# Spin‐Flip‐Restricted Multiple‐Resonance Emitters for Extended Device Lifetime in Indolocarbazole‐Based Blue Organic Light‐Emitting Diodes

**DOI:** 10.1002/advs.202405604

**Published:** 2024-08-29

**Authors:** Jihoon Kang, Ha Lim Lee, Soon Ok Jeon, Hye Jin Bae, Seung Chan Kim, Seungwon Han, Jun Yeob Lee

**Affiliations:** ^1^ School of Chemical Engineering Sungkyunkwan University 2066, Seobu‐ro, Jangan‐gu Suwon Gyeonggi 16419 Republic of Korea; ^2^ Samsung Advanced Institute of Technology Samsung Electronics Co., Ltd 130 Samsung‐ro, Yeongtong‐gu Suwon Gyeonggi 16678 Republic of Korea; ^3^ Department of Display Convergence Engineering Sungkyunkwan University 2066, Seobu‐ro, Jangan‐gu Suwon Gyeonggi 16419 Republic of Korea; ^4^ SKKU Institute of Energy Science and Technology Sungkyunkwan University 2066, Seobu‐ro, Jangan‐gu Suwon Gyeonggi 16419 Republic of Korea

**Keywords:** blue emitter, device lifetime, fluorescence, multiple‐resonance effect, narrow emission

## Abstract

In this study, a multiple‐resonance (MR) core structure is developed with a spin‐flip‐restricted emission mechanism based on a fused indolo[3,2,1‐*jk*]carbazole (ICz) framework as emitters to improve the lifetime of blue organic light‐emitting diodes. The molecular skeleton modulation approach applied to the conjugated *π*‐system effectively stabilizes the triplet energy of the fused ICz emitters and narrows the full‐width‐at‐half maximum (<20 nm). In addition, the emitters exhibit higher exciton stability than conventional boron‐based MR emitters. The fused ICz‐based blue fluorescent device exhibits a high external quantum efficiency of 7.2%, a blue index of 68.6 cd A^−1^ at a Commission internationale de l'éclairage y coordinate (CIE_y_) of 0.075, and a device lifetime 1.8 times longer than that of a boron‐based emitter. In addition, a phosphor‐sensitized fluorescent device based on the ICz emitter exhibited an improved external quantum efficiency of 20.6% with a CIE_y_ coordinate of 0.076.

## Graphical abstract

A multiple‐resonance thermally activated delayed fluorescence emitter with a spin‐flip‐restricted emission mechanism based on a fused indolo[3,2,1‐*jk*]carbazole (ICz) framework is developed as an emitter to improve the lifetime of blue organic light‐emitting diodes.

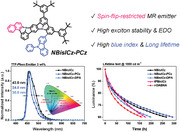

## 1 Introduction

Molecular design approaches using multiple‐resonance (MR) effects have attracted significant attention in the development of high performance blue emitters with high photoluminescence quantum yield (PLQY) and narrow emission bandwidth.^[^ ^1^, ^2^, ^3^, ^4^, ^5^, ^6^ ^]^ The molecular design concept based on the MR effect was established and rationalized by Hatakeyama's group in 2016.^[^ ^1^ ^]^ Unique characteristics are induced when heteroatoms (e.g., boron and nitrogen) are appropriately arranged in an alternating manner on a polycyclic aromatic hydrocarbon (PAH) structure through the electron push–pull effect. The precisely controlled electron density of MR compounds on an atomic scale separates the highest occupied molecular orbital (HOMO) and lowest unoccupied molecular orbital (LUMO), resulting in short‐range charge transfer (CT). Combining the unique electronic structure and rigid molecular geometry of MR‐based PAH compounds, high PLQY, small Stokes shift, and narrow emission spectrum can be achieved. In addition, the atomically separated HOMO–LUMO distributions result in a small singlet–triplet energy gap (Δ*E*_ST_) for thermally activated delayed fluorescence (TADF) characteristics.^[^ ^7^, ^8^, ^9^, ^10^ ^]^ Thus, MR‐TADF emitters can up‐convert 75% of triplet excitons generated in electroluminescence (EL) into singlet excitons through reverse intersystem crossing (RISC) and achieve 100% internal quantum efficiency.^[^ ^11^ ^]^

Since the revolutionary report of 5,9‐diphenyl‐5,9‐dihydro‐5,9‐diaza‐13b‐boranaphtho[3,2,1‐*de*]anthracene (DABNA‐1), numerous high‐color purity and high‐efficiency MR‐TADF organic light‐emitting diodes (OLEDs) have been reported with a high external quantum efficiency (EQE) of over 30%.^[^ ^12^, ^13^, ^14^, ^15^, ^16^, ^17^, ^18^, ^19^, ^20^, ^21^, ^22^, ^23^, ^24^, ^25^, ^26^ ^]^ However, ensuring long‐term stability in MR‐TADF OLEDs remains a challenge because of the long triplet exciton lifetime of MR‐TADF emitters. When a triplet exciton harvesting emission mechanism is employed, triplet exciton‐triggered quenching (e.g., triplet–triplet annihilation (TTA) and triplet–polaron annihilation (TPA)) significantly contributes to device degradation due to hot excitons and polarons, thereby shortening the device operational lifetime.^[^ ^27^, ^28^, ^29^ ^]^ For MR‐TADF emitters, the small Δ*E*_ST_ allows the up‐conversion of triplet excitons with an exciton lifetime of approximately microseconds, thereby degrading the devices during long‐term electrical driving. Therefore, it is necessary to conserve the narrow emission bandwidth of the MR emitters and suppress the TADF process by energetically stabilizing the triplet excited states.

Previously, we reported fused indolo[3,2,1‐*jk*]carbazole (ICz)‐based MR frameworks without boron in the PAH structure.^[^ ^30^, ^31^ ^]^ Among them, an indolo[3,2,1‐*jk*]indolo[1′,2′,3′:1,7]indolo[3,2‐*b*]carbazole (BisICz) framework designed through a unique *para*‐oriented dimeric fusion of ICz units exhibited a strong monoatomic MR effect. The intensified short‐range CT of BisICz resulted in Δ*E*_ST_ of 0.31 eV or less, thereby allowing TADF behavior with excellent deep blue color. The TADF behavior of BisICz derivatives is attributed to the second‐order spin‐vibronic coupling (SVC)‐assisted RISC process, known as the SVC‐TADF mechanism, in which high‐lying triplet states participate in the triplet state (T_1_)‐to‐singlet state (S_1_) spin‐flip.^[^ ^16^, ^31^, ^32^ ^]^ In the device application, BisICz derivatives recorded a Commission internationale de l'éclairage (CIE) y coordinate of 0.05 and a high EQE of 23.1%, indicating that the BisICz framework is excellent for deep blue emitters. However, SVC‐TADF emitters suffer from severe efficiency roll‐off and short device lifetime due to the slow RISC process. Therefore, there is a need for an MR molecular design descriptor that can address the long‐term stability issue of MR emitters.

Herein, we propose a novel spin‐flip‐restricted ICz framework named 10,13‐bis(3,5‐di‐*tert*‐butylphenyl)‐indolo[3,2,1‐*jk*]benzo[4′,5′]indolo[3′,2′,1′:7,1]indolo[3,2‐*b*]carbazole (NBisICz) for stable MR emitters and, in turn, a long device lifetime. NBisICz was designed by employing a naphthalene unit in the expanded *π*‐conjugation system of BisICz to obtain a large Δ*E*_ST_ and a triplet–triplet energy gap (Δ*E*_TT_), thereby deactivating the SVC‐TADF mechanism. Furthermore, two *ortho*‐substituted NBisICz derivatives, 10,13‐bis(3,5‐di‐*tert*‐butylphenyl)‐15‐(9‐phenyl‐carbazol‐3‐yl)‐indolo[3,2,1‐*jk*]benzo[4′,5′]indolo[3′,2′,1′:7,1]indolo[3,2‐*b*]carbazole (NBisICz–PCz) and 10,13‐bis(3,5‐di‐*tert*‐butylphenyl)‐*N* ^15^,*N* ^15^‐diphenyl‐indolo[3,2,1‐*jk*]benzo[4′,5′]indolo[3′,2′,1′:7,1]indolo[3,2‐*b*]carbazol‐15‐amine (NBisICz–DPA), were developed to enhance the photophysical and EL characteristics. The rigid and fully conjugated core structure of NBisICz enabled a narrow full‐width‐at‐half‐maximum (FWHM) (<30 nm), appropriate singlet energy (*E*_S_) (2.75–2.79 eV) for blue emission, and low triplet energy (*E*_T_) for suppressed TADF. The NBisICz–PCz‐based MR device exhibited a CIE_y_ of 0.075, a small FWHM of 25 nm, a high EQE of 7.2%, and a device lifetime 1.8 times longer than that of the state‐of‐the‐art boron–nitrogen‐based MR emitters. In addition, a phosphor‐sensitized fluorescent (PSF) device based on the ICz emitter exhibited an improved EQE of 20.6% with a CIE_y_ coordinate of 0.076.

## 2 Results and Discussion

### 2.1 Molecular Design and Simulation

**Figure 1:**
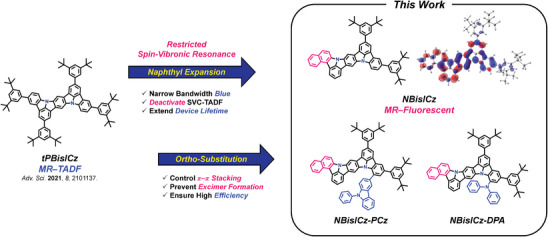
Molecular design strategy for the NBisICz core structure and its derivatives (blue: highest occupied molecular orbital (HOMO); red: lowest unoccupied molecular orbital (LUMO)).

The BisICz derivatives reported in our previous study exhibited a high EQE of up to 23.1% owing to the activated SVC‐TADF mechanism. However, their small rate constant of RISC (*k*_RISC_) caused serious efficiency roll‐off and unstable device operation despite the high EQE due to the long exciton lifetime. To overcome this problem, we propose a molecular design strategy that eliminates the delayed fluorescence component originating from a fused ICz framework (**Figure** **1**). The NBisICz core structure has an expanded *π*‐conjugated structure incorporating a naphthyl unit into the BisICz core to manage the *E*_T_. The naphthyl‐embedded backbone structure of NBisICz can stabilize the T_1_ state and deactivate the SVC‐TADF mechanism. Two di‐*tert*‐butylphenyl units of the NBisICz core were attached to induce bathchorimics shift of *E*_S_, to block intermolecular interactions, and obtain a high horizontal emitting dipole orientation ratio (*Θ*_Hor_).^[^ ^31^ ^]^ Furthermore, 9‐phenylcarbazole and diphenylamine were introduced to the backbone structures of NBisICz–PCz and NBisICz–DPA as substituents, resulting in additional blocking groups.^[^ ^33^ ^]^ The molecular orbital distribution of the NBisICz core demonstrates a unique MR feature with an atomically separated HOMO/LUMO pattern up to the naphthyl unit. Therefore, the expanded *π*‐system of NBisICz would exhibit MR characteristics.

**Figure 2:**
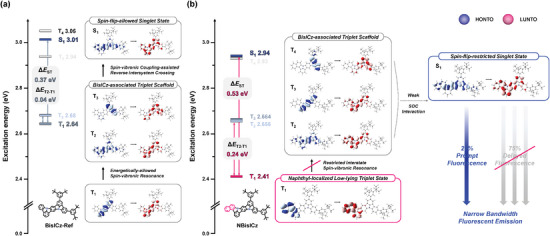
Emission mechanism of a) spin‐vibronic coupling‐thermally activated delayed fluorescence based on BisICz‐Ref and b) spin‐flip‐restricted fluorescence based on the NBisICz structure (blue: highest occupied natural transition orbital (HONTO); red: lowest unoccupied natural transition orbital (LUNTO)).

Ab initio calculations based on density functional theory (DFT) were performed to predict the material characteristics of the proposed NBisICz. Because NBisICz was derived from BisICz, the calculation results of NBisICz were compared with those of the BisICz derivative, denoted as BisICz‐Ref, with the same skeleton except for the *π*‐extended aromatic structure. **Figure** **2** shows a schematic of two emission mechanisms (SVC‐TADF and spin‐flip‐restricted fluorescence) predicted by the excited state simulation of BisICz‐Ref and NBisICz, including the vertical excitation energies and natural transition orbital (NTO) distributions. Table S1 (Supporting Information) lists the vertical excitation energies of the excited states and the reorganization energy (λ_Reorg_) of the fused ICz frameworks, and Figure S21 (Supporting Information) shows the NTO distributions of BisICz‐Ref, NBisICz, and its derivatives.

BisICz‐Ref and NBisICz showed similar highest occupied NTO (HONTO)/lowest unoccupied NTO (LUNTO) distributions in the S_1_ state. BisICz‐Ref showed an alternating HONTO/LUNTO distribution exhibiting the MR effect of the fused ICz framework, which was extended to the additional aromatic unit of NBisICz, indicating that the extended aromatic structure does not affect the MR characteristics. However, the λ_Reorg_ of NBisICz slightly increased compared with that of BisICz‐Ref (from 0.2315 to 0.2528 eV), which is attributed to the asymmetric contribution of the fused ICz structure to the MR electronic structure. This shows that NBisICz exhibits a slightly increased emission bandwidth.^[^ ^34^, ^35^ ^]^

In general, MR‐type emitters exhibit the same local excited (LE) state nature in the S_1_ and T_1_ states, which prohibits direct spin‐orbit coupling (SOC) (i.e., T_1_–S_1_ spin crossover) because of the El‐Sayed rule.^[^ ^36^ ^]^ The SVC‐TADF emission mechanism has been proposed to rationalize the RISC process of MR‐type emitters. This mechanism is determined by i) Δ*E*_ST_ and the vibrational coupling between T_1_ and high‐lying T_n_ states and ii) appropriate SOC matrix element values between the S_1_ and T_n_ states.^[^ ^16^, ^31^, ^32^ ^]^ Therefore, it is necessary to energetically restrict the vibronic resonance between the T_1_ and T_n_ states by stabilizing the T_1_ state to eliminate the delayed fluorescence component of MR‐type emitters. Figure 2a,b compare the calculated excited states of BisICz‐Ref and NBisICz, illustrating the spin‐flip‐allowed electronic structure of BisICz‐Ref by SVC‐TADF and an energetically stabilized T_1_ state of NBisICz deactivating the spin‐vibronic resonance through Δ*E*_ST_ of 0.53 eV and Δ*E*_T2‐T1_ of 0.24 eV. SOC calculations were further performed to confirm the effect of excited states, and the results are summarized in Table S2 (Supporting Information). NBisICz showed small SOC matrix element values and large Δ*E*_Tn‐T1_ values simultaneously, indicating that it exhibits fluorescence without delayed fluorescence.

**Figure 3:**
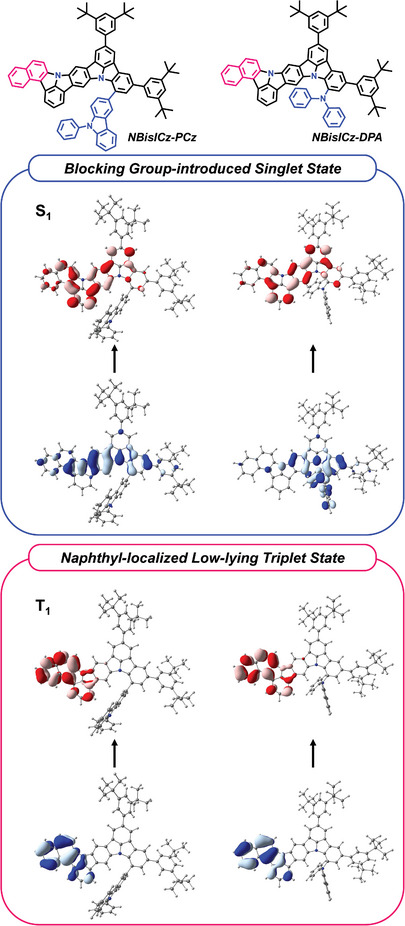
Molecular structure and associated NTO distributions for the first singlet and triplet excited states of NBisICz–PCz (left) and NBisICz–DPA (right) (blue: HONTO; red: LUNTO).

In the case of NBisICz–PCz and NBisICz–DPA, the T_1_, T_2_, and T_3_ state NTO distributions and their excitation energies were almost identical with those of NBisICz despite the presence of an additional electron‐donating group. Therefore, they are predicted to follow the same emission mechanism as that of NBisICz. As shown in **Figure** **3**, the 9‐phenylcarbazole substituent contributed slightly to the NTO of NBisICz–PCz, resulting in almost the same excited state energy as that of NBisICz. However, the strong electron‐donating property of the diphenylamine substituent greatly contributed to the HONTO of the S_1_ state and induced long‐range CT. Among the three emitters, NBisICz–DPA showed the largest λ_Reorg_ of 0.3195 eV, the most stabilized S_1_ energy, and the smallest Δ*E*_ST_ because of the intramolecular CT characteristic. NBisICz–PCz would exhibit an LE characteristic dominated by short‐range CT, and NBisICz–DPA would have hybridized LE and CT characteristics.

As the two blocking groups are intended to control the intermolecular distance by increasing the bulkiness, the molecular cubic volume of the emitters was calculated at the optimized S_0_ geometry (Figure S22 and Table S3, Supporting Information). The molecular cubic volumes of NBisICz, NBisICz–PCz, and NBisICz–DPA were 2.778, 3.925, and 3.711 nm^3^, respectively. The NBisICz–PCz emitter with a bulky 9‐phenylcarbazole blocking group exhibited efficient intermolecular interaction blocking.

### 2.2 Material Characterization

Scheme S1 (Supporting Information) shows the synthetic scheme and molecular structures of NBisICz, NBisICz–PCz, and NBisICz–DPA. Details of the synthetic method, nuclear magnetic resonance data, and mass spectroscopy data are provided in the Synthesis Section of the Supporting Information. Starting from naphthalen‐2‐amine, 1,3‐dibromo‐2‐fluorobenzene, and 3,6‐bis(3,5‐di‐*tert*‐butylphenyl)−9*H*‐carbazole, functionalized carbazole‐derived intermediates were synthesized to regioselectively cyclize the PAH structures. NBisICz and its derivatives were synthesized through sequential nucleophilic substitution, palladium‐catalyzed ring‐closing, palladium‐catalyzed Miyaura borylation, bromination, Suzuki–Miyaura cross‐coupling, and Ullmann amination‐based cyclization reactions. The synthesized products were purified by column chromatography, reprecipitation, and train sublimation before material and device characterization.

**Figure 4:**
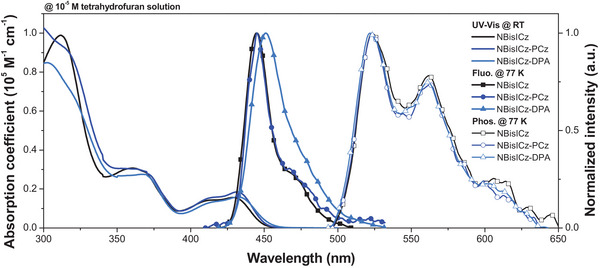
Solution PL analysis of emitters sampled in 10^−5^ M tetrahydrofuran. Ultraviolet–visible (UV–Vis) absorption spectra measured at room temperature (RT) and fluorescence (Fluo.) and phosphorescence (Phos.) spectra measured at 77 K of NBisICz (black), NBisICz–PCz (blue), and NBisICz–DPA (sky blue).

We analyzed the photophysical, electrochemical, and thermal properties of NBisICz, NBisICz–PCz, and NBisICz–DPA. **Table** **1** lists the characterization data of the three emitters, and **Figure** **4** shows the ultraviolet–visible (UV–Vis) absorption spectra and photoluminescence (PL) emission spectra. Both spectra were obtained at room temperature and 77 K using a 1.0 × 10^−5^ m diluted tetrahydrofuran solution. All molecules exhibited similar absorption patterns with strong local *π–π* ^*^ transition at short wavelengths and weak short‐range CT transitions corresponding to HOMO–LUMO transitions at long wavelengths. This confirms that the three molecules share the photophysical transition characteristics of fused ICz‐based MR emitters. The peak absorption wavelength (λ_abs_) for the HOMO–LUMO transition/log molar absorption coefficient (log*ε*) of NBisICz, NBisICz–PCz, and NBisICz–DPA were 429 nm/4.19, 432 nm/4.27, and 432 nm/4.20, respectively, indicating that the three emitters have similar light absorption properties. The optical bandgaps (*E*_opt_) of NBisICz, NBisICz–PCz, and NBisICz–DPA at the onset energy of the absorption spectrum were 2.76, 2.75, and 2.73 eV, respectively.

**Table 1:** Material characterization data of NBisICz and its derivatives.

	*E*_HOMO_ ^a)^	*E*_LUMO_ ^b)^	*E*_opt_ ^c)^	λ_abs_	log*ε*	λ_PL_ ^d)^	FWHM	*E*_s_ ^d)^	*E*_T_ ^d)^	Δ*E*_ST_ ^e)^	Stokes shift^f)^
	[eV]	[eV]	[eV]	[nm]		[nm]	[nm]	[eV]	[eV]	[eV]	[nm]
NBisICz	−5.68	−2.92	2.76	429	4.19	445	19.5	2.79	2.37	0.42	16
NBisICz–PCz	−5.65	−2.90	2.75	432	4.27	445	18.6	2.79	2.37	0.42	13
NBisICz–DPA	−5.71	−2.98	2.73	432	4.20	451	29.1	2.75	2.37	0.37	19

^a)^ Measured by cyclic voltammetry;

^b)^ Estimated from the *E*_opt_ and HOMO levels;

^c)^ Value at the onset position of the UV–Vis absorption spectrum;

^d)^ Low‐temperature fluorescence/phosphorescence spectra measured in diluted tetrahydrofuran solution at 77 K without/with 1 ms delay. Values at peak position;

^e)^ Difference between *E*_S_ and *E*_T_;

^f)^ Difference between λ_abs_ and λ_PL_.

The PL characteristics of the emitters were determined by low‐temperature fluorescence (LTFl) spectroscopy. Peak wavelength (λ_PL_)/FWHM/Stokes shifts of 445/19.5/16, 445/18.6/13, and 451/29.1/19 nm were observed for NBisICz, NBisICz–PCz, and NBisICz–DPA, respectively. All emitters showed typical MR features characterized by a FWHM of <30 nm and a Stokes shift of <20 nm because of the structural rigidity of the sp^2^ nitrogen‐based fused ICz framework. Although the photophysical properties of NBisICz–PCz and NBisICz were comparable, NBisICz–DPA showed a red shift in the emission peak, accompanied by spectral broadening because of intramolecular CT from the strong electron‐donating diphenylamine unit. The photophysical properties are consistent with the DFT calculation results, and the FWHM order agrees with the tendency of λ_Reorg_. The *E*_T_ obtained from the low‐temperature phosphorescence (LTPh) spectra was 2.37 eV for all three molecules, indicating that the naphthyl‐localized T_1_ state dominantly determines the *E*_T_ of the molecule regardless of the blocking groups. Because the BisICz‐derived emitters exhibited *E*_T_ of ≈2.55 eV, the design strategy of the NBisICz core structure is effective in stabilizing *E*_T_.^[^ ^31^, ^33^ ^]^ The NBisICz, NBisICz–PCz, and NBisICz–DPA emitters exhibited significantly increased Δ*E*_ST_ values of 0.42, 0.42, and 0.37 eV, respectively.

The HOMO and LUMO of the samples were determined using an electrochemical method. The HOMO was measured using cyclic voltammetry, and the LUMO was estimated from HOMO and *E*_opt_. As shown in Figure S23 (Supporting Information), NBisICz, NBisICz–PCz, and NBisICz–DPA exhibited similar HUMO (−5.68, −5.65, and −5.71 eV, respectively) and LUMO levels (−2.92, −2.90, and −2.98 eV, respectively).

The intramolecular CT properties of the three molecules were confirmed from the solvent‐dependent PL spectra (Figure S24, Supporting Information). As solvent matrices, *n*‐hexane, toluene, tetrahydrofuran, and methylene chloride with different dielectric constants were selected. When the matrix was changed from low polarity (*n*‐hexane) to high polarity (methylene chloride), the peak wavelengths of NBisICz, NBisICz–PCz, and NBisICz–DPA redshifted by ≈10 nm. However, there was a significant difference in the spectral broadening depending on the solvent polarity. The FWHM of NBisICz and NBisICz–PCz increased by up to 6 nm, whereas that of NBisICz–DPA broadened up to 19 nm. These results show that NBisICz and NBisICz–PCz exhibit poor intramolecular CT, whereas the S_1_ excited state of NBisICz–DPA exhibits relatively large long‐range CT, which is attributed to the strong electron‐donating strength of diphenylamine. These results agree well with the NTO distributions predicted by the DFT calculations.

**Figure 5:**
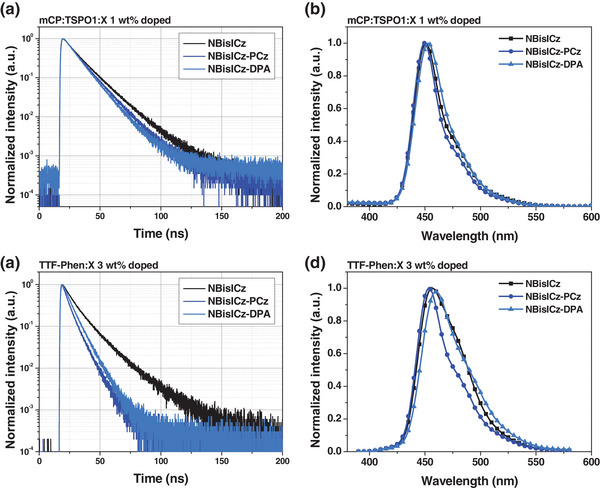
Transient PL decay curves of NBisICz (black), NBisICz–PCz (blue), and NBisICz–DPA (sky blue) a) 1 wt% doped mCP:TSPO1 and c) 3 wt% doped TTF‐Phen films. Solid PL spectra of NBisICz (black), NBisICz–PCz (blue), and NBisICz–DPA (sky blue) b) 1 wt% doped mCP:TSPO1 and d) 3 wt% doped TTF‐Phen films.

Solid PL analysis was performed to analyze the radiative‐transition‐related photophysical properties of the fused ICz emitters. **Table** **2** and **Figure** **5** show the results of the solid PL analysis of the emitters. Transient PL and PLQY analyses were conducted to characterize the dynamic electronic transition behavior of the emitters using 1,3‐di(9*H*‐carbazol‐9‐yl)benzene (mCP):diphenyl(4‐(triphenylsilyl)phenyl)phosphine oxide (TSPO1) host. The mCP:TSPO1 host was selected to harvest triplet excitons for radiative transition. However, only conventional fluorescence in the nanosecond range was detected, indicating the absence of the TADF mechanism in the three emitters. The spin‐flip‐restricted emission mechanism predicted by quantum chemical calculations was verified in the NBisICz derivatives. The fitted fluorescence decay time (*τ*_F_) of NBisICz, NBisICz–PCz, and NBisICz–DPA was 14.0, 12.2, and 11.2 ns, respectively, and the measured PLQYs of the doped thin films were 66, 70, and 72%. The relatively low PLQY of the NBisICz emitter is attributed to intermolecular interaction‐induced exciton quenching because of the lack of blocking groups. Among the three emitters, NBisICz–PCz showed the narrowest emission spectrum with an FWHM of 28.1 nm. The rate constants of the radiative singlet emissive transition (*k*_r_^S^) of the films were 2.43 × 10^7^, 2.70 × 10^7^, and 2.50 × 10^7^ s^−1^, respectively. Appropriate functional groups introduced into the MR backbone structure enabled a more effective radiative transition.

**Table 2:** Photophysical parameters of NBisICz‐, NBisICz–PCz‐, and NBisICz–DPA‐doped films measured in mCP:TSPO1 and TTF‐Phen host systems.

Host	Doping concentration	Emitter	λ_PL_ ^a)^, ^b)^	FWHM	*E*_s_ ^a)^	PLQY ^b)^	*τ*_F_ ^c)^	*k*_r_^S^ ^d)^	*k*_ISC_ ^e)^	*Θ*_Hor_ ^f)^
			[nm]	[nm]	[eV]	[%]	[ns]	[10^7^ s^−1^]	[10^7^ s^−1^]	[%]
mCP:TSPO1	1 wt%	NBisICz	451	31.8	2.75	66	14.0	4.71	2.43	–
		NBisICz–PCz	450	28.1	2.75	70	12.2	5.49	2.70	–
		NBisICz–DPA	453	33.0	2.74	72	11.2	6.43	2.50	–
TTF‐Phen	3 wt%	NBisICz	457	47.0	2.71	63	12.6	5.00	2.94	90
		NBisICz–PCz	454	35.9	2.73	64	6.3	10.2	5.75	92
		NBisICz–DPA	460	45.9	2.70	64	7.2	8.91	5.01	91

^a)^ Value at the peak position;

^b)^ Absolute PL quantum yield in a nitrogen atmosphere;

^c)^ Fluorescence decay time;

^d)^ Rate constant of radiative singlet emissive transition;

^e)^ Rate constant of intersystem crossing;

^f)^ Horizontal emitting dipole orientation ratio;

The same analysis was performed for the TTF‐Phen (9‐(3‐(phenanthren‐9‐yl)phenyl)−10‐phenylanthracene) matrix, which is a typical anthracene‐based triplet–triplet fusion (TTF) host. TTF is a suitable host material for stable blue fluorescent OLEDs, and its small *E*_T_ effectively deactivates triplet excitons formed in PL systems. Herein, the doping concentration of the deposited films was set to 3 wt%. The λ_PL_ of each film was 457, 454, and 460 nm, showing an ≈10 nm redshift accompanied by spectral broadening compared to that of solution PL analysis. This is attributed to solid state *π*–*π* stacking between hosts and emitters. Unlike NBisICz–PCz, which exhibited excellent intermolecular blocking performance, NBisICz showed a broadened FWHM of more than 45 nm. PL and transient PL analyses revealed that excimers were formed in the NBisICz film because of the severe *π*–*π* stacking. The *τ*_F_ of NBisICz–PCz was 6.3 ns, whereas that of NBisICz was 12.6 ns, indicating a significantly slow decay component. Intermolecular CT‐originated emissive states, such as excimers, exhibit relatively long decay times compared with monomers, and similar results were observed in the radiative properties of a TTF‐Phen‐hosted NBisICz film.^[^ ^33^ ^]^ In the case of PLQY, the three emitters exhibited similar values (≈64%). In addition, the *k*_r_^S^ of NBisICz–PCz was 10.2 × 10^7^ s^−1^, which was higher than that of NBisICz and NBisICz–DPA (5.00 × 10^7^ and 8.91 × 10^7^ s^−1^, respectively).

**Figure 6:**
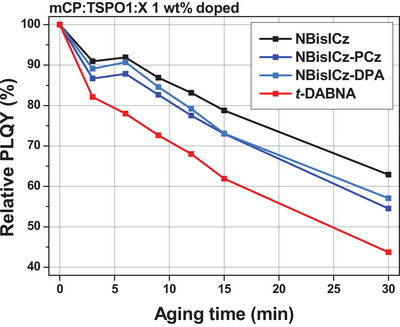
Changes in the relative photoluminescence quantum yield (PLQY) of NBisICz, NBisICz–PCz, NBisICz–DPA, and *t*‐DABNA with aging time.

To investigate the intrinsic stability of the emitters, photostability tests were conducted by measuring the PLQY of the emitter‐doped films at different UV irradiation times (**Figure** **6**). As a solid matrix, mCP:TSPO1 was selected, and the doping concentration of the emitters was set to 1 wt%. The high‐triplet‐energy host system allowed triplet exciton confinement into the emitter, and the low emitter concentration minimized the concentration‐dependent triplet–triplet annihilation. One of the representative MR emitters, 2,12‐di‐*tert*‐butyl‐5,9‐bis(4‐(*tert*‐butyl)phenyl)−5,9‐dihydro‐5,9‐diaza‐13b‐boranaphtho[3,2,1‐*de*]anthracene (*t*‐DABNA)^[^ ^37^ ^]^ was selected as a reference to compare the PL degradation of the samples. As shown in Figure S25 (Supporting Information), all doped films maintained the intrinsic narrow emission spectrum of each emitter even after UV exposure. After 30 min UV aging, NBisICz, NBisICz–PCz, and NBisICz–DPA exhibited relative PLQY values of 63%, 55%, and 57%, respectively, indicating that the PL stability of the NBisICz–PCz and NBisICz–DPA films was slightly lower than that of the NBisICz film. Because the NBisICz derivatives exhibited the same *E*_T_ (2.37 eV), the difference in PL stability can be attributed to the structural differences caused by the introduction of substituents. Unlike the three emitters, the *t*‐DABNA film showed a significantly reduced relative PLQY value of 44%. These results are attributed to the high *E*_T_ value for *t*‐DABNA (2.63 eV), the long triplet exciton lifetime due to TADF, and the weak C–N bond in the six‐membered PAH structure. The NBisICz derivatives exhibited better exciton stability than *t*‐DABNA.

To evaluate the *Θ*_Hor_ of the films with a TTF‐Phen host, angle‐dependent PL analysis was performed for NBisICz, NBisICz–PCz, and NBisICz–DPA (Figure S26, Supporting Information). The fitted *Θ*_Hor_ values of the fused ICz framework were 90%, 92%, and 91%, respectively. The multiply fused structure of the fused ICz framework and the expanded molecular linearity by substituting di‐*tert*‐butylphenyl groups provided a high *Θ*_Hor_ of over 90%.

Thermogravimetric analysis was conducted to evaluate the thermal stability of the emitters (Figure S27, Supporting Information). The measured decomposition temperatures (*T*_d_) of NBisICz, NBisICz–PCz, and NBisICz–DPA defined as the temperature at 5% weight loss were 525, 541, and 520 °C, respectively. The rigid sp^2^ nitrogen‐based fused ICz structure showed a high *T*_d_ of over 500 °C. Among the three emitters, the large molecular weight and stable C–C‐bond‐based NBisICz–PCz provided the highest *T*_d_.

### 2.3 Device Characterization

NBisICz, NBisICz–PCz, and NBisICz–DPA were embedded as fluorescent emitters in an anthracene‐based TTF host. The fabricated device structure was ITO (50 nm)/BCFN:HATCN (40 nm, 30 wt%)/BCFN (10 nm)/oCBP (10 nm)/TTF‐Phen:emitter (30 nm, X wt%)/SBF‐Trz (5 nm)/ZADN (20 nm)/LiF/Al (1.5 nm/200 nm), where ITO is indium tin oxide, BCFN is *N*‐([1,1′‐biphenyl]−4‐yl)−9,9‐dimethyl‐*N*‐(4‐(9‐phenyl‐9*H*‐carbazol‐3‐yl)phenyl)−9*H*‐fluoren‐2‐amine, HATCN is hexaazatriphenylenehexacarbonitrile, oCBP is 2,2′‐di(9*H*‐carbazol‐9‐yl)−1,1′‐biphenyl, SBF‐Trz is 2‐(9,9′‐spirobi[fluoren]−4‐yl)−4,6‐diphenyl‐1,3,5‐triazine, and ZADN is 2‐(4‐(9,10‐di(naphthalen‐2‐yl)anthracen‐2‐yl)phenyl)−1‐phenyl‐1*H*‐benzo[*d*]imidazole. Furthermore, NBisICz–PCz and NBisICz–DPA were embedded as terminal emitters in a PSF system. The fabricated device structure is ITO (50 nm)/PEDOT:PSS (40 nm, 30 wt%)/TAPC (10 nm)/TCTA (5 nm)/mCP (5 nm)/3‐CzPB:PtON7‐dtb:NBisICz–PCz or NBisICz–DPA (25 nm, 10 wt%:2 wt%)/TSPO1 (25 nm)/LiF/Al (1.5 nm/200 nm), where TAPC is 4,4′‐(cyclohexane‐1,1‐diyl)bis(*N*,*N*‐di‐*p*‐tolylaniline), TCTA is tris(4‐(9*H*‐carbazol‐9‐yl)phenyl)amine, 3‐CzPB is 2,6‐bis(3‐(9*H*‐carbazol‐9‐yl)phenoxy)benzonitrile,^[^ ^38^ ^]^ and PtON7‐dtb is *N*‐heterocylic tetradentate Pt(II) complex reported from Jian Li's group.^[^ ^39^ ^]^ To allow efficient Förster energy transfer in NBisICz derivatives with a large *E*_S_, PtON7‐dtb, a narrow‐emitting deep blue Pt(II) complex, was selected as a phosphor sensitizer. 3‐CzPB is a large‐*E*_T_ bipolar host and was selected as a single host material because it exhibits excellent charge‐balancing characteristics. Figure S28 (Supporting Information) shows the energy level diagram of two device architectures, and Figure S29 (Supporting Information) shows the chemical structure of the materials. Furthermore, 2,5,11,14‐tetrakis(3,5‐di‐*tert*‐butylphenyl)‐indolo[3,2,1‐*jk*]indolo[1′,2′,3′:1,7]indolo[3,2‐*b*]carbazole (tPBisICz) and *t*‐DABNA were used as reference emitters. Because all emitters have similar HOMO and LUMO levels, device performance is mostly determined by material characteristics rather than device parameters. For the TTF devices, the doping concentration of the emitters was varied (1, 3, and 5 wt%) to investigate concentration‐dependent device performance. The doping concentration of the terminal emitters in the PSF device was set to 2 wt% to allow complete energy transfer in the system.

**Figure 7:**
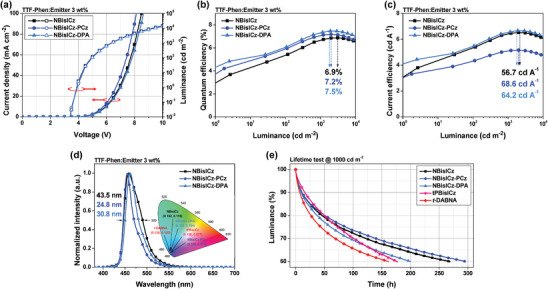
Device performances of 3 wt% emitter doped blue MR–TTF devices. a) Current density–voltage–luminance curves. b) External quantum efficiency–luminance curves including maximum value. c) Current efficiency–luminance curves including maximum blue index. d) Normalized electroluminescence spectra (inset: corresponding CIE color coordinates). e) Luminance–lifetime curves (black: NBisICz; blue: NBisICz–PCz; sky blue: NBisICz–DPA; pink: tPBisICz; red: *t*‐DABNA).

**Figure** **7**, Figures S30–S32 (Supporting Information) and **Table** **3** show the operational characteristics of the TTF devices. The fabricated MR–TTF OLEDs with a 3 wt% doping concentration showed optimum device performance in terms of efficiency, color purity, and device lifetime. All NBisICz‐derived emitters exhibited low EQE at a 1 wt% doping concentration due to incomplete energy transfer from the host to the MR emitters (Figure S30j–l, Supporting Information). The device with 3 and 5 wt% doping concentrations showed complete energy transfer, improving the EQE and current efficiency (CE). However, at high doping concentrations, the spectra shifted and broadened with increasing concentration. In particular, the peak wavelength of the NBisICz devices shifted from 454 to 469 nm, and the FWHM increased from 26.5 to 48.4 nm because of the strong *π*–*π* interaction through the planar core structure. They also showed relatively low EQE compared to other devices as can be predicted from low PLQY of the NBisICz emitter. At a 3 wt% doping concentration, the NBisICz–PCz and NBisICz–DPA devices exhibited EQEs of 7.2% and 7.5%, FWHM values of 24.8 and 30.8 nm, CE values of 5.2 and 6.7 cd A**^−^** ^1^, and CIE_y_ coordinates of 0.075 and 0.104, respectively, indicating excellent color purity and high efficiency. This is attributed to the superior intermolecular blocking function. The relatively low CE of NBisICz–PCz is attributed to the blue shift in the color coordinates. Nevertheless, among the three emitters, NBisICz–PCz showed the best color purity and the highest blue index (defined as CE divided by CIE_y_) of 68.6 cd A**^−^** ^1^. Moreover, the spectral shift and broadening were suppressed in the NBisICz–PCz devices.

**Table 3:** Device performance of blue MR–TTF and PSF OLEDs.

	Type	Doping conc. [wt%]	*V*_d_ ^a)^ [V]	λ_EL_ ^b)^ [nm]	FWHM ^a)^ [nm]	CIE (x, y)^a)^	EQE (%)		CE_Max_ [cd A^−1^]	Blue index [cd A^−1^]	LT_60_ ^c)^ [h]
							Max	1000 cd m^−2^			
NBisICz	TTF	1	6.7	454	26.5	(0.141, 0.077)	5.6	5.4	4.1	53.0	–
		3	6.3	458	43.5	(0.132, 0.115)	6.9	6.7	6.5	56.7	269.0
		5	6.1	469	48.4	(0.127, 0.148)	7.1	6.9	7.9	53.8	–
NBisICz–PCz	TTF	1	6.8	453	22.2	(0.142, 0.066)	6.7	6.6	4.4	66.0	–
		3	6.3	455	24.8	(0.139, 0.075)	7.2	7.1	5.2	68.6	296.2
		5	6.5	457	29.7	(0.137, 0.088)	7.5	7.4	6.0	68.3	–
	PSF	2	7.2	453	22.0	(0.143, 0.076)	18.8	5.5	16.6	218.8	–
NBisICz–DPA	TTF	1	6.2	458	28.9	(0.136, 0.085)	6.7	6.3	5.2	61.1	–
		3	6.4	462	30.8	(0.132, 0.104)	7.5	7.3	6.7	64.2	200.1
		5	6.1	463	31.9	(0.129, 0.115)	7.5	7.4	7.2	62.5	–
	PSF	2	6.6	454	27.5	(0.141, 0.085)	20.6	7.9	19.1	224.6	–
tPBisICz	TTF	3	6.7	456	25.3	(0.138, 0.077)	6.6	6.4	4.8	62.4	177.2
*t*‐DABNA	TTF	3	6.8	464	25.2	(0.126, 0.100)	8.2	8.2	6.9	68.8	161.7

^a)^ Value at 1000 cd m^−2^;

^b)^ Value at peak position;

^c)^ Device lifetime at an initial luminance of 1000 cd m^−2^ until reaching 60% relative luminance.

Constant‐current density lifetime tests were performed to evaluate the operational stability of the NBisICz derivatives (Figure 7e). The measured device lifetime up to 60% of the initial luminance of 1000 cd m^−2^ (LT_60_) was 269.0, 296.2, and 200.1 h for NBisICz, NBisICz–PCz, and NBisICz–DPA, respectively. The tPBisICz/*t*‐DABNA control devices recorded a EQE of 6.6/8.2% and a blue index of 62.4/68.8 cd A^–1^ but a relatively short LT_60_ of 177.2/161.7 h, respectively. Overall, the NBisICz derivatives showed a similar blue index but a long lifetime compared with the boron‐based *t*‐DABNA. The NBisICz–PCz device exhibited the highest blue index and longest device lifetime. Notably, the blue index of NBisICz–PCz was comparable with that of *t*‐DABNA, but its device lifetime was higher than that of *t*‐DABNA, indicating the superiority of the NBisICz framework. These results may be attributed to the excellent intermolecular blocking function and stability of the NBisICz framework due to the stable fused aromatic structure.

**Figure 8:**
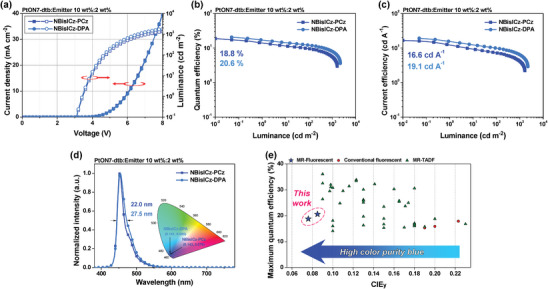
Device performance of 2 wt%‐doped blue‐phosphorescence‐sensitized fluorescence devices. a) Current density–voltage–luminance curves. b) External quantum efficiency (EQE)–luminance curves. c) Current efficiency–luminance curves. d) Normalized electroluminescence spectra (inset: corresponding CIE color coordinates) (blue: NBisICz–PCz; sky blue: NBisICz–DPA), e) EQE_Max_–CIE_y_ data for the blue phosphor‐sensitized fluorescence OLEDs.

PSF devices were fabricated to improve the device efficiency and color purity of the blue OLEDs. **Figure** **8** and Table 3 show the PSF device performance. The terminal emitter with 2 wt% doping exhibited the optimum device performance, which is attributed to complete Förster energy transfer. The EQE_Max_ values for the NBisICz–PCz‐ and NBisICz–DPA‐embedded PSF devices increased by 18.8% and 20.6%, respectively, which is attributed to the triplet exciton harvesting function of PtON7‐dtb. In addition, the devices recorded pure blue emission spectra with λ_EL_ of 453 and 454 nm and FWHM values of 22.0 and 27.5 nm, respectively. The emission wavelength of the devices was slightly hypsochromically shifted relative to that of the TTF devices, and the main peak emission bandwidth was narrowed by ≈3 nm. Thus, the NBisICz–PCz and NBisICz–DPA embedded PSF devices exhibited improved blue color purity with CIE_y_ values of 0.070 and 0.083, resulting in significantly improved blue indexes of 190.0 and 204.8 cd A**^−^** ^1^, respectively.

Figure 8e shows the summary of EQE_Max_–CIE_y_ plot for the PSF devices reported so far, and Table S4 (Supporting Information) lists the detailed device performances. Although numerous PSF devices have been reported, most fluorescent‐emitter‐embedded PSF devices exhibit poor color purity with CIE_y_ ≥ 0.19 because of the poor emission color of the terminal emitters. This issue can be overcome by employing NBisICz–PCz as the terminal emitter. The EQE of the fabricated PSF device was comparable to that of other PSF devices with conventional fluorescent emitters; however, it can be improved by using a phosphor sensitizer, which can completely harvest the singlet excitons of the deep blue‐emitting NBisICz–PCz emitter. PtON7‐dtb is not an ideal phosphorescent sensitizer because of its low PLQY and long emission wavelength. Therefore, the proposed spin‐flip‐restricted ICz structure design strategy is promising for the development of high‐performance blue devices.

## 3 Conclusion

Fused‐ICz‐based blue MR‐type emitters (NBisICz, NBisICz–PCz, and NBisICz–DPA) with spin‐flip‐restricted emission were developed as narrow‐emitting fluorescent emitters by expanding the *π*‐system of the bis‐fused‐ICz framework. They energetically stabilized the T_1_ state and suppressed intertriplet vibronic resonance, thereby deactivating the SVC‐TADF mechanism. Owing to the sp^2^ nitrogen‐based stable aromatic structure and its stabilized *E*_T_, NBisICz showed improved exciton stability compared with *t*‐DABNA. Thus, the NBisICz–PCz‐embedded MR–TTF device exhibited a blue index of 68.6 cd A^−1^ at CIE_y_ of 0.075 and a device lifetime 1.8 times longer than that of *t*‐DABNA. In addition, the PSF devices exhibited a higher blue index of 224.6 cd A^−1^. These results show that the proposed emitter design strategy is an effective approach for the development of advanced blue emitters.

## Conflict of Interest

The authors declare no conflict of interest.

## Supporting information

Supporting Information

## Data Availability

The data that support the findings of this study are available from the corresponding author upon reasonable request.
