# Supplementary material for: Spin‐Flip‐Restricted Multiple‐Resonance Emitters for Extended Device Lifetime in Indolocarbazole‐Based Blue Organic Light‐Emitting Diodes
Source: Adv Sci (Weinh). 2024 Aug 29;11(40):2405604. doi: 10.1002/advs.202405604 (PMC11515912; doi:10.1002/advs.202405604)
Supplement: Supplementary file 1 — Supporting Information [file ADVS-11-2405604-s001.docx]

**Supporting Information**

**Spin-Flip-Restricted Multiple Resonance Emitters for Extending Device Lifetime in Indolocarbazole-Based Blue Organic Light-Emitting Diodes**

Jihoon Kang^1^, Ha Lim Lee^1,2^, Soon Ok Jeon^2^, Hye Jin Bae^2^, Seung Chan Kim^1^, Seungwon Han^3^, and Jun Yeob Lee^1,4‡^

^1^School of Chemical Engineering, Sungkyunkwan University

2066, Seobu-ro, Jangan-gu, Suwon, Gyeonggi, 16419, Republic of Korea

E-mail: leej17@skku.edu

^2^Samsung Advanced Institute of Technology, Samsung Electronics Co., Ltd.,

130 Samsung-ro, Yeongtong-gu, Suwon, Gyeonggi, 16678, Republic of Korea

^3^Department of Display Convergence Engineering, Sungkyunkwan University

2066, Seobu-ro, Jangan-gu, Suwon, Gyeonggi, 16419, Republic of Korea

^4^SKKU Institute of Energy Science and Technology, Sungkyunkwan University

2066, Seobu-ro, Jangan-gu, Suwon, Gyeonggi, 16419, Republic of Korea

* To whom correspondence should be addressed

**Supporting Information**

**1. Synthesis**

***1.1. General information***

***1.2. Synthetic procedure***

**2. Quantum chemical calculation**

**3. Material characterization**

**4. Device characterization**

**5. Supplementary figures**

**6. Supplementary tables**

**Supplementary references**

**1. Synthesis**

***1.1. General information***

All reagents, catalysts, bases, and solvents used in this paper are commercially available. The starting materials, naphthalen-2-amine, 1,3-dibromo-2-fluorobenzene, 3,6-dibromo-9*H*-carbazole, and (3,5-di-*tert*-butylphenyl)boronic acid were purchased from companies. 3,6-bis(3,5-di-*tert*-butylphenyl)-9*H*-carbazole was synthesized according to the previously reported method in the literature.^[S1]^

^1^H NMR spectra were recorded at 500 MHz and ^13^C NMR spectra were recorded on a 125 MHz instrument using DMSO-*d*_6_, CDCl_3_, and CD_2_Cl_2_ solvents. Low resolution mass spectroscopy used a mass spectrometer (Advion, Expression) using atmospheric pressure chemical ionization (APCI) source. High resoltuion mass spectroscopy of the final compound was performed using JMS-700(JEOL) instrument under high resolution fast atom bombardment (HR-FAB) mode.

***1.2. Synthetic procedure***

Scheme S1. Synthetic scheme of NBisICz, NBisICz-PCz and NBisICz-DPA.

**3,6-Bis(3,5-di-*tert*-butylphenyl)-9*H*-carbazole**

3,6-Bis(3,5-di-*tert*-butylphenyl)-9*H*-carbazole was synthesized according to the synthetic procedure described in the reported paper.^[S1]^

***N*-(2,6-dibromophenyl)naphthalen-2-amine (1)**

Naphthalen-2-amine (10.39 g, 72.56 mmol), 1,3-dibromo-2-fluorobenzene (22.10 g, 87.04 mmol), potassium *tert*-butoxide (KOtBu, 20.36 g, 181.41 mmol) and *N*,*N*-dimethylformamide (60 mL) were mixed in 250 mL seal tube. The reaction phase was stirred and heated with 80 °C for 8 h. After end of reaction, the reaction phase was cooled down to room temperature. It was mixed and stirred with 250 mL of deionized water (DW) for 30 min. The reaction phase was extracted with methylene chloride (MC) and DW. The extracted organic phase was dried with anhydrous magnessium sulfate (MgSO_4_) to remove residual moisture and filtered under reduced pressure with MC. The filtrate was concentrated with rotary evaporator under reduced pressure. The concentrated filtrate was adsorbed in silica gel and purified through column chromatography with an eluent of MC:*n*-hexane (Hex) (1:4) (17.69g, 65%). The product was obtained as a stiky liquid phase.

^1^H NMR (500 MHz, CDCl_3_): δ 7.74 (d, *J* = 3.1 Hz, 1H), 7.72 (d, *J* = 2.4 Hz, 1H), 7.65 (s, 1H), 7.63 (s, 1H), 7.59 (d, *J* = 8.7 Hz, 1H), 7.37 (ddd, *J* = 8.2, 6.8, 1.3 Hz, 1H), 7.27 (ddd, *J* = 8.1, 6.8, 1.2 Hz, 1H), 7.04 (dd, *J* = 8.8, 2.4 Hz, 1H), 6.97 (t, *J* = 8.0 Hz, 1H), 6.84 (d, *J* = 2.3 Hz, 1H).

MS (APCI) m/z: Found 375.88 [(M + H)^+^]. Calculated for C_16_H_11_Br_2_N: 374.93.

**8-Bromo-7*H*-benzo[*c*]carbazole (2)**

*N*-(2,6-Dibromophenyl)naphthalen-2-amine (17.00 g, 45.08 mmol), tris(dibenzylideneacetone)dipalladium(0) (Pd_2_(dba)_3_) (0.44 g, 0.48 mmol), tri-*tert*-butylphosphine (P(tBu)_3_) (50 wt% in toluene, 4.7 mL, 14.07 mmol), potassium phosphate tribasic (K_3_PO_4_) (29.87 g, 140.71 mmol) and 140 mL of dimethylacetamide (DMAc) were mixed in 2-neck 250 mL round-bottom flask. The reaction phase was stirred and refluxed for 32 h. After end of reaction, the reaction phase was cooled down to room temperature and diluted with 100 mL of MC. After filtration with celite/silica gel packed short filter under reduced pressure, the filtrate was concentrated using rotary evaporator. The crude product was adsorbed in silica gel and purified through column chromatography with an eluent of MC:Hex (1:4) (1.61 g, 12%). The product was obtained as a white solid.

^1^H NMR (500 MHz, DMSO-*d*_6_): δ 11.95 (s, 1H), 8.77 (d, *J* = 8.3 Hz, 1H), 8.62 (d, *J* = 7.9 Hz, 1H), 8.09 (d, *J* = 8.0 Hz, 1H), 7.98 (d, *J* = 8.8 Hz, 1H), 7.85 (d, *J* = 8.8 Hz, 1H), 7.73 (t, *J* = 7.6 Hz, 1H), 7.67 (d, *J* = 7.6 Hz, 1H), 7.51 (t, *J* = 7.5 Hz, 1H), 7.28 (t, *J* = 7.8 Hz, 1H).

MS (APCI) m/z: Found 296.12 [(M + H)^+^]. Calculated for C_16_H_10_BrN: 295.00.

**8-(4,4,5,5-Tetramethyl-1,3,2-dioxaborolan-2-yl)-7*H*-benzo[*c*]carbazole (3)**

8-Bromo-7*H*-benzo[*c*]carbazole (1.60 g, 5.40 mmol), 4,4,4',4',5,5,5',5'-octamethyl-2,2'-bi(1,3,2-dioxaborolane) (B_2_Pin_2_) (2.74 g, 10.81 mmol), [1,1′-bis(diphenylphosphino)ferrocene]dichloropalladium(II) (PdCl_2_(dppf)) (0.40 g, 0.54 mmol), potassium acetate (KOAc) (1.60 g, 16.21 mmol) and 25 mL of 1,4-dioxane were mixed in 2-neck 100 mL round-bottom flask. The reaction phase was stirred and refluxed for 8 h. After end of reaction, the reaction phase was cooled down to room temperature and diluted with 50 mL of MC. After filtration with celite/silica gel packed short filter under reduced pressure, the filtrate was concentrated using rotary evaporator. The crude product was adsorbed in silica gel and purified through column chromatography with an eluent of MC:Hex (1:2) (1.31 g, 71%). The product was obtained as a yellowish white solid.

^1^H NMR (500 MHz, CDCl_3_): δ 9.56 (s, 1H), 8.77 (d, *J* = 8.3 Hz, 1H), 8.68 (d, *J* = 7.9 Hz, 1H), 8.00 (d, *J* = 8.0 Hz, 1H), 7.90 (dd, *J* = 7.1, 1.1 Hz, 1H), 7.87 (d, *J* = 8.8 Hz, 1H), 7.72 (d, *J* = 8.8 Hz, 1H), 7.71 – 7.67 (m, 1H), 7.47 (ddd, *J* = 8.1, 6.9, 1.1 Hz, 1H), 7.39 (dd, *J* = 7.9, 7.2 Hz, 1H), 1.46 (s, 12H).

MS (APCI) m/z: Found 344.26 [(M + H)^+^]. Calculated for C_22_H_22_BNO_2_: 343.17.

**1-Bromo-3,6-bis(3,5-di-*tert*-butylphenyl)-9*H*-carbazole (4)**

3,6-Bis(3,5-di-*tert*-butylphenyl)-9*H*-carbazole (8.16g, 15.00 mmol) was dissolved in 100 mL of tetrahydrofuran (THF). It was added into 2-neck 250 mL round-bottom flask and cooled down to 0 °C using icebath. The *N*-bromosuccinimide (NBS) (2.93 g, 16.46 mmol) was dissolved in 20 mL of DMF and NBS solution was slowly added to round-bottom flask using dropping funnel. At this time, the reactor was wrapped with Al foil to block the external light. After 8 h, 2 M aqueous sodium thiosulfate solution was added to reaction phase to quench the reaction phase and stirred for 30 min. The crude product was extracted with MC and DW. The extracted organic phase was dried with anhydrous MgSO_4_ and filtered under reduced pressure with MC. The filtrate was concentrated with rotary evaporator under reduced pressure. The concentrated filtrate was adsorbed in silica gel and purified through column chromatography with an eluent of MC:Hex (1:6) (8.30 g, 89%). The product was obtained as a white solid.

^1^H NMR (500 MHz, CDCl_3_): δ 8.27 (d, *J* = 1.5 Hz, 1H), 8.22 (d, *J* = 1.2 Hz, 1H)z, 7.80 (d, *J* = 1.4 Hz, 1H), 7.71 (dd, *J* = 8.4, 1.7 Hz, 1H), 7.57 (d, *J* = 8.4 Hz, 1H), 7.51 (d, *J* = 1.8 Hz, 2H), 7.47 (d, *J* = 1.8 Hz, 2H), 7.45 (dd, *J* = 3.3, 1.6 Hz, 2H), 1.41 (s, 36H).

MS (APCI) m/z: Found 622.31 [(M + H)^+^]. Calculated for C_40_H_48_BrN: 621.30.

**3,6-Bis(3,5-di-*tert*-butylphenyl)-1-(4,4,5,5-tetramethyl-1,3,2-dioxaborolan-2-yl)-9*H*-carbazole (5)**

The same synthetic method of 8-(4,4,5,5-tetramethyl-1,3,2-dioxaborolan-2-yl)-7*H*-benzo[*c*]carbazole (3) was applied to synthesize 3,6-bis(3,5-di-*tert*-butylphenyl)-1-(4,4,5,5-tetramethyl-1,3,2-dioxaborolan-2-yl)-9*H*-carbazole (5). The reaction phase was composed of 1-Bromo-3,6-bis(3,5-di-*tert*-butylphenyl)-9*H*-carbazole (8.00 g, 12.85 mmol), B_2_Pin_2_ (4.89 g, 19.27 mmol), PdCl_2_(dppf) (0.28 g, 0.39 mmol), KOAc (2.52 g, 25.67 mmol) and 80 mL of 1,4-dioxane. The reaction time was 8 h and the eluent of MC:Hex (1:1) was used for column chromatography (6.19 g, 72%). The product was obtained as a white solid.

^1^H NMR (500 MHz, CDCl_3_): δ 9.18 (s, 1H), 8.39 (d, *J* = 1.7 Hz, 1H), 8.30 (d, *J* = 1.6 Hz, 1H), 8.05 (d, *J* = 1.8 Hz, 1H), 7.68 (dd, *J* = 8.4, 1.7 Hz, 1H), 7.57 (d, *J* = 8.4 Hz, 1H), 7.53 (d, *J* = 1.8 Hz, 2H), 7.49 (d, *J* = 1.8 Hz, 2H), 7.42 (dt, *J* = 3.7, 1.8 Hz, 2H), 1.46 (s, 12H), 1.41 (d, *J* = 1.7 Hz, 36H).

MS (APCI) m/z: Found 670.49 [(M + H)^+^]. Calculated for C_46_H_60_BNO_2_: 669.47.

**3,6-Bis(3,5-di-*tert*-butylphenyl)-1-(2,5-dibromo-4-iodophenyl)-9*H*-carbazole (6)**

3,6-Bis(3,5-di-*tert*-butylphenyl)-1-(4,4,5,5-tetramethyl-1,3,2-dioxaborolan-2-yl)-9*H*-carbazole (7.10 g, 7.47 mmol), 1,4-dibromo-2,5-diiodobenzene (5.46 g, 11.20 mmol), tetrakis(triphenylphosphine)palladium (0) (Pd(PPh_3_)_4_) (0.26 g, 0.22 mmol), dicyclohexyl(2′,6′-dimethoxy[1,1′-biphenyl]-2-yl)phosphane (SPhos) (0.18 g, 0.44 mmol), 25 mL of 4 M aqueous K_3_PO_4_ solution and 50 mL of 1,2-dimethoxyethane (DME) were mixed in 2-neck 250 mL round-bottom flask. The mixture was stirred and refluxed for 18 h. After end of reaction, the cooled reaction phase was extracted with MC and DW. The extracted organic phase was dried with anhydrous MgSO_4_ and filtered under reduced pressure with MC. The filtrate was concentrated with rotary evaporator under reduced pressure. The concentrated filtrate was adsorbed in silica gel and purified through column chromatography with an eluent of MC:Hex (1:4) (1.31 g, 19%). The product was obtained as a white solid.

^1^H NMR (500 MHz, CDCl_3_): δ 8.35 (d, *J* = 1.6 Hz, 1H), 8.32 (d, *J* = 1.5 Hz, 1H), 8.28 (s, 1H), 7.89 (s, 1H), 7.82 (s, 1H), 7.68 (dd, *J* = 8.4, 1.7 Hz, 1H), 7.53 – 7.51 (m, 5H), 7.50 – 7.47 (dd, *J* = 8.3, 0.5 Hz, 1H), 7.44 (dd, *J* = 3.1, 1.7 Hz, 1H), 1.41 (d, *J* = 1.6 Hz, 36H).

MS (APCI) m/z: Found 902.18 [(M + H)^+^]. Calculated for C_46_H_50_Br_2_IN: 901.14.

**8-(4-(3,6-Bis(3,5-di-*tert*-butylphenyl)-9*H*-carbazol-1-yl)-2,5-dibromophenyl)-7*H*-benzo[*c*]carbazole (7)**

The same synthetic method of 3,6-bis(3,5-di-*tert*-butylphenyl)-1-(2,5-dibromo-4-iodophenyl)-9*H*-carbazole (6) was applied to synthesize 8-(4-(3,6-bis(3,5-di-*tert*-butylphenyl)-9*H*-carbazol-1-yl)-2,5-dibromophenyl)-7*H*-benzo[*c*]carbazole (7). The reaction phase was composed of 3,6-bis(3,5-di-*tert*-butylphenyl)-1-(2,5-dibromo-4-iodophenyl)-9*H*-carbazole (0.70 g, 0.77 mmol), 8-(4,4,5,5-tetramethyl-1,3,2-dioxaborolan-2-yl)-7*H*-benzo[*c*]carbazole (0.22 g, 0.65 mmol), Pd(PPh_3_)_4_ (0.03 g, 0.02 mmol), SPhos (0.02 g, 0.05 mmol), 5 mL of 2 M aqueous K_3_PO_4_ solution and 10 mL of DME. The reaction time was 4 h and the eluent of MC:Hex (1:4) was used for column chromatography (0.47 g, 73%). The product was obtained as a white solid.

^1^H NMR (300 MHz, DMSO-d_6_) : δ 8.34-8.20 (m, 6H), 7.64-7.60 (dd, *J*_1_=8.70 Hz, *J*_2_=2.10 Hz, 1H), 7.44-7.43 (d, *J*=2.40 Hz, 1H), 7.13-7.12 (d, *J*=2.10 Hz, 1H), 4.66 (s, 2H), 1.50 (s, 9H), 1.43 (s, 9H), 1.25 (s, 9H)

MS (APCI) m/z: Found 991.50 [(M + H)^+^]. Calculated for C_62_H_60_Br_2_N_2_: 990.31.

**10,13-Bis(3,5-di-*tert*-butylphenyl)-indolo[3,2,1-*jk*]benzo[4',5']indolo[3',2',1':7,1]indolo[3,2-*b*]carbazole (NBisICz)**

8-(4-(3,6-Bis(3,5-di-*tert*-butylphenyl)-9*H*-carbazol-1-yl)-2,5-dibromophenyl)-7*H*-benzo[*c*]carbazole (0.400 g, 0.403 mmol), CuI (0.077 g, 0.403 mmol), 1,10-phenanthroline (0.073 g, 0.403 mmol), K_3_PO_4_ (0.342 g, 1.611 mmol) and 5 mL of DMF were mixed in 40 mL vial. The mixture was stirred and heated with 100 °C for 8 h. After end of reaction, the cooled reaction phase was quenched with 30 mL of DW. After stirring for 30 min, the precipitated reaction phase was filtered with DW and dried in vacuum oven. The dried crude product was adsorbed in silica gel and purified through column chromatography with an eluent of MC:Hex (1:4). To further purification, the obtained pure product was reprecipitated with MC and MeOH. The product was obtained as a yellow powder by filtering and drying in vacuum oven (0.275 g, 82%). To apply the vacuum deposition process, a train sublimation method was carried out at 10^-3^ torr to purify final product.

^1^H NMR (500 MHz, CDCl_3_): δ 8.34 (d, *J* = 8.1 Hz, 1H), 8.26 (s, 1H), 8.24 – 8.21 (m, 3H), 8.19 (d, *J* = 0.8 Hz, 1H), 8.10 (s, 1H), 8.07 (d, *J* = 7.2 Hz, 1H), 7.96 (d, *J* = 8.7 Hz, 1H), 7.87 (m, 2H), 7.79 (dd, *J* = 8.3, 1.6 Hz, 2H), 7.70 (d, *J* = 1.8 Hz, 2H), 7.66 (t, *J* = 7.3 Hz, 1H), 7.61 (d, *J* = 1.8 Hz, 2H), 7.55 (t, *J* = 1.8 Hz, 1H), 7.53 – 7.48 (m, 2H), 7.40 (ddd, *J* = 7.9, 6.8, 1.1 Hz, 1H), 1.53 (s, 18H), 1.48 (s, 18H).

MS (HR-FAB) m/z: Found 830.4596 [(M)^+^]. Calculated for C_62_H_58_N_2_: 830.4600.

**1,8-Dibromo-3,6-bis(3,5-di-*tert*-butylphenyl)-9*H*-carbazole (8)**

The same synthetic method of 1-bromo-3,6-bis(3,5-di-*tert*-butylphenyl)-9*H*-carbazole (4) was applied to synthesize 1,8-dibromo-3,6-bis(3,5-di-*tert*-butylphenyl)-9*H*-carbazole (8). The reaction phase was composed of 3,6-bis(3,5-di-*tert*-butylphenyl)-9*H*-carbazole (7.70 g, 14.16 mmol), NBS (5.29 g, 29.43 mmol) and 90 mL of THF, 30 mL of DMF were used to dissolve each compound, respectively. The reaction time was 8 h and the eluent of MC:Hex (1:6) was used for column chromatography (8.73 g, 88%). The product was obtained as a white solid.

^1^H NMR (500 MHz, CDCl_3_): δ 8.35 (s, 1H), 8.19 (d, *J* = 1.3 Hz, 2H), 7.84 (d, *J* = 1.4 Hz, 2H), 7.46 (s, 6H), 1.41 (s, 36H).

MS (APCI) m/z: Found 700.22 [(M + H)^+^]. Calculated for C_40_H_47_Br_2_N: 699.21.

**8-Bromo-3,6-bis(3,5-di-*tert*-butylphenyl)-9'-phenyl-9*H*,9'*H*-1,3'-bicarbazole (9)**

The same synthetic method of 3,6-bis(3,5-di-*tert*-butylphenyl)-1-(2,5-dibromo-4-iodophenyl)-9*H*-carbazole (6) was applied to synthesize 8-Bromo-3,6-bis(3,5-di-*tert*-butylphenyl)-9'-phenyl-9*H*,9'*H*-1,3'-bicarbazole (9). The reaction phase was composed of 1,8-Dibromo-3,6-bis(3,5-di-*tert*-butylphenyl)-9*H*-carbazole (11.90 g, 16.96 mmol), (9-phenyl-9*H*-carbazol-3-yl)boronic acid (3.25 g, 11.31 mmol), Pd(PPh_3_)_4_ (0.39 g, 0.34 mmol), 60 mL of 2 M aqueous potassium carbonate (K_2_CO_3_) solution and 120 mL of THF. The reaction time was 4 h and the eluent of MC:Hex (1:6) was used for column chromatography (3.69 g, 38%). The product was obtained as a white solid.

^1^H NMR (500 MHz, CDCl_3_): δ 8.52 (d, *J* = 1.3 Hz, 1H), 8.49 (s, 1H), 8.28 (dd, *J* = 4.9, 1.4 Hz, 2H), 8.23 (dt, *J* = 7.0, 0.8 Hz, 1H), 7.82 (dd, *J* = 9.8, 1.5 Hz, 3H), 7.66 (d, *J* = 1.9 Hz, 2H), 7.65 (s, 3H), 7.58 (d, *J* = 1.8 Hz, 2H), 7.50 (d, *J* = 1.8 Hz, 2H), 7.48 (d, *J* = 0.8 Hz, 1H), 7.47 – 7.45 (m, 3H), 7.34 (ddd, *J* = 8.0, 4.5, 3.6 Hz, 1H), 1.42 (s, 18H), 1.42 (s, 18H).

MS (APCI) m/z: Found 863.43 [(M + H)^+^]. Calculated for C_58_H_59_BrN_2_: 862.39.

**3,6-Bis(3,5-di-*tert*-butylphenyl)-9'-phenyl-8-(4,4,5,5-tetramethyl-1,3,2-dioxaborolan-2-yl)-9*H*,9'*H*-1,3'-bicarbazole (10)**

The same synthetic method of 8-(4,4,5,5-tetramethyl-1,3,2-dioxaborolan-2-yl)-7*H*-benzo[*c*]carbazole (3) was applied to synthesize 3,6-bis(3,5-di-*tert*-butylphenyl)-9'-phenyl-8-(4,4,5,5-tetramethyl-1,3,2-dioxaborolan-2-yl)-9*H*,9'*H*-1,3'-bicarbazole (10). The reaction phase was composed of 8-Bromo-3,6-bis(3,5-di-*tert*-butylphenyl)-9'-phenyl-9*H*,9'*H*-1,3'-bicarbazole (4.94 g, 5.72 mmol), B_2_Pin_2_ (2.18 g, 8.58 mmol), PdCl_2_(dppf) (0.13 g, 0.17 mmol), KOAc (1.12 g, 11.44 mmol) and 50 mL of 1,4-dioxane. The reaction time was 8 h and the eluent of MC:Hex (1:1) was used for column chromatography. To further purification, the product was reprecipitated using MC and DW (3.24 g, 62%). The product was obtained as a white solid.

^1^H NMR (500 MHz, CDCl_3_): δ 9.82 (s, 1H), 8.65 (d, *J* = 1.5 Hz, 1H), 8.46 (d, *J* = 1.7 Hz, 1H), 8.31 (d, *J* = 1.4 Hz, 1H), 8.23 (d, *J* = 7.8 Hz, 1H), 8.07 (d, *J* = 1.8 Hz, 1H), 7.89 (dd, *J* = 8.5, 1.7 Hz, 1H), 7.85 (d, *J* = 1.6 Hz, 1H), 7.66 (s, 2H), 7.66 (s, 2H), 7.63 (d, *J* = 8.3 Hz, 1H), 7.61 (d, *J* = 1.8 Hz, 2H), 7.53 (d, *J* = 1.8 Hz, 2H), 7.53 – 7.51 (m, 1H), 7.46 (dt, *J* = 3.4, 1.3 Hz, 3H), 7.43 (t, *J* = 1.8 Hz, 1H), 7.32 (ddd, *J* = 7.9, 6.3, 1.7 Hz, 1H), 1.43 (s, 18H), 1.43 (s, 18H), 1.34 (s, 12H).

MS (APCI) m/z: Found 911.71 [(M + H)^+^]. Calculated for C_64_H_71_BN_2_O_2_: 910.56.

**3,6-Bis(3,5-di-*tert*-butylphenyl)-8-(2,5-dibromo-4-iodophenyl)-9'-phenyl-9*H*,9'*H*-1,3'-bicarbazole (11)**

The same synthetic method of 3,6-bis(3,5-di-*tert*-butylphenyl)-1-(2,5-dibromo-4-iodophenyl)-9*H*-carbazole (6) was applied to synthesize 3,6-bis(3,5-di-*tert*-butylphenyl)-8-(2,5-dibromo-4-iodophenyl)-9'-phenyl-9*H*,9'*H*-1,3'-bicarbazole (11). The reaction phase was composed of 3,6-bis(3,5-di-*tert*-butylphenyl)-9'-phenyl-8-(4,4,5,5-tetramethyl-1,3,2-dioxaborolan-2-yl)-9*H*,9'*H*-1,3'-bicarbazole (1.78 g, 3.66 mmol), 1,4-dibromo-2,5-diiodobenzene (5.00 g, 5.49 mmol), Pd(PPh_3_)_4_ (0.42 g, 0.37 mmol), SPhos (0.30 g, 0.73 mmol), 25 mL of 4 M aqueous K_3_PO_4_ solution and 50 mL of 1,4-dioxane. The reaction time was 10 h and the eluent of MC:Hex (1:4) was used for column chromatography (0.97 g, 23%). The product was obtained as a white solid.

^1^H NMR (500 MHz, ) δ 8.53 (d, *J* = 1.4 Hz, 1H), 8.46 (d, *J* = 1.4 Hz, 1H), 8.36 (d, *J* = 1.4 Hz, 1H), 8.29 (s, 1H), 8.24 – 8.20 (m, 2H), 7.82 (s, 1H), 7.81 (d, *J* = 1.7 Hz, 1H), 7.79 (dd, *J* = 8.4, 1.8 Hz, 1H), 7.68 – 7.61 (m, 4H), 7.60 (d, *J* = 1.8 Hz, 2H), 7.59 – 7.56 (m, 3H), 7.56 (d, *J* = 1.7 Hz, 1H), 7.54 – 7.49 (m, 1H), 7.47 – 7.44 (m, 4H), 1.41 (s, 18H), 1.40 (s, 18H).

MS (APCI) m/z: Found 1143.41 [(M + H)^+^]. Calculated for C_64_H_61_Br_2_IN_2_: 1142.22.

**8-(4-(3,6-Bis(3,5-di-*tert*-butylphenyl)-9'-phenyl-9*H*,9'*H*-[1,3'-bicarbazol]-8-yl)-2,5-dibromophenyl)-7*H*-benzo[*c*]carbazole (12)**

The almost same synthetic method of 3,6-bis(3,5-di-*tert*-butylphenyl)-1-(2,5-dibromo-4-iodophenyl)-9*H*-carbazole (6) was applied to synthesize 8-(4-(3,6-bis(3,5-di-*tert*-butylphenyl)-9'-phenyl-9*H*,9'*H*-[1,3'-bicarbazol]-8-yl)-2,5-dibromophenyl)-7*H*-benzo[*c*]carbazole (12). 3,6-Bis(3,5-di-*tert*-butylphenyl)-8-(2,5-dibromo-4-iodophenyl)-9'-phenyl-9*H*,9'*H*-1,3'-bicarbazole (0.500 g, 0.437 mmol), 8-(4,4,5,5-tetramethyl-1,3,2-dioxaborolan-2-yl)-7*H*-benzo[*c*]carbazole (0.125 g, 0.364 mmol), Pd(PPh_3_)_4_ (0.021 g, 0.018 mmol), SPhos (0.040 g, 0.097 mmol), 25 mL of 4 M aqueous K_3_PO_4_ solution and 50 mL of 1,4-dioxane were mixed into 40 mL vial. The reaction phase was heated up to 100 °C for 2 h and the eluent of MC:Hex (1:4) was used for column chromatography (0.353 g, 65%). The product was obtained as a white solid.

MS (APCI) m/z: Found 1232.59 [(M + H)^+^]. Calculated for C_80_H_71_Br_2_N_3_: 1231.40.

**10,13-Bis(3,5-di-*tert*-butylphenyl)-15-(9-phenyl-carbazol-3yl)-indolo[3,2,1-*jk*]benzo[4',5']indolo[3',2',1':7,1]indolo[3,2-*b*]carbazole (NBisICz-PCz)**

The same synthetic method of NBisICz was applied to synthesize NBisICz-PCz. The reaction phase was composed of 8-(4-(3,6-Bis(3,5-di-*tert*-butylphenyl)-9'-phenyl-9*H*,9'*H*-[1,3'-bicarbazol]-8-yl)-2,5-dibromophenyl)-7*H*-benzo[*c*]carbazole (0.316 g, 0.256 mmol), CuI (0.048 g, 0.256 mmol), 1,10-phenanthroline (0.046 g, 0.256 mmol), K_3_PO_4_ (0.217 g, 1.024 mmol) and DMF (5 mL). The eluent of MC:Hex (1:4) was used for column chromatography (0.256 g, 93%).

^1^H NMR (500 MHz, CDCl_3_): δ 8.64 (s, 1H), 8.45 (d, *J* = 1.8 Hz, 1H), 8.38 (s, 1H), 8.29 – 8.25 (m, 3H), 8.12 (s, 1H), 8.02 (d, *J* = 7.5 Hz, 1H), 7.92 (s, 1H), 7.89 (s, 1H), 7.86 – 7.79 (m, 4H), 7.74 (d, *J* = 1.4 Hz, 2H), 7.72 – 7.69 (m, 5H), 7.68 – 7.64 (m, 2H), 7.58 – 7.48 (m, 4H), 7.43 (s, 1H), 7.37 – 7.34 (m, 1H), 7.27 (s, 1H), 7.13 (s, 1H), 6.61 (d, *J* = 7.2 Hz, 1H), 1.54 (s, 18H), 1.48 (s, 18H).

^13^C NMR (126 MHz, CDCl_3_): δ 151.40, 151.31, 141.77, 140.83, 137.70, 130.21, 129.13, 128.74, 127.84, 127.22, 126.86, 123.79, 123.49, 122.98, 122.69, 122.48, 122.06, 121.36, 120.97, 120.92, 120.73, 119.22, 119.06, 118.89, 117.30, 110.22, 109.85, 35.24, 35.20, 31.85, 31.77.

MS (HR-FAB) m/z: Found 1072.5576 [(M + H)^+^]. Calculated for C_80_H_69_N_3_: 1071.5491

**8-Bromo-3,6-bis(3,5-di-*tert*-butylphenyl)-*N*,*N*-diphenyl-9*H*-carbazol-1-amine (13)**

1,8-Dibromo-3,6-bis(3,5-di-*tert*-butylphenyl)-9*H*-carbazole (2.25 g, 3.21 mmol), diphenylamine (0.27 g, 1.60 mmol), Pd_2_(dba)_3_ (0.07 g, 0.08 mmol), dicyclohexyl[2′,4′,6′-tris(propan-2-yl)[1,1′-biphenyl]-2-yl]phosphane (XPhos) (0.08 g, 0.16 mmol), sodium *tert*-butoxide (NaOtBu) (0.31 g, 3.2 mmol) and 20 mL of *o*-xylene were mixed in 2-neck 100 mL round-bottom flask. The reaction mixture was stirred and refluxed for 4 h. After end of reaction, the reaction phase was cooled down to room temperature and diluted with 50 mL of MC. After filtration with celite/silica gel packed short filter under reduced pressure, the filtrate was concentrated using rotary evaporator. The crude product was adsorbed in silica gel and purified through column chromatography with an eluent of MC:Hex (1:6) (0.59 g, 47%). The product was obtained as a white solid.

^1^H NMR (500 MHz, CDCl_3_): δ 8.18 (d, *J* = 1.1 Hz, 1H), 8.05 (s, 1H), 7.72 (d, *J* = 1.4 Hz, 1H), 7.54 (s, 1H), 7.47 (s, 1H), 7.44 (s, 3H), 7.40 (s, 3H), 7.30 (t, *J* = 7.9 Hz, 4H), 7.20 (d, *J* = 7.8 Hz, 4H), 7.06 (t, *J* = 7.3 Hz, 2H), 1.40 (s, 18H), 1.37 (s, 18H).

MS (APCI) m/z: Found 789.42 [(M + H)^+^]. Calculated for C_52_H_57_BrN_2_: 788.37.

**3,6-Bis(3,5-di-*tert*-butylphenyl)-*N*,*N*-diphenyl-8-(4,4,5,5-tetramethyl-1,3,2-dioxaborolan-2-yl)-9*H*-carbazol-1-amine (14)**

The same synthetic method of 8-(4,4,5,5-tetramethyl-1,3,2-dioxaborolan-2-yl)-7*H*-benzo[*c*]carbazole (3) was applied to synthesize 3,6-Bis(3,5-di-*tert*-butylphenyl)-*N*,*N*-diphenyl-8-(4,4,5,5-tetramethyl-1,3,2-dioxaborolan-2-yl)-9*H*-carbazol-1-amine (14). The reaction phase was composed of 8-bromo-3,6-bis(3,5-di-*tert*-butylphenyl)-*N*,*N*-diphenyl-9*H*-carbazol-1-amine (2.60 g, 3.29 mmol), B_2_Pin_2_ (1.67 g, 6.58 mmol), PdCl_2_(dppf) (0.24 g, 0.33 mmol), KOAc (0.97 g, 9.87 mmol) and 25 mL of 1,4-dioxane. The reaction time was 8 h and the eluent of MC:Hex (1:1) was used for column chromatography (1.81 g, 66%). The product was obtained as a white solid.

^1^H NMR (500 MHz, CDCl_3_): δ 8.73 (s, 1H), 8.38 (d, *J* = 1.7 Hz, 1H), 8.12 (d, *J* = 1.2 Hz, 1H), 7.99 (d, *J* = 1.9 Hz, 1H), 7.48 (d, *J* = 1.8 Hz, 2H), 7.43 (dd, *J* = 4.9, 1.7 Hz, 4H), 7.40 (t, *J* = 1.8 Hz, 1H), 7.31 – 7.26 (m, 4H), 7.21 (dd, *J* = 8.7, 1.1 Hz, 4H), 7.05 – 7.01 (m, 2H), 1.42 (s, 18H), 1.38 (s, 18H), 1.27 (s, 12H).

MS (APCI) m/z: Found 837.50 [(M + H)^+^]. Calculated for C_58_H_69_BN_2_O_2_: 836.55.

**3,6-Bis(3,5-di-*tert*-butylphenyl)-8-(2,5-dibromo-4-iodophenyl)-*N*,*N*-diphenyl-9*H*-carbazol-1-amine (15)**

The same synthetic method of 8-(4-(3,6-bis(3,5-di-*tert*-butylphenyl)-9'-phenyl-9*H*,9'*H*-[1,3'-bicarbazol]-8-yl)-2,5-dibromophenyl)-7*H*-benzo[*c*]carbazole (12) was applied to synthesize 3,6-bis(3,5-di-*tert*-butylphenyl)-8-(2,5-dibromo-4-iodophenyl)-*N*,*N*-diphenyl-9*H*-carbazol-1-amine (15). The reaction phase was composed of 3,6-bis(3,5-di-*tert*-butylphenyl)-*N*,*N*-diphenyl-8-(4,4,5,5-tetramethyl-1,3,2-dioxaborolan-2-yl)-9*H*-carbazol-1-amine (1.00 g, 1.19 mmol), 1,4-dibromo-2,5-diiodobenzene (0.87 g, 1.79 mmol), Pd(PPh_3_)_4_ (0.14 g, 0.12 mmol), SPhos (0.10 g, 0.24 mmol), 7.5 mL of 4 M aqueous K_3_PO_4_ solution and 15 mL of 1,4-dioxane. The reaction time was 4 h and the eluent of MC:Hex (1:4) was used for column chromatography (0.313 g, 71%). The product was obtained as a white solid.

^1^H NMR (500 MHz, CDCl_3_): δ 8.31 (d, *J* = 1.4 Hz, 1H), 8.12 (s, 1H), 8.10 (s, 1H), 7.53 (s, 1H), 7.49 (d, *J* = 1.8 Hz, 2H), 7.46 (d, *J* = 1.5 Hz, 2H), 7.44 (t, *J* = 1.7 Hz, 1H), 7.41 (dd, *J* = 4.1, 1.6 Hz, 3H), 7.25 – 7.21 (m, 5H), 7.13 (s, 3H), 7.12 (s, 1H), 7.01 (t, *J* = 7.3 Hz, 2H), 1.40 (s, 18H), 1.37 (s, 18H).

MS (APCI) m/z: Found 1069.35 [(M + H)^+^]. Calculated for C_58_H_59_Br_2_IN_2_: 1068.21.

**8-(4-(7*H*-benzo[*c*]carbazol-8-yl)-2,5-dibromophenyl)-3,6-bis(3,5-di-*tert*-butylphenyl)-*N*,*N*-diphenyl-9*H*-carbazol-1-amine (16)**

The same synthetic method of 8-(4-(3,6-bis(3,5-di-*tert*-butylphenyl)-9'-phenyl-9*H*,9'*H*-[1,3'-bicarbazol]-8-yl)-2,5-dibromophenyl)-7*H*-benzo[*c*]carbazole (12) was applied to synthesize 8-(4-(7*H*-benzo[*c*]carbazol-8-yl)-2,5-dibromophenyl)-3,6-bis(3,5-di-*tert*-butylphenyl)-*N*,*N*-diphenyl-9*H*-carbazol-1-amine (16). The reaction phase was composed 3,6-bis(3,5-di-*tert*-butylphenyl)-8-(2,5-dibromo-4-iodophenyl)-*N*,*N*-diphenyl-9*H*-carbazol-1-amine (15) (0.486 g, 0.455 mmol), 8-(4,4,5,5-tetramethyl-1,3,2-dioxaborolan-2-yl)-7*H*-benzo[*c*]carbazole (0.130 g, 0.379 mmol), Pd(PPh_3_)_4_ (0.044 g, 0.038 mmol), SPhos (0.031 g, 0.076 mmol), 2.5 mL of 2 M aqueous K_3_PO_4_ solution and 5 mL of 1,4-dioxane. The reaction time was 1.5 h and the eluent of MC:Hex (1:4) was used for column chromatography (0.313 g, 71%). The product was obtained as a white solid.

^1^H NMR (500 MHz, CDCl_3_): δ 8.83 (d, *J* = 8.2 Hz, 1H), 8.67 (d, *J* = 8.0 Hz, 1H), 8.38 (s, 1H), 8.32 (s, 1H), 8.17 (s, 1H), 8.02 (d, *J* = 8.0 Hz, 1H), 7.89 (d, *J* = 8.2 Hz, 1H), 7.84 (s, 1H), 7.79 – 7.72 (m, 2H), 7.63 (s, 1H), 7.57 (d, *J* = 1.2 Hz, 2H), 7.53 – 7.49 (m, 2H), 7.47 (s, 2H), 7.43 (d, *J* = 8.1 Hz, 4H), 7.27 (s, 4H), 7.19 (d, *J* = 6.7 Hz, 4H), 7.04 (t, *J* = 6.0 Hz, 2H), 1.44 (s, 18H), 1.39 (s, 18H).

MS (APCI) m/z: Found 1158.82 [(M + H)^+^]. Calculated for C_74_H_69_Br_2_N_3_: 1157.39.

**10,13-Bis(3,5-di-*tert*-butylphenyl)-*N*^15^,*N*^15^-diphenyl-indolo[3,2,1-*jk*]benzo[4',5']indolo[3',2',1':7,1]indolo[3,2-*b*]carbazol-15-amine (NBisICz-DPA)**

The same synthetic method of NBisICz was applied to synthesize NBisICz-DPA. The reaction phase was composed of 8-(4-(7*H*-benzo[*c*]carbazol-8-yl)-2,5-dibromophenyl)-3,6-bis(3,5-di-*tert*-butylphenyl)-*N*,*N*-diphenyl-9*H*-carbazol-1-amine (0.270 g, 0.233 mmol), CuI (0.044 g, 0.233 mmol), 1,10-phenanthroline (0.040 g, 0.233 mmol), K_3_PO_4_ (0.198 g, 0.932 mmol) and DMF (5 mL). The eluent of MC:Hex (1:4) was used for column chromatography (0.196 g, 84%).

^1^H NMR (500 MHz, CDCl_3_): δ 8.87 (d, *J* = 0.6 Hz, 1H), 8.61 (d, *J* = 7.7 Hz, 1H), 8.52 (d, *J* = 0.6 Hz, 1H), 8.37 (dd, *J* = 2.5, 1.0 Hz, 2H), 8.36 (d, *J* = 1.7 Hz, 1H), 8.32 (d, *J* = 7.5 Hz, 1H), 8.20 (d, *J* = 8.7 Hz, 1H), 7.98 (d, *J* = 8.7 Hz, 1H), 7.95 (d, 1H), 7.89 (d, *J* = 7.2 Hz, 1H), 7.69 (d, *J* = 1.8 Hz, 2H), 7.64 – 7.60 (m, 3H), 7.54 (t, *J* = 1.8 Hz, 1H), 7.48 (d, *J* = 1.8 Hz, 2H), 7.45 (t, *J* = 1.8 Hz, 1H), 7.44 (t, *J* = 1.6 Hz, 2H), 7.43 – 7.42 (m, 3H), 7.34 – 7.30 (m, 4H), 6.99 (tt, *J* = 7.5, 1.1 Hz, 2H), 1.51 (s, 18H), 1.40 (s, 18H).

^13^C NMR (126 MHz, CDCl_3_): δ 151.37, 151.34, 148.32, 143.71, 142.75, 140.23, 139.26, 138.48, 137.80, 136.02, 135.56, 135.12, 132.78, 132.03, 130.96, 130.29, 129.75, 129.71, 129.37, 129.07, 127.96, 127.14, 124.14, 123.93, 123.34, 123.17, 122.98, 122.61, 121.91, 121.82, 121.57, 121.17, 121.06, 120.47, 119.41, 119.40, 119.36, 118.83, 118.67, 113.15, 110.30, 105.81, 35.21, 35.11, 31.80, 31.65.

MS (HR-FAB) m/z: Found 998.5409 [(M + H)^+^]. Calculated for C_74_H_67_N_3_: 997.5335.

Figure S1. ^1^H NMR spectrum of intermediate 1.

Figure S2. ^1^H NMR spectrum of intermediate 2.

**Figure S3. ^1^H NMR spectrum of intermediate 3.**

**Figure S4. ^1^H NMR spectrum of intermediate 4.**

Figure S5. ^1^H NMR spectrum of intermediate 5.

**Figure S6. ^1^H NMR spectrum of intermediate 6.**

**Figure S7. ^1^H NMR spectrum of intermediate 7.**

**Figure S8. ^1^H NMR spectrum of intermediate 8.**

**Figure S9. ^1^H NMR spectrum of intermediate 9.**

**Figure S10. ^1^H NMR spectrum of intermediate 10.**

**Figure S11. ^1^H NMR spectrum of intermediate 11.**

**Figure S12. ^1^H NMR spectrum of intermediate 13.**

**Figure S13. ^1^H NMR spectrum of intermediate 14.**

Figure S14. ^1^H NMR spectrum of intermediate 15.

**Figure S15. ^1^H NMR spectrum of intermediate 16.**

**Figure S16.** **^1^H NMR spectrum of NBisICz.**

Figure S17. ^1^H NMR spectrum of NBisICz–PCz.

Figure S18. ^13^C NMR spectrum of NBisICz–PCz.

Figure S19. ^1^H NMR spectrum of NBisICz–DPA.

Figure S 20. ^13^C NMR spectrum of NBisICz–DPA.

**2. Quantum chemical calculation**

The quantum chemical calculations for characterizing electronic structure were performed with Gaussian 16W^[S2]^. The ground state (S_0_), 1^st^ singlet excited state (S_1_) and 1^st^ triplet excited state (T_1_) geometry were optimized using the hybrid version of the Becke’s three parameter exchange functional with the Lee-Yang-Parr correlation functional (B3LYP)^[S3-S6]^ at 6-31 G(d) level. The time-dependent-density functional theorgy (TD-DFT) calculation was performed at S_0_ geometry to determine vertical excitation energy. The natural transition orbitals (NTOs) of S_1_ state and triplet excited states were calculated at optimized S_1_ and T_1_ geometry, respectively. The spin-orbit coupling (SOC) matrix elements were calculated with ORCA software^[S7]^ using B3LYP functional at def2-split valence polarization (SVP) level^[S8]^. Molecular cubic volume was calculated using Multiwfn^[S9]^ based on optimized S_0_ geometry.

**3. Material characterization**

The ultraviolet-visible (UV-Vis) absorption spectra and photoluminescence (PL) emission spectra were measured with UV-Vis spectrophotometer (JASCO, V-730), fluorescence spectrophotometer (PerkinElmer, LS-55). The sampled solution for measuring fluorescence and phosphorescence spectra were prepared at 10^-5^ M in tetrahydrofuran (THF) and the spectra were measured without and with 1 ms delay at 77 K frozen THF solution.

Analysis of doped films were performed by measuring absolute photoluminescence quantum yield (PLQY), transient PL decay curves, and angle-dependent PL data. The doped film was prepared in 1,3-di(9*H*-carbazol-9-yl)benzene (mCP): diphenyl(4-(triphenylsilyl)phenyl)phosphine oxide (TSPO1) (50 wt%:50 wt%) mixed host and TTF-Phen on quartz substrate, and the thickness of each film was 40 nm. PLQY and transient PL meausurements were conducted under nitrogen saturated atomosphere using a Quantaurus-QY system (Hamamatsu, C11347-11) and a Quantaurus-Tau system (Hamamatsu, C11367-31). Angle-dependent PL equipment was constructed by combining a rotating stage and a half-cylinder lens, and the doped films used were encapsulated. The film was attached to a half-cylinder lens with refractive index matching oil. The excitation wavelength of the He/Cd laser source was 325 nm, and the angle-dependent p-polarized PL signal was collected with a spectrometer (MAYA 2000 Pro, OCEAN OPTICS Inc.). The rate constants were calculated using following formulas.

$\tau_{F}=\frac{1}{k_{F}}$ ∙∙∙ (1)

$k_{\mathrm{ISC}}=\left( 1-\emptyset_{\mathrm{PL}} \right)\cdot k_{F}$ ∙∙∙ (2)

$k_{r}^{S}=\emptyset_{\mathrm{PL}}\cdot k_{F}$ ∙∙∙ (3)

Where, *τ*_F_ represents fluorescence decay time, *k*_F_ represents rate constant of fluorescence, *Ф*_PL_ represents PLQY, *k*_ISC_ represents rate constant of intersystem crossing, *k*_r_^S^ represents rate constant radiative singlet emission transition.

Decomposition temeperature (*T*_d_), which was defined as the temperature at which 5% weigh loss occurred, was measured using thermogravimetric analyser (Hitachi High-Tech, TG/DTA7300). The thermogravimetric thermogram was recorded by scanning the weight of sample from room temperature to 600 °C under nitrogen atmosphere.

To characterize the energy level of the highest occupied molecular orbital (HOMO), cyclic voltammetry analysis (Ivium Tech., Iviumstat) was conducted to obtain oxidation curve. Two Pt wire electrodes were used as a working and counter electrode, respectively, and Ag/AgCl saturated in KCl electrode was used as a reference electrode. Tetrabutylammonium perchlorate and methylene chloride were selected as the electrolyte and solution matrix, and a measurement solution was prepared as a mixture of 0.1 M electrolyte and 10^-3^ M sample. HOMO level was characterized at onset voltage of the oxidation curve. Energy level of the lowest unoccupied molecular orbital (LUMO) was estimated using HOMO level and optical bandgap which was obtained from the edge position of UV-Vis absorption spectrum.

**4. Device characterization**

As a substrate for blue OLEDs, a transparent glass substrate with an indium tin oxide (ITO) anode was used. As a pretreatment of the manufacturing process, the ITO substrate was cleaned with acetone, chloroform, isopropyl alcohol, and deionized water. After drying, oxygen plasma treatment was performed. 40 nm of PEDOT:PSS film on ITO was prepared using spin-coating method (30 sec at 3,200 rpm) and after annealed at 150 °C for 15 minutes. The thermal evaporation process was performed under a high vacuum atmosphere of 10^-7^ Torr, and the organic layer and Al cathode were deposited and then encapsulated using a glass lid. The current density–volatge–luminance characteristics of the fabricated devices were evaluated using a spectroradiometer (Konica Minolta, CS-2000) and source meter (Keithley 2400). EQE was calculated assuming Lambertian distribution.

**5. Supplementary figures**


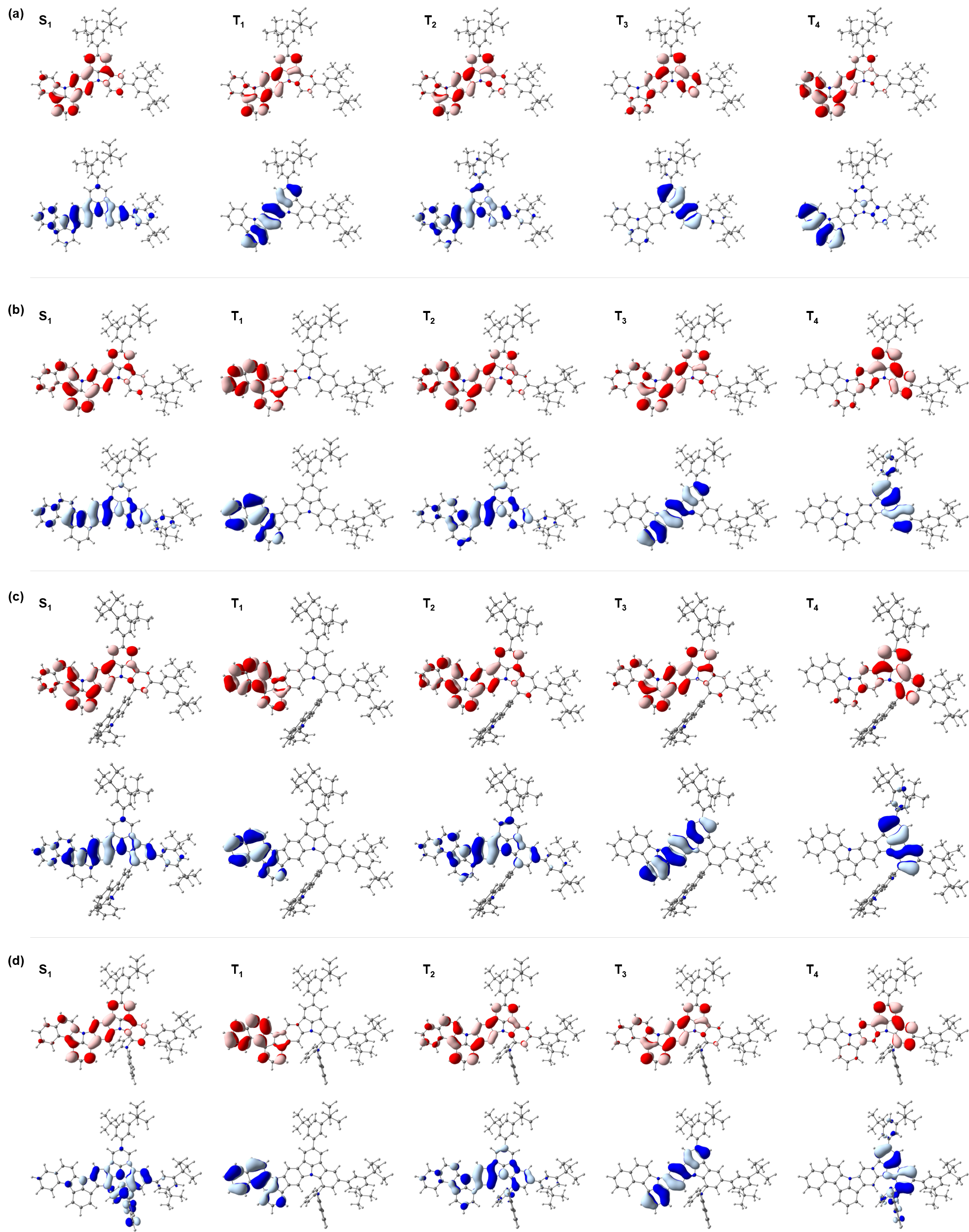


Figure S21. NTO distributions of (a) BisICz-Ref, (b) NBisICz, (c) NBisICz-PCz and (d) NBisICz-DPA corresponding to S_1_, T_1_ ~ T_4_ excited state (blue occupied: HONTO; red occupied: LUNTO).


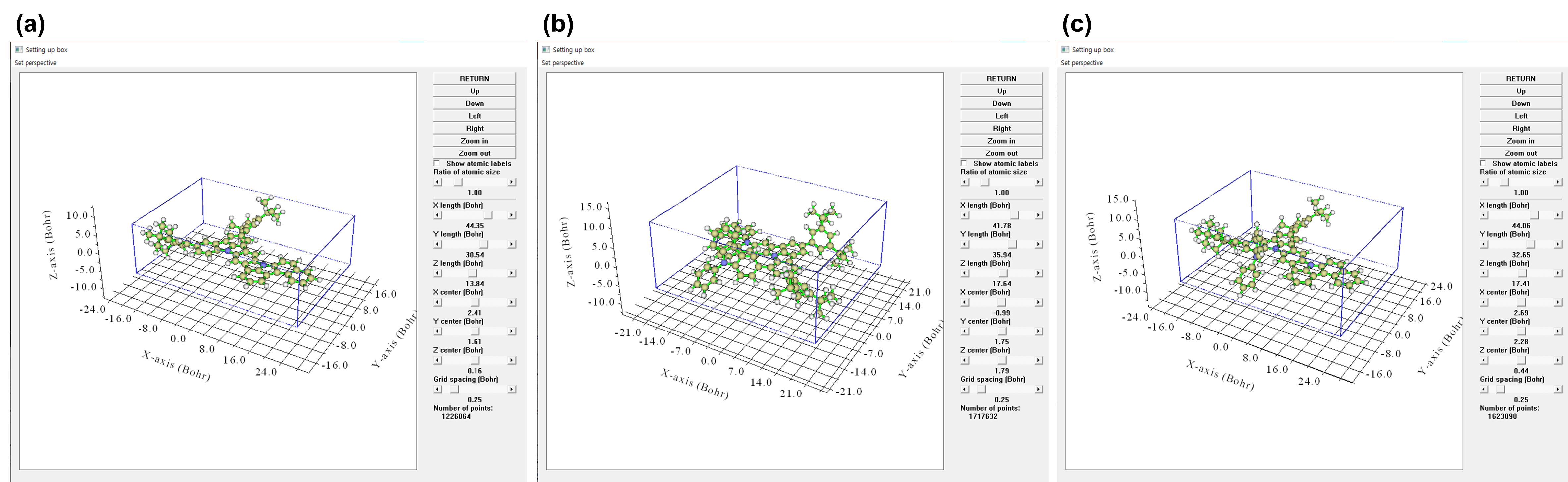


Figure S22. The molecular cubic specification calculated at optimized S_0_ geometry of (a) NBisICz, (b) NBisICz-PCz and (c) NBisICz-DPA.

Figure S23. The cyclic voltammogram of NBisICz (black), NBisICz-PCz (blue) and NBisICz-DPA (red).

Figure S24. Solvent-dependent PL spectra of (a) NBisICz, (b) NBisICz-PCz and (c) NBisICz-DPA measured in *n*-hexane (black), toluene (red), tetrahydrofuran (blue), and methylene chloride (green) at room temperature.

Figure S25. Solid PL spectra of mCP:TSPO1 1 wt% doped films of (a) NBisICz, (b) NBisICz-PCz, (c) NBisICz-DPA and (d) *t*-DABNA according to PL aging time.

Figure S26. Angle-dependent PL data (blue dot) and fitting results (red) of NBisICz (a), NBisICz-PCz (b) and (c) NBisICz-DPA measured in TTF-Phen 3 wt% doped films.

Figure S27. TGA thermogram of NBisICz (black), NBisICz-PCz (blue) and NBisICz-DPA (sky blue).


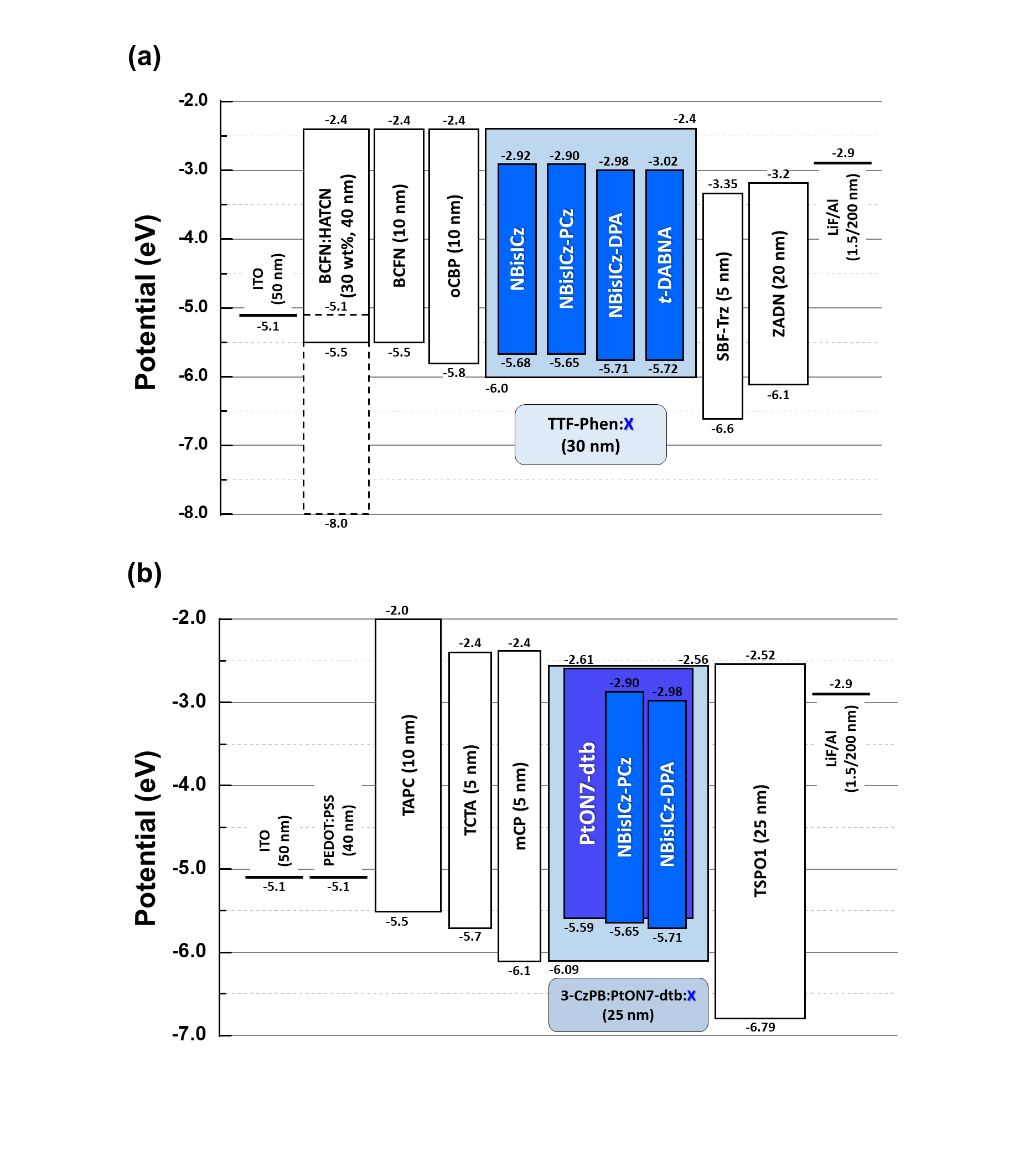


Figure S28. Energy level diagram of (a) TTF device and (b) PSF device.


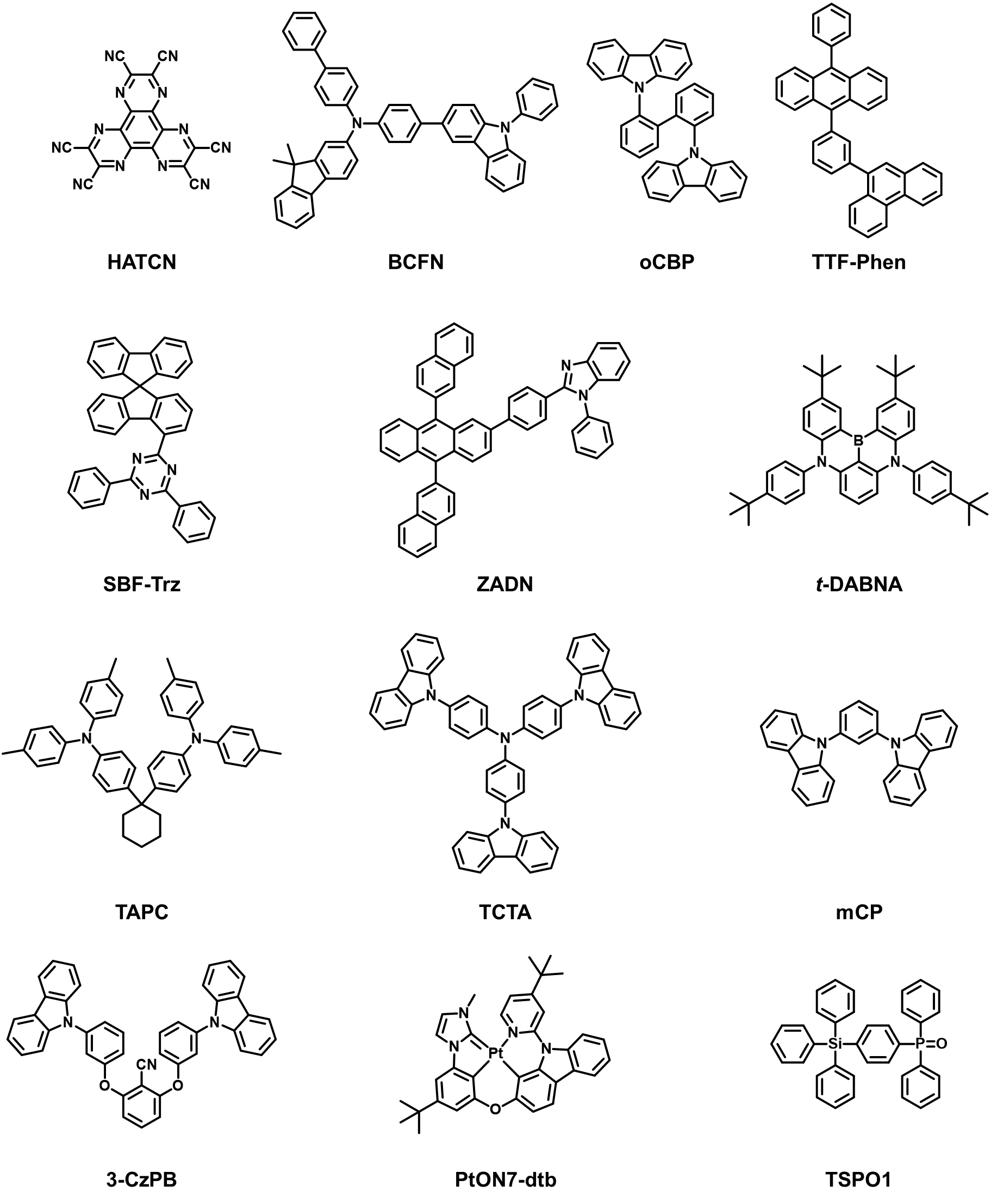


**Figure S29. Chemical structure of materials used in fabricated devices.**


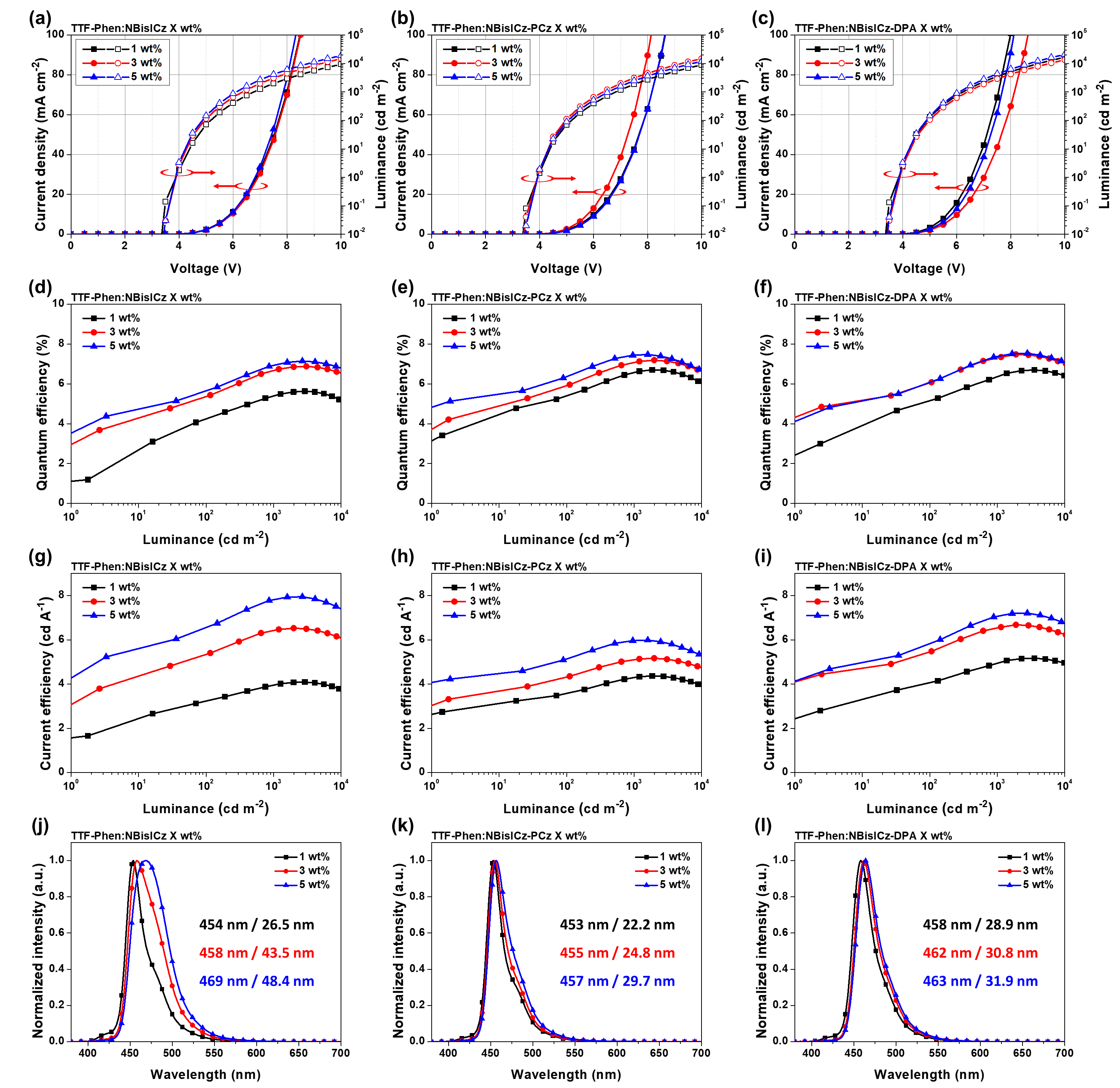


Figure S30. Device performance of TTF-Phen hosted devices at different doping concentration of NBisICz, NBisICz-PCz and NBisICz-DPA. (a), (b), (c) Current density–voltage–luminance curves, (d), (e), (f) EQE–luminance curves, (g), (h), (i) CE–luminance curves, (j), (k), (l) normalized EL spectra with λ_EL_ and FWHM (black: 1 wt%; red: 3 wt%; blue: 5 wt%).


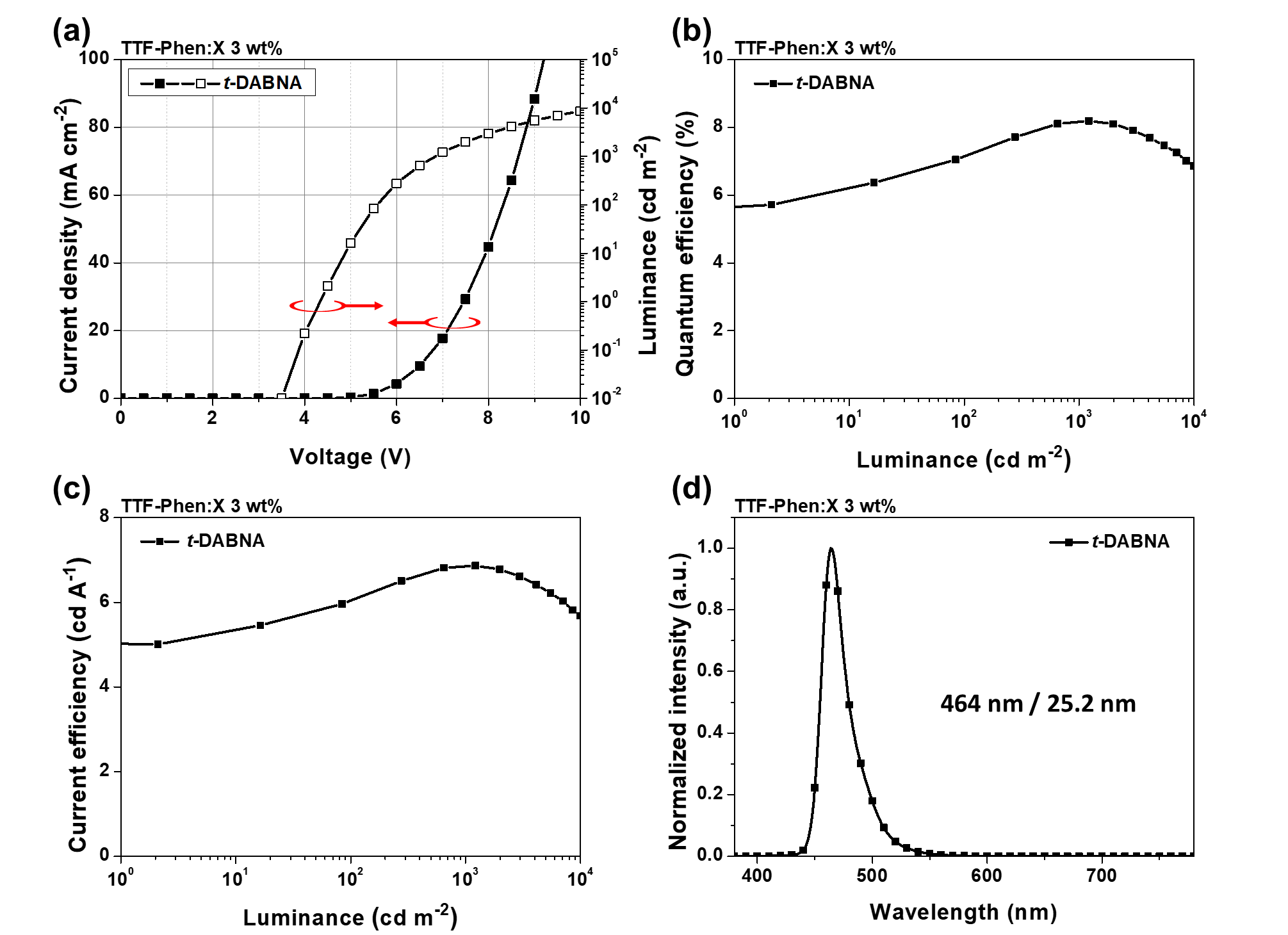


Figure S31. Device performance of TTF-Phen hosted *t*-DABNA 3 wt% doped device. (a) Current density–voltage–luminance curve, (b) EQE–luminance curve, (c) CE–luminance curve, (d) normalized EL spectrum with λ_EL_ and FWHM.


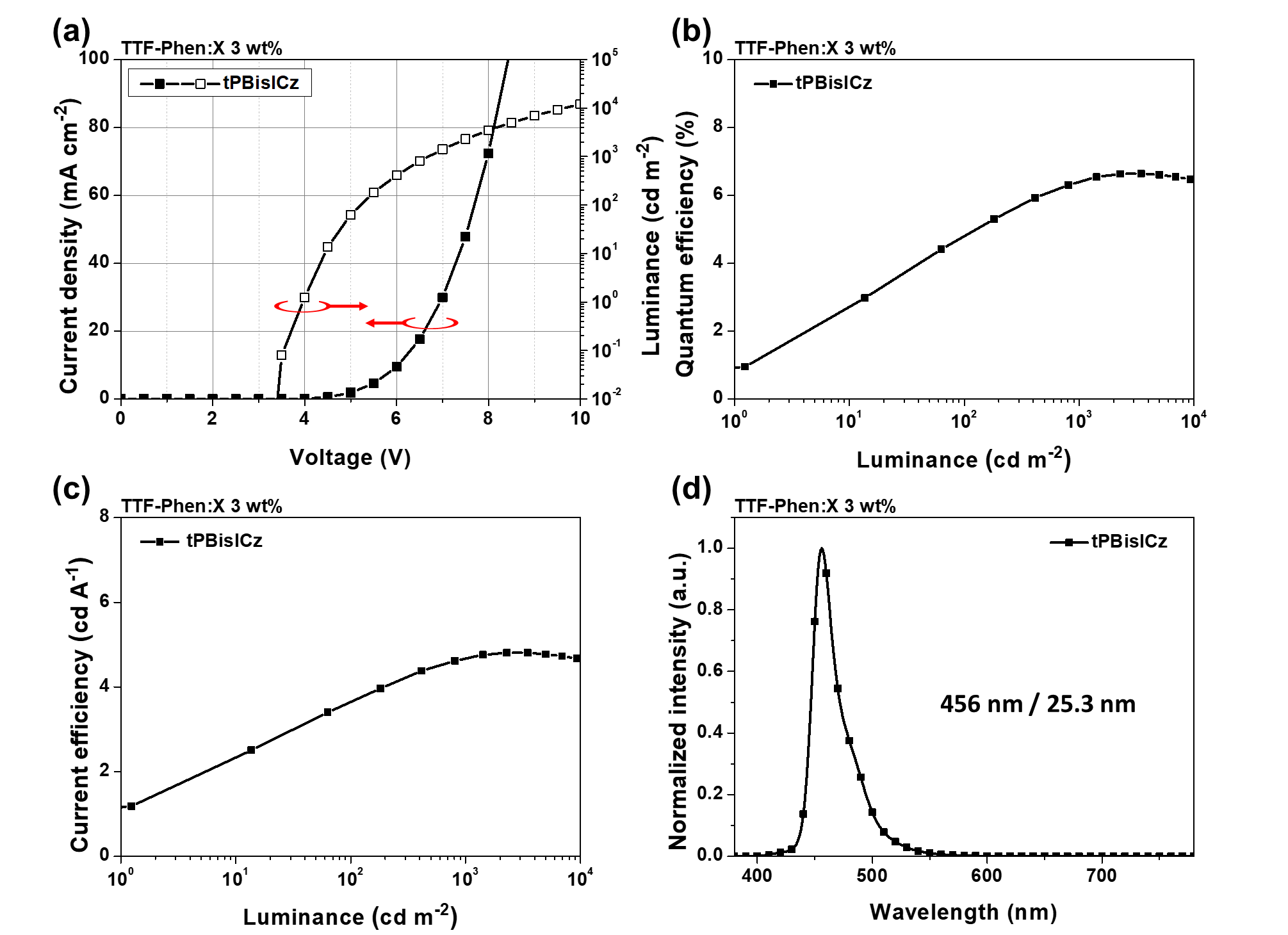


Figure S32. Device performance of TTF-Phen hosted tPBisICz 3 wt% doped device. (a) Current density–voltage–luminance curve, (b) EQE–luminance curve, (c) CE–luminance curve, (d) normalized EL spectrum with λ_EL_ and FWHM.

**6. Supplementary tables**


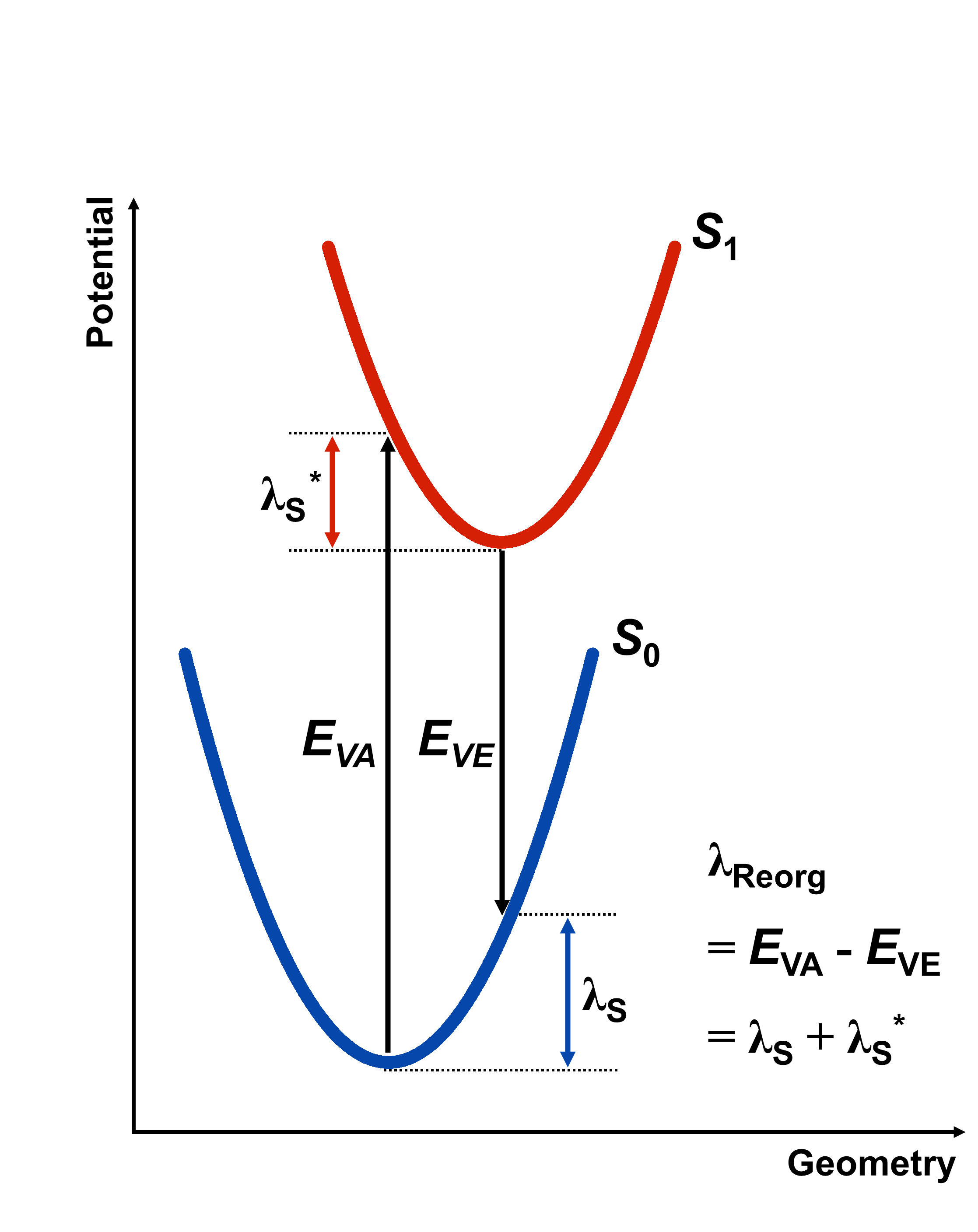


Table S1. The vertical excitation energy and reorganization energy of fused-ICz frameworks.

|  | **Vertical excitation energy**  **@ S_0_ geomtery (eV)** | | | | | | | **Reorganization energy**  **@ S_0_ and S_1_ geometry (eV)** | | |
| --- | --- | --- | --- | --- | --- | --- | --- | --- | --- | --- |
|  | **S_1_ (*f ^a^*)** | **T_1_** | **T_2_** | **T_3_** | **T_4_** | **T_5_** | **Δ*E*_ST_** | **λ_S_ *^b^*** | **λ_S_^*^ *^c^*** | **λ_Reorg_ *^d^*** |
| **BisICz-Ref** | 3.0145 (0.1533) | 2.6437 | 2.6815 | 2.9394 | 3.0529 | 3.1748 | 0.3708 | 0.1252 | 0.1063 | 0.2315 |
| **NBisICz** | 2.9434 (0.1636) | 2.4121 | 2.6558 | 2.6643 | 2.9319 | 3.0908 | 0.5313 | 0.1361 | 0.1167 | 0.2528 |
| **NBisICz-PCz** | 2.9413 (0.1682) | 2.4173 | 2.6494 | 2.6618 | 2.9107 | 3.0763 | 0.5240 | 0.1403 | 0.1201 | 0.2604 |
| **NBisICz-DPA** | 2.9034 (0.1647) | 2.4152 | 2.6434 | 2.6476 | 2.8928 | 3.0574 | 0.4882 | 0.1640 | 0.1555 | 0.3195 |

*^a^* Oscillator strength. *^b^* Reorganization energy of ground state. *^c^* Reorganization energy of excited singlet state. *^d^* Total reorganization energy.

Table S2. The vertical excitation energy gap and spin-orbit coupling matrix element of fused-ICz frameworks.

|  | **BisICz-Ref** | | | **NBisICz** | | | **NBisICz-PCz** | | | **NBisICz-DPA** | | |
| --- | --- | --- | --- | --- | --- | --- | --- | --- | --- | --- | --- | --- |
|  | ***E*_S1_ – *E*_Tn_** | **ξ(T_n_ – S_1_)** | ***E*_Tn_ - *E*_T1_** | ***E*_S1_ – *E*_Tn_** | **ξ(T_n_ – S_1_)** | ***E*_Tn_ - *E*_T1_** | ***E*_S1_ – *E*_Tn_** | **ξ(T_n_ – S_1_)** | ***E*_Tn_ - *E*_T1_** | ***E*_S1_ – *E*_Tn_** | **ξ(T_n_ – S_1_)** | ***E*_Tn_ - *E*_T1_** |
| **n** | **(eV) *^a^*** | **(cm^-1^) *^b^*** | **(eV) *^c^*** | **(eV) *^a^*** | **(cm^-1^) *^b^*** | **(eV) *^c^*** | **(eV) *^a^*** | **(cm^-1^) *^b^*** | **(eV) *^c^*** | **(eV) *^a^*** | **(cm^-1^) *^b^*** | **(eV) *^c^*** |
| **1** | 0.3708 | 0.006255 | 0 | 0.5313 | 0.002301 | 0 | 0.5240 | 0.007253 | 0 | 0.4882 | 0.073347 | 0 |
| **2** | 0.3330 | 0.042223 | 0.0378 | 0.2876 | 0.008645 | 0.2437 | 0.2919 | 0.008829 | 0.2321 | 0.2600 | 0.059065 | 0.2282 |
| **3** | 0.0751 | 0.086602 | 0.2957 | 0.2791 | 0.036838 | 0.2522 | 0.2795 | 0.050288 | 0.2445 | 0.2558 | 0.076218 | 0.2324 |
| **4** | -0.0384 | 0.030002 | 0.4092 | 0.0115 | 0.047176 | 0.5198 | 0.0306 | 0.077275 | 0.4934 | 0.0106 | 0.141719 | 0.4776 |
| **5** | -0.1603 | 0.008894 | 0.5311 | -0.1474 | 0.053676 | 0.6787 | -0.1350 | 0.094896 | 0.6590 | -0.1540 | 0.172522 | 0.6422 |

*^a^* Vertical excitation energy gap between S_1_ and T_n_ state. *^b^* Spin-orbit coupling matrix element between S_1_ and T_n_ state without vibronic coupling contribution. *^c^* Vertical excitation energy gap between T_1_ and T_n_ state.

Table S3. The molecular cubic specifications and molecular volume calculated at optimized S_0_ geometry of NBisICz, NBisICz–PCz, and NBisICz–DPA.

|  | **x** | **y** | **z** | **Molecular cubic volume** | |
| --- | --- | --- | --- | --- | --- |
|  | **(Bohr)** | **(Bohr)** | **(Bohr)** | **(Bohr^3^)** | **(nm^3^)** |
| **NBisICz** | 44.35 | 30.54 | 13.84 | 18745.57416 | 2.778 |
| **NBisICz–PCz** | 41.78 | 35.94 | 17.64 | 26487.75125 | 3.925 |
| **NBisICz–DPA** | 44.06 | 32.65 | 17.41 | 25045.31219 | 3.711 |

1 Bohr = 0.0529177249 nm

Table S4. Summarized device performance of blue phosphor-sensitized fluorescence OLEDs by terminal emitter type.

| **Terminal emitter type** | **Name** | **Phosphor**  **sensitizer** | **λ_EL_** | **FWHM** | **EQE_Max_** | **CE_Max_** | **CIE**  **(x, y)** | **Blue index** | **Ref.** |
| --- | --- | --- | --- | --- | --- | --- | --- | --- | --- |
|  |  |  | **(nm)** | **(nm)** | **(%)** | **(cd A^-1^)** |  | **(cd A^-1^)** |  |
| **MR-fluorescent** | NBisICz-PCz | PtON7-dtb | 453 | 22.0 | 18.8 | 16.6 | (0.143, 0.076) | 218.4 | This |
|  | NBisICz-DPA | PtON7-dtb | 454 | 27.5 | 20.6 | 19.1 | (0.141, 0.085) | 224.7 | work |
| **Conventional**  **fluorescent** | TBPe | 11 | 461 | - | 15.89 | 24.34 | (0.15, 0.20) | 121.7 | [S10] |
|  | TBPDP | PtON7-dtb | 474 | 56 | 17.9 | 27.3 | (0.131, 0.223) | 122.4 | [S11] |
|  | TBPe | (difysipy)_2_  Ir(mpic) | - | - | 15.3 | 18.0 | (0.14, 0.19) | 94.7 | [S12] |
| **MR-TADF** | mBP-DABNA-Me | Pt-AdaPh | 470 | 29.0 | 25.0 | 31.5 | (0.133, 0.174) | 181.0 | [S13] |
|  | *ν*-DABNA | Pt-dipCz | 470 | 18 | 31.4 | 28.9 | (0.124, 0.116) | 249.1 | [S14] |
|  | *ν*-DABNA | *f*-Ir(tBpp)_3_ | 474 | 19 | 18.9 | 20.1 | (0.12, 0.15) | 134.0 | [S15] |
|  | *t*-DABNA | *f*-Ir(tBpp)_3_ | 462 | 29 | 18.1 | 15.7 | (0.14, 0.10) | 157.0 | [S15] |
|  | *ν*-DABNA | *f*-Ir(ptBp)_3_ | 473 | 20 | 16.3 | 15.1 | (0.12, 0.13) | 116.2 | [S15] |
|  | *t*-DABNA | *f*-Ir(ptBp)_3_ | 461 | 33 | 14.1 | 11.9 | (0.14, 0.10) | 119.0 | [S15] |
|  | *ν*-DABNA | *f*-CN1 | 472 | 18 | 30.3 | 33.8 | (0.12, 0.16) | 211.3 | [S16] |
|  | m-DINBO | *f*-CN1 | 466 | 22 | 30.8 | 26.5 | (0.13, 0.10) | 265.0 | [S16] |
|  | *ν*-DABNA | *f*-CN2 | 472 | 18 | 30.6 | 38.2 | (0.13, 0.18) | 212.2 | [S16] |
|  | m-DINBO | *f*-CN2 | 466 | 20 | 30.1 | 31.8 | (0.16, 0.13) | 244.6 | [S16] |
|  | *ν*-DABNA | Irtb1 | 472 | 24 | 23.5 | 25.0 | (0.12, 0.15) | 166.7 | [S17] |
|  | *ν*-DABNA | *f*-ct5mix | 467 | 17 | 32.0 | 25.4 | (0.127, 0.098) | 259.2 | [S18] |
|  | *ν*-DABNA | *f*-ct6a | 472 | 22 | 26.2 | 25.0 | (0.12, 0.13) | 192.3 | [S19] |
|  | *ν*-DABNA | *f*-ct6b | 472 | 22 | 25.1 | 23.3 | (0.12, 0.13) | 179.2 | [S19] |
|  | *ν*-DABNA | *f*-ct6c | 472 | 22 | 25.8 | 25.0 | (0.12, 0.13) | 192.3 | [S19] |
|  | *ν*-DABNA | Pt-tmCyCz | 472 | 19 | 33.9 | 30.8 | (0.116, 0.123) | 250.4 | [S20] |
|  | *t*-DABNA | *f*-ct7a | 460 | 26.6 | 32.5 | 26.0 | (0.14, 0.09) | 288.9 | [S21] |
|  | *t*-DABNA | *f*-ct7b | 460 | 26.5 | 36.1 | 23.5 | (0.14, 0.09) | 261.1 | [S21] |
|  | *t*-DABNA | *f*-ct7c | 460 | 27.0 | 26.4 | 20.3 | (0.14, 0.09) | 225.6 | [S21] |
|  | *ν*-DABNA | Β–4–TMS | 467 | 18 | 29.50 | 24.77 | (0.127, 0.097) | 255.4 | [S22] |
|  | *ν*-DABNA | Β–5–TMS | 467 | 18 | 31.06 | 26.91 | (0.126, 0.098) | 274.6 | [S22] |
|  | *ν*-DABNA | Β–4–TMS | 470 | 17 | 33.43 | 31.06 | (0.119, 0.123) | 252.5 | [S22] |
|  | *ν*-DABNA | Β–5–TMS | 470 | 17 | 33.42 | 30.79 | (0.119, 0.123) | 250.3 | [S22] |
|  | *ν*-DABNA | *f*-Ir(mfcp)_3_ | 473 | - | 16.70 | 11.40 | (0.13, 0.18) | 63.3 | [S23] |
|  | *ν*-DABNA | *f*-Ir(mfcp)_3_ | 474 | - | 15.50 | 10.30 | (0.12, 0.17) | 60.6 | [S23] |
|  | *t*-DABNA | *f*-Ir(mfcp)_3_ | 463 | - | 16.10 | 9.70 | (0.14, 0.18) | 53.9 | [S23] |
|  | *t*-DABNA | *f*-Ir(mfcp)_3_ | 463 | - | 15.30 | 8.40 | (0.14, 0.15) | 56.0 | [S23] |
|  | *ν*-DABNA | *f*-Ir(5-mfcp)_3_ | 473 | - | 18.80 | 13.10 | (0.13, 0.20) | 65.5 | [S23] |
|  | *ν*-DABNA | *f*-Ir(5-mfcp)_3_ | 475 | - | 17.10 | 11.40 | (0.12, 0.18) | 63.3 | [S23] |
|  | *t*-DABNA | *f*-Ir(5-mfcp)_3_ | 465 | - | 16.80 | 10.40 | (0.15, 0.23) | 45.2 | [S23] |
|  | *t*-DABNA | *f*-Ir(5-mfcp)_3_ | 465 | - | 16.00 | 9.05 | (0.15, 0.18) | 50.3 | [S23] |
|  | *t*-DABNA | PtON-TBBI | - | - | 23.7 *^a^* | 26.2 *^a^* | (-, 0.171) | 153.2 | [17] |
|  | TBE01 | PtON-TBBI | - | - | 25.4 *^a^* | 27.1 *^a^* | (-, 0.165) | 164.2 | [17] |
|  | TBE02 | PtON-TBBI | - | - | 25.8 *^a^* | 27.7 *^a^* | (-, 0.165) | 167.9 | [17] |
|  | *t*-DABNA | *f*-CF_3_ | 463 | 26 | 16.0 | 14.2 | (0.13, 0.10) | 142.0 | [S24] |
|  | *ν*-DABNA | Complex 3 | 471 | - | 15.30 | 27.23 | (0.15, 0.27) | 100.9 | [S25] |
|  | *ν*-DABNA | Complex 6 | 472 | - | 15.76 | 21.78 | (0.13, 0.19) | 114.6 | [S25] |
|  | *t*-DABNA | *f*-tpb1 | 462 | 30 | 29.6 | 28.7 | (0.13, 0.11) | 260.9 | [S26] |
|  | *ν*-DABNA | PtON7-dtb | 473 | 20 | 32.2 | 32.0 | (0.111, 0.141) | 227.0 | [S11] |
|  | *t*-DABNA | *m*-tz1 | 468 | 31 | 16.7 | 20.0 | (0.14, 0.15) | 133.3 | [S27] |
|  | *t*-DABNA | *m*-tz2 | 468 | 31 | 19.7 | 20.0 | (0.12, 0.13) | 153.8 | [S27] |
|  | *t*-DABNA | Ir(cb)_3_ | - | - | 24.8 | 22.6 | (0.131, 0.107) | 211.2 | [S28] |

*^a^* Values at 1000 cd m^-2^.

Table S5. The Cartesian coordinates of optmized S_0_, S_1_ and T_1_ geometry for BisICz-Ref.

|  | **X (Å)** | **Y (Å)** | **Z (Å)** |
| --- | --- | --- | --- |
| ***Optimized S_0_ geometry*** | | | |
| C | 0.636122061 | 6.420598104 | 3.463174005 |
| C | 0.858712115 | 6.881396498 | 2.002879906 |
| C | 0.653930441 | 8.407411919 | 1.948172980 |
| C | 2.318956900 | 6.580419794 | 1.588910852 |
| C | -0.106146861 | 6.120530791 | 1.071606858 |
| C | -0.039910218 | 4.723946278 | 0.999288157 |
| C | -0.900098718 | 3.986395724 | 0.174273721 |
| C | -0.809016206 | 2.500136098 | 0.112452135 |
| C | -2.008589548 | 1.738718624 | 0.111582619 |
| C | -1.937553689 | 0.341275466 | 0.050642910 |
| C | -2.769350325 | -0.867537225 | 0.027686105 |
| C | -1.880881367 | -2.007284497 | -0.041578869 |
| C | -2.343467940 | -3.316849971 | -0.082110455 |
| C | -3.726519899 | -3.513719005 | -0.052393922 |
| C | -4.557747542 | -4.721888212 | -0.078186017 |
| C | -5.851800931 | -4.227079401 | -0.022565430 |
| N | -5.933012547 | -2.865753349 | 0.034558256 |
| C | -7.077667070 | -4.874635167 | -0.014717898 |
| C | -8.044384526 | -3.771661417 | 0.056669659 |
| C | -7.297874551 | -2.540006926 | 0.085767400 |
| C | -7.929765104 | -1.302359260 | 0.153709896 |
| C | -9.326016050 | -1.284337478 | 0.193360626 |
| C | -10.071719347 | -2.470641865 | 0.165909153 |
| C | -9.438075207 | -3.712731948 | 0.097875807 |
| C | -6.978767493 | -6.272543359 | -0.073780405 |
| C | -5.694490347 | -6.855330179 | -0.132757403 |
| C | -4.488939016 | -6.121938826 | -0.136493065 |
| C | -4.614742498 | -2.374310666 | 0.017839694 |
| C | -4.152096138 | -1.064472718 | 0.057377058 |
| C | -0.645685167 | -0.155076813 | 0.000100634 |
| N | -0.562264908 | -1.515836337 | -0.056955350 |
| C | 0.575755420 | 0.497588151 | -0.000905166 |
| C | 1.547006353 | -0.603273457 | -0.075805657 |
| C | 0.803876435 | -1.835697065 | -0.104104965 |
| C | 1.449997617 | -3.065418026 | -0.165745481 |
| C | 2.844232271 | -3.072774368 | -0.200377833 |
| C | 3.607304811 | -1.886785725 | -0.172381939 |
| C | 5.092675080 | -1.942941369 | -0.208184291 |
| C | 5.789600115 | -2.927243089 | 0.499319040 |
| C | 7.191157545 | -2.998751546 | 0.478327245 |
| C | 7.909014423 | -4.099102667 | 1.284317401 |
| C | 7.565645383 | -3.944807356 | 2.785175979 |
| C | 7.438262523 | -5.488464022 | 0.792150629 |
| C | 9.441133322 | -4.035764162 | 1.136331337 |
| C | 7.878847404 | -2.044229932 | -0.276409756 |
| C | 7.219311309 | -1.034927784 | -1.001953951 |
| C | 8.047063009 | -0.023454553 | -1.818778278 |
| C | 9.002039398 | 0.743826496 | -0.873392153 |
| C | 8.877949661 | -0.776210884 | -2.885172900 |
| C | 7.164079121 | 1.009659740 | -2.544359880 |
| C | 5.825060600 | -1.003161713 | -0.955782045 |
| C | 2.938578335 | -0.652566582 | -0.109894496 |
| C | 0.474940994 | 1.892787802 | 0.056172193 |
| C | -1.847915540 | 4.679098347 | -0.591508704 |
| C | -1.953129434 | 6.074456069 | -0.547839325 |
| C | -3.007456522 | 6.786226880 | -1.419030413 |
| C | -2.734134235 | 6.480425985 | -2.911064717 |
| C | -4.418253202 | 6.274185925 | -1.042577200 |
| C | -2.992322191 | 8.315811126 | -1.235228850 |
| C | -1.071191687 | 6.772519126 | 0.290047681 |
| H | 1.320566545 | 6.948249577 | 4.139000002 |
| H | 0.811139413 | 5.346507992 | 3.583296410 |
| H | -0.389801394 | 6.629560781 | 3.787350946 |
| H | 1.365162550 | 8.898483326 | 2.622023187 |
| H | -0.354198607 | 8.696450490 | 2.266224461 |
| H | 0.823298975 | 8.806940795 | 0.941750360 |
| H | 3.019572866 | 7.110501565 | 2.245892778 |
| H | 2.508475514 | 6.903273797 | 0.558733645 |
| H | 2.548495316 | 5.511615633 | 1.651447611 |
| H | 0.675379673 | 4.183864162 | 1.612742598 |
| H | -2.957542296 | 2.261643956 | 0.182769893 |
| H | -1.660465173 | -4.158467935 | -0.134533524 |
| H | -7.360398536 | -0.378654260 | 0.175709627 |
| H | -9.840580175 | -0.328974836 | 0.246685618 |
| H | -11.156335166 | -2.422362902 | 0.198147811 |
| H | -10.024295646 | -4.627525828 | 0.077185142 |
| H | -7.855854653 | -6.913652550 | -0.075333968 |
| H | -5.629949667 | -7.939047296 | -0.178222199 |
| H | -3.541997994 | -6.652471192 | -0.184037266 |
| H | -4.834739510 | -0.222635564 | 0.110051698 |
| H | 0.892602557 | -3.996193192 | -0.197853393 |
| H | 3.359551537 | -4.025481057 | -0.277267217 |
| H | 5.223248778 | -3.633589479 | 1.099147142 |
| H | 6.489115551 | -4.025211605 | 2.968014894 |
| H | 7.896394869 | -2.971024574 | 3.164086702 |
| H | 8.063514495 | -4.725770235 | 3.373204607 |
| H | 6.357888378 | -5.619500163 | 0.912955688 |
| H | 7.935759147 | -6.283916013 | 1.360633867 |
| H | 7.675466913 | -5.629344764 | -0.268619083 |
| H | 9.848299019 | -3.086405794 | 1.502446358 |
| H | 9.756673354 | -4.165091181 | 0.094719075 |
| H | 9.901280193 | -4.839217217 | 1.722615453 |
| H | 8.961377524 | -2.078173891 | -0.308008549 |
| H | 9.692819970 | 0.070589662 | -0.355253032 |
| H | 8.439168587 | 1.295386471 | -0.111619949 |
| H | 9.602831050 | 1.465100140 | -1.441082293 |
| H | 9.565350167 | -1.498423177 | -2.432507675 |
| H | 9.476610899 | -0.068876133 | -3.472381366 |
| H | 8.225204603 | -1.323400422 | -3.574899838 |
| H | 6.564249407 | 1.598989038 | -1.841466875 |
| H | 6.483799860 | 0.535072802 | -3.260503034 |
| H | 7.797148311 | 1.707726077 | -3.103754701 |
| H | 5.276599502 | -0.261038230 | -1.524112754 |
| H | 3.513265050 | 0.268027440 | -0.063308421 |
| H | 1.352849596 | 2.531336283 | 0.034962588 |
| H | -2.494485189 | 4.108321289 | -1.251714394 |
| H | -2.775627139 | 5.406771755 | -3.121566611 |
| H | -1.742729258 | 6.840447122 | -3.208954397 |
| H | -3.480377965 | 6.974526243 | -3.545601849 |
| H | -4.514414408 | 5.194286420 | -1.196203690 |
| H | -5.180795199 | 6.767322499 | -1.658070503 |
| H | -4.643055603 | 6.483528657 | 0.009593754 |
| H | -2.028718705 | 8.752553174 | -1.521092961 |
| H | -3.205858916 | 8.605300873 | -0.199965587 |
| H | -3.761121011 | 8.770447832 | -1.870326540 |
| H | -1.137367123 | 7.850722321 | 0.334879188 |
| ***Optimized S_1_ geometry*** | | | |
| C | 0.661824300 | 6.463746476 | 3.417595720 |
| C | 0.855692291 | 6.922536748 | 1.952646967 |
| C | 0.640122667 | 8.447076770 | 1.898240434 |
| C | 2.310524381 | 6.630225163 | 1.514143441 |
| C | -0.120079286 | 6.152944379 | 1.040007939 |
| C | -0.046181558 | 4.756902225 | 0.970485642 |
| C | -0.916723298 | 4.010398235 | 0.163892592 |
| C | -0.817098685 | 2.524762775 | 0.104956674 |
| C | -2.022654826 | 1.766416205 | 0.106167426 |
| C | -1.947854907 | 0.354223346 | 0.046273314 |
| C | -2.759264894 | -0.848455855 | 0.026283987 |
| C | -1.859463664 | -2.008312360 | -0.041688938 |
| C | -2.295147771 | -3.330787187 | -0.078094558 |
| C | -3.674215845 | -3.564066886 | -0.047232104 |
| C | -4.486458567 | -4.765011782 | -0.068211972 |
| C | -5.786287518 | -4.285771852 | -0.012272832 |
| N | -5.868696714 | -2.902757539 | 0.041768644 |
| C | -7.007180448 | -4.923825812 | -0.000165659 |
| C | -7.970234675 | -3.823489910 | 0.069385798 |
| C | -7.230761382 | -2.580581386 | 0.094134656 |
| C | -7.861715026 | -1.341790548 | 0.159460812 |
| C | -9.257934592 | -1.320990993 | 0.201564126 |
| C | -9.996013874 | -2.514342057 | 0.178480101 |
| C | -9.364136392 | -3.759668731 | 0.113008235 |
| C | -6.912489524 | -6.337962791 | -0.055666246 |
| C | -5.626497367 | -6.906653229 | -0.114640739 |
| C | -4.415652867 | -6.177969383 | -0.122985915 |
| C | -4.575821417 | -2.404949362 | 0.022278942 |
| C | -4.137882734 | -1.080849140 | 0.058163689 |
| C | -0.649178520 | -0.120107441 | -0.004108504 |
| N | -0.561981074 | -1.509291054 | -0.059224870 |
| C | 0.564993479 | 0.521728588 | -0.007356269 |
| C | 1.530561503 | -0.577296924 | -0.081774964 |
| C | 0.787129451 | -1.825304859 | -0.107337388 |
| C | 1.432608704 | -3.061387675 | -0.165885923 |
| C | 2.822502232 | -3.077037541 | -0.197268941 |
| C | 3.584808475 | -1.881565217 | -0.175487453 |
| C | 5.061836609 | -1.935956600 | -0.207412109 |
| C | 5.755491254 | -2.979326566 | 0.421396104 |
| C | 7.155381564 | -3.048035892 | 0.408222126 |
| C | 7.868462671 | -4.207151321 | 1.131769740 |
| C | 7.514823993 | -4.167452651 | 2.637682516 |
| C | 7.401241865 | -5.554292772 | 0.530837484 |
| C | 9.401389200 | -4.131713982 | 0.998417057 |
| C | 7.847861219 | -2.036241198 | -0.265022889 |
| C | 7.195136428 | -0.970414722 | -0.914316854 |
| C | 8.030874260 | 0.098152914 | -1.645064956 |
| C | 8.978774067 | 0.788310405 | -0.635150206 |
| C | 8.869066045 | -0.574250879 | -2.758493634 |
| C | 7.154893821 | 1.184824982 | -2.296915884 |
| C | 5.802744031 | -0.937082318 | -0.871336130 |
| C | 2.917108429 | -0.637115485 | -0.119444004 |
| C | 0.470549692 | 1.936084037 | 0.049010075 |
| C | -1.882815023 | 4.695593009 | -0.586368736 |
| C | -1.993962745 | 6.090377319 | -0.547442865 |
| C | -3.067136842 | 6.792785768 | -1.403158054 |
| C | -2.818365986 | 6.481392950 | -2.898300808 |
| C | -4.468461804 | 6.275191362 | -0.999977331 |
| C | -3.056938516 | 8.323290093 | -1.226781361 |
| C | -1.101871283 | 6.797032680 | 0.272579380 |
| H | 1.354683116 | 6.997045275 | 4.080443893 |
| H | 0.845209903 | 5.390942053 | 3.536346730 |
| H | -0.359607098 | 6.666971178 | 3.759124308 |
| H | 1.359467243 | 8.944506189 | 2.558800794 |
| H | -0.364404012 | 8.730084255 | 2.232688858 |
| H | 0.789448179 | 8.845393195 | 0.888151399 |
| H | 3.019269489 | 7.165600771 | 2.158173812 |
| H | 2.480024452 | 6.952698740 | 0.480332240 |
| H | 2.547123403 | 5.562809086 | 1.573871486 |
| H | 0.682877067 | 4.221419342 | 1.571270146 |
| H | -2.970446326 | 2.286991027 | 0.189886407 |
| H | -1.590379223 | -4.154444987 | -0.128451691 |
| H | -7.290691340 | -0.419005231 | 0.177622168 |
| H | -9.775390142 | -0.367736970 | 0.252917319 |
| H | -11.081070815 | -2.469384714 | 0.212331554 |
| H | -9.953860937 | -4.671948300 | 0.096269632 |
| H | -7.788821601 | -6.978271019 | -0.053765432 |
| H | -5.557484240 | -7.991128286 | -0.157131509 |
| H | -3.469893582 | -6.708878312 | -0.170587692 |
| H | -4.842573535 | -0.257579008 | 0.109390699 |
| H | 0.869353192 | -3.988377413 | -0.197815879 |
| H | 3.335730835 | -4.029064688 | -0.278754263 |
| H | 5.186967105 | -3.730439801 | 0.960437926 |
| H | 6.437332329 | -4.263013680 | 2.807410276 |
| H | 7.842104882 | -3.225582019 | 3.092499106 |
| H | 8.009904394 | -4.990750298 | 3.166916054 |
| H | 6.320475652 | -5.695871658 | 0.635639028 |
| H | 7.896515945 | -6.390308475 | 1.039400919 |
| H | 7.644424211 | -5.613538769 | -0.536201569 |
| H | 9.806036596 | -3.213928996 | 1.440050191 |
| H | 9.723995813 | -4.180684558 | -0.047919037 |
| H | 9.857937242 | -4.978024601 | 1.523637648 |
| H | 8.930375463 | -2.069102068 | -0.291932377 |
| H | 9.664728860 | 0.076438804 | -0.164255993 |
| H | 8.410610055 | 1.282121617 | 0.161406023 |
| H | 9.585215904 | 1.548899786 | -1.141719473 |
| H | 9.552234720 | -1.330033754 | -2.356979455 |
| H | 9.473525161 | 0.174640240 | -3.284539212 |
| H | 8.221623237 | -1.065472689 | -3.493780315 |
| H | 6.551787971 | 1.722357220 | -1.556300769 |
| H | 6.479146910 | 0.767402686 | -3.051943940 |
| H | 7.793526886 | 1.920841472 | -2.797877117 |
| H | 5.261997739 | -0.150960584 | -1.383930779 |
| H | 3.496954966 | 0.279102225 | -0.070391586 |
| H | 1.346715357 | 2.573790136 | 0.012772316 |
| H | -2.538794999 | 4.117025890 | -1.229899834 |
| H | -2.856868283 | 5.406426943 | -3.102354466 |
| H | -1.834453302 | 6.845799803 | -3.215240991 |
| H | -3.578601718 | 6.967868611 | -3.522152048 |
| H | -4.560996918 | 5.194040146 | -1.146360379 |
| H | -5.244338618 | 6.761156896 | -1.604554855 |
| H | -4.676016191 | 6.488527635 | 0.054938857 |
| H | -2.100497265 | 8.763597368 | -1.530797405 |
| H | -3.254365176 | 8.616521495 | -0.189354600 |
| H | -3.838751742 | 8.771102160 | -1.850832719 |
| H | -1.173796722 | 7.875049817 | 0.314964656 |
| ***Optimized T_1_ geometry*** | | | |
| C | 0.649244455 | 6.405708009 | 3.438414976 |
| C | 0.846526613 | 6.868791708 | 1.975223240 |
| C | 0.632704750 | 8.393714907 | 1.924818895 |
| C | 2.301623387 | 6.575707417 | 1.538017473 |
| C | -0.128829922 | 6.103501497 | 1.058674195 |
| C | -0.056707664 | 4.707508614 | 0.984370819 |
| C | -0.928174482 | 3.966355850 | 0.174048755 |
| C | -0.835592858 | 2.480687564 | 0.114994816 |
| C | -2.023747349 | 1.722672975 | 0.115602905 |
| C | -1.944973024 | 0.298165231 | 0.051029059 |
| C | -2.749417228 | -0.866208482 | 0.027718725 |
| C | -1.853913097 | -2.046873506 | -0.044280641 |
| C | -2.310013042 | -3.332144617 | -0.084429548 |
| C | -3.739666858 | -3.530794316 | -0.053508509 |
| C | -4.544232668 | -4.695831710 | -0.079186599 |
| C | -5.864689996 | -4.213443551 | -0.022808978 |
| N | -5.952523206 | -2.853070716 | 0.035590045 |
| C | -7.076687986 | -4.865459599 | -0.015789517 |
| C | -8.051892810 | -3.777577825 | 0.056286161 |
| C | -7.318890825 | -2.535321951 | 0.087041665 |
| C | -7.961094937 | -1.303596863 | 0.156177360 |
| C | -9.357186072 | -1.297603697 | 0.195641326 |
| C | -10.091086123 | -2.492958941 | 0.166668944 |
| C | -9.448584720 | -3.728924824 | 0.097488883 |
| C | -6.962306112 | -6.281364585 | -0.077046520 |
| C | -5.664769530 | -6.852626401 | -0.136555777 |
| C | -4.467736191 | -6.122922778 | -0.139860376 |
| C | -4.635236576 | -2.351057363 | 0.019511235 |
| C | -4.178380886 | -1.065161506 | 0.058810570 |
| C | -0.625011185 | -0.186018862 | -0.000573106 |
| N | -0.534956817 | -1.545055658 | -0.059399483 |
| C | 0.582677423 | 0.470738373 | -0.001361851 |
| C | 1.562165778 | -0.614747734 | -0.076478870 |
| C | 0.832339254 | -1.857650776 | -0.106972027 |
| C | 1.488254386 | -3.081602071 | -0.170339033 |
| C | 2.882140301 | -3.077415988 | -0.204348976 |
| C | 3.633698225 | -1.882142941 | -0.174365500 |
| C | 5.119725774 | -1.925924373 | -0.209129539 |
| C | 5.824447971 | -2.905060503 | 0.497890788 |
| C | 7.226595698 | -2.964680046 | 0.478487051 |
| C | 7.952714727 | -4.059866323 | 1.284136057 |
| C | 7.605860366 | -3.910518125 | 2.784686770 |
| C | 7.494680703 | -5.452604643 | 0.789536504 |
| C | 9.484466430 | -3.983241012 | 1.138591745 |
| C | 7.907236229 | -2.003680075 | -0.274405593 |
| C | 7.239813805 | -0.999420282 | -0.999731586 |
| C | 8.059834914 | 0.019697691 | -1.814884952 |
| C | 9.007647036 | 0.794073087 | -0.868081637 |
| C | 8.897870856 | -0.724993956 | -2.881344424 |
| C | 7.168931518 | 1.046141129 | -2.540257529 |
| C | 5.845294538 | -0.979572177 | -0.955148821 |
| C | 2.956777974 | -0.654238227 | -0.110416688 |
| C | 0.465861180 | 1.883793329 | 0.056670625 |
| C | -1.889988094 | 4.654754675 | -0.578437068 |
| C | -1.999268504 | 6.049554099 | -0.534380935 |
| C | -3.067762975 | 6.757219712 | -1.391482731 |
| C | -2.813118501 | 6.451752674 | -2.886886021 |
| C | -4.471458773 | 6.240024147 | -0.996000654 |
| C | -3.055615864 | 8.286880317 | -1.208166001 |
| C | -1.108075712 | 6.751029926 | 0.290965305 |
| H | 1.341279876 | 6.936710443 | 4.103724766 |
| H | 0.832239467 | 5.332558615 | 3.555046631 |
| H | -0.372581233 | 6.608747287 | 3.778865911 |
| H | 1.351360264 | 8.887999848 | 2.588303759 |
| H | -0.372031170 | 8.677125837 | 2.258320199 |
| H | 0.784901165 | 8.795048700 | 0.916369627 |
| H | 3.009583751 | 7.109078872 | 2.184310231 |
| H | 2.473312696 | 6.900224413 | 0.505254593 |
| H | 2.537929017 | 5.508122692 | 1.596313840 |
| H | 0.671498978 | 4.171079721 | 1.585545396 |
| H | -2.977362106 | 2.233570351 | 0.196417099 |
| H | -1.639364981 | -4.182675942 | -0.137498028 |
| H | -7.399351538 | -0.375042438 | 0.179257907 |
| H | -9.881154470 | -0.347531261 | 0.249914285 |
| H | -11.176245460 | -2.454390450 | 0.198789569 |
| H | -10.028037406 | -4.648038865 | 0.075811136 |
| H | -7.832068453 | -6.931009246 | -0.079450619 |
| H | -5.596785070 | -7.936403844 | -0.182929699 |
| H | -3.516346592 | -6.643389293 | -0.187699634 |
| H | -4.848745996 | -0.214474412 | 0.112241086 |
| H | 0.938258706 | -4.016913365 | -0.204144408 |
| H | 3.406597009 | -4.024803805 | -0.283312976 |
| H | 5.263227166 | -3.616972292 | 1.095941371 |
| H | 6.529771710 | -4.000194608 | 2.965731526 |
| H | 7.927767089 | -2.934424622 | 3.165288209 |
| H | 8.109464913 | -4.687984138 | 3.372512670 |
| H | 6.415239073 | -5.592832751 | 0.908210990 |
| H | 7.997920911 | -6.244552158 | 1.357909261 |
| H | 7.734893201 | -5.590124634 | -0.271002325 |
| H | 9.882877260 | -3.030791948 | 1.506323021 |
| H | 9.802670870 | -4.108638436 | 0.097307581 |
| H | 9.950654970 | -4.783373290 | 1.724682837 |
| H | 8.990067556 | -2.028501859 | -0.304911324 |
| H | 9.703513954 | 0.126097451 | -0.349940767 |
| H | 8.439545467 | 1.340175764 | -0.106250771 |
| H | 9.602937680 | 1.520877184 | -1.434562760 |
| H | 9.590968537 | -1.441797227 | -2.428770350 |
| H | 9.490991556 | -0.012156543 | -3.467570669 |
| H | 8.250237386 | -1.277114468 | -3.571961539 |
| H | 6.563465113 | 1.629570964 | -1.837279918 |
| H | 6.493264407 | 0.566537750 | -3.257410046 |
| H | 7.796637232 | 1.750090373 | -3.098375958 |
| H | 5.290931442 | -0.241754981 | -1.523325073 |
| H | 3.524980950 | 0.270275351 | -0.061464008 |
| H | 1.335419265 | 2.531708136 | 0.026909636 |
| H | -2.543334277 | 4.080446359 | -1.228531392 |
| H | -2.853492079 | 5.377853383 | -3.096403730 |
| H | -1.827194588 | 6.815392378 | -3.198246003 |
| H | -3.569665010 | 6.942762167 | -3.511472531 |
| H | -4.565871276 | 5.159799997 | -1.148438908 |
| H | -5.243891924 | 6.730390478 | -1.601215858 |
| H | -4.682956510 | 6.448601424 | 0.059065276 |
| H | -2.097685979 | 8.727144464 | -1.507401648 |
| H | -3.256105000 | 8.575924816 | -0.170166418 |
| H | -3.834703562 | 8.738470286 | -1.832750711 |
| H | -1.177215736 | 7.829051878 | 0.335716624 |

Table S6. The Cartesian coordinates of optmized S_0_, S_1_ and T_1_ geometry for NBisICz.

|  | **X (Å)** | **Y (Å)** | **Z (Å)** |
| --- | --- | --- | --- |
|  | ***Optimized S_0_ geometry*** | | |
| C | 2.076358241 | 8.195517654 | 1.114084070 |
| C | 1.275745174 | 7.190005579 | 1.975787131 |
| C | 2.282677233 | 6.335170534 | 2.768773188 |
| C | 0.403679117 | 7.966705645 | 2.990914204 |
| C | 0.370600097 | 6.338884512 | 1.063762065 |
| C | 0.467478089 | 4.948445434 | 0.993346063 |
| C | -0.376781981 | 4.193879387 | 0.159031000 |
| C | -0.254516988 | 2.709769279 | 0.100009996 |
| C | 1.041342100 | 2.129160224 | 0.052428993 |
| C | 1.170391090 | 0.735509126 | -0.002436011 |
| C | 2.165805151 | -0.340682965 | -0.066335016 |
| C | 1.449267088 | -1.596541048 | -0.099095019 |
| C | 2.094485118 | -2.826360145 | -0.158238023 |
| C | 3.490800222 | -2.820908159 | -0.187038025 |
| C | 4.486518279 | -3.897280248 | -0.248469029 |
| C | 5.697275354 | -3.221370213 | -0.248581029 |
| N | 5.583420381 | -1.864589110 | -0.195390025 |
| C | 7.008152441 | -3.678513256 | -0.292916032 |
| C | 7.798378542 | -2.442646176 | -0.262937030 |
| C | 6.886678481 | -1.350653092 | -0.203075026 |
| C | 7.313215505 | -0.009767998 | -0.162093023 |
| C | 8.666400631 | 0.241156005 | -0.180961025 |
| C | 9.629018710 | -0.808242083 | -0.240045029 |
| C | 9.197631617 | -2.178511173 | -0.282124031 |
| C | 10.183093695 | -3.198162261 | -0.340513036 |
| C | 11.525764810 | -2.887256249 | -0.356842037 |
| C | 11.952033859 | -1.539462159 | -0.315594034 |
| C | 11.020339788 | -0.526768076 | -0.258529030 |
| C | 7.101286473 | -5.081227362 | -0.347527036 |
| C | 5.913740335 | -5.841497427 | -0.351318037 |
| C | 4.614335275 | -5.291518347 | -0.303510033 |
| C | 4.207087286 | -1.565634075 | -0.154868023 |
| C | 3.563087252 | -0.336369980 | -0.095035018 |
| C | -0.037449002 | 0.058071088 | -0.007597012 |
| N | 0.074297991 | -1.300844009 | -0.060829016 |
| C | -1.339441091 | 0.527958136 | 0.034881991 |
| C | -2.143715160 | -0.702279943 | 0.006946989 |
| C | -1.232367107 | -1.814838035 | -0.051179015 |
| C | -1.696077156 | -3.125219125 | -0.085349017 |
| C | -3.074914260 | -3.333877122 | -0.060595016 |
| C | -3.999229315 | -2.270134038 | -0.001430011 |
| C | -5.461114396 | -2.540007038 | 0.026528991 |
| C | -5.978749495 | -3.605783109 | 0.768924044 |
| C | -7.355064588 | -3.878141114 | 0.808607046 |
| C | -7.872696618 | -5.060158196 | 1.651553108 |
| C | -7.226951570 | -6.373368294 | 1.148905070 |
| C | -7.493616580 | -4.839080182 | 3.135380217 |
| C | -9.402696744 | -5.219885190 | 1.568619100 |
| C | -8.203908606 | -3.041512047 | 0.078388994 |
| C | -7.727416561 | -1.956948972 | -0.681033059 |
| C | -8.725581645 | -1.084733899 | -1.467347116 |
| C | -9.488477699 | -1.962685952 | -2.487810190 |
| C | -9.736436717 | -0.445924841 | -0.485146046 |
| C | -8.031037564 | 0.051591176 | -2.241797174 |
| C | -6.351634455 | -1.724360971 | -0.694613059 |
| C | -3.513788264 | -0.951916945 | 0.031062991 |
| C | -1.438702080 | 1.923104239 | 0.093309995 |
| C | -1.331521043 | 4.864446446 | -0.611354057 |
| C | -1.466438037 | 6.261055556 | -0.569206052 |
| C | -2.530539104 | 6.949363601 | -1.446494114 |
| C | -2.244461089 | 6.645585591 | -2.936511221 |
| C | -3.932224214 | 6.409819610 | -1.074517090 |
| C | -2.545958091 | 8.479192696 | -1.265798102 |
| C | -0.605770967 | 6.971436589 | 0.272289008 |
| H | 2.722672293 | 8.813248694 | 1.749845115 |
| H | 1.418744202 | 8.868791720 | 0.554507027 |
| H | 2.711666279 | 7.672003607 | 0.390401017 |
| H | 2.903939287 | 6.984342534 | 3.396198231 |
| H | 2.953522275 | 5.776027459 | 2.106526139 |
| H | 1.780348188 | 5.619972453 | 3.429831234 |
| H | 1.034431167 | 8.581961670 | 3.644469252 |
| H | -0.169250932 | 7.277658604 | 3.621904250 |
| H | -0.308245927 | 8.632699713 | 2.492337169 |
| H | 1.185657134 | 4.418360387 | 1.608131104 |
| H | 1.906086169 | 2.785640263 | 0.036271991 |
| H | 1.538725068 | -3.758075204 | -0.181812024 |
| H | 6.594965500 | 0.802391066 | -0.117145020 |
| H | 9.023461657 | 1.267367076 | -0.150386022 |
| H | 9.868536662 | -4.236292329 | -0.372498038 |
| H | 12.263769848 | -3.683770315 | -0.401773040 |
| H | 13.013213899 | -1.306992154 | -0.329053035 |
| H | 11.340079847 | 0.512390995 | -0.226552027 |
| H | 8.054186529 | -5.599782383 | -0.386901039 |
| H | 6.005923382 | -6.923372478 | -0.393898039 |
| H | 3.752705203 | -5.953159383 | -0.310318034 |
| H | 4.117929301 | 0.595803083 | -0.071702016 |
| H | -1.011986114 | -3.966101189 | -0.140187022 |
| H | -3.450184296 | -4.351433192 | -0.114288020 |
| H | -5.291910447 | -4.216217160 | 1.347821084 |
| H | -6.134919491 | -6.346035284 | 1.224439074 |
| H | -7.484823612 | -6.560359319 | 0.100150996 |
| H | -7.580562625 | -7.224881376 | 1.743276115 |
| H | -6.410159501 | -4.761112190 | 3.272657225 |
| H | -7.848638633 | -5.676260243 | 3.749080260 |
| H | -7.945773615 | -3.918514110 | 3.521783243 |
| H | -9.739067786 | -5.406263216 | 0.542279027 |
| H | -9.927126744 | -4.334541118 | 1.945746131 |
| H | -9.717487779 | -6.073808250 | 2.179127146 |
| H | -9.270653685 | -3.230934049 | 0.093473995 |
| H | -10.045500731 | -2.768095005 | -1.997895157 |
| H | -8.796644646 | -2.422106992 | -3.203022241 |
| H | -10.207311757 | -1.355491900 | -3.051798232 |
| H | -10.301363752 | -1.202258890 | 0.069610994 |
| H | -10.457728750 | 0.175307212 | -1.030379088 |
| H | -9.223711641 | 0.190241199 | 0.245401006 |
| H | -7.321635509 | -0.333254860 | -2.983125228 |
| H | -7.491577554 | 0.731302220 | -1.572341127 |
| H | -8.780693603 | 0.643673224 | -2.778896211 |
| H | -5.939872430 | -0.917842915 | -1.290059106 |
| H | -4.212996306 | -0.123603879 | 0.101313996 |
| H | -2.398536146 | 2.426372282 | 0.158995000 |
| H | -1.962887092 | 4.281123411 | -1.275225104 |
| H | -2.263079100 | 5.570869514 | -3.144808240 |
| H | -1.259467015 | 7.025352607 | -3.231135242 |
| H | -2.998091138 | 7.122711643 | -3.575233267 |
| H | -4.005955231 | 5.327776493 | -1.225242101 |
| H | -4.701823261 | 6.885640605 | -1.694794134 |
| H | -4.165823229 | 6.617587616 | -0.023985013 |
| H | -1.589258013 | 8.933629744 | -1.547426123 |
| H | -2.770716105 | 8.766275746 | -0.232221028 |
| H | -3.319896143 | 8.917880741 | -1.905792148 |
| H | -0.689564962 | 8.050583678 | 0.321784012 |
| ***Optimized S_1_ geometry*** | | | |
| C | 2.058120512 | 8.230086845 | 1.076279636 |
| C | 1.270272735 | 7.222062343 | 1.946762998 |
| C | 2.288437988 | 6.371338846 | 2.729723812 |
| C | 0.405614811 | 7.996021218 | 2.970311629 |
| C | 0.359022589 | 6.366921328 | 1.044703180 |
| C | 0.461298015 | 4.977139070 | 0.973066943 |
| C | -0.390258012 | 4.218763719 | 0.149255086 |
| C | -0.264735437 | 2.735417971 | 0.091267698 |
| C | 1.041074326 | 2.164648703 | 0.046582618 |
| C | 1.178274397 | 0.765039418 | -0.005587838 |
| C | 2.167593966 | -0.305184697 | -0.066600884 |
| C | 1.449061783 | -1.585436660 | -0.099365386 |
| C | 2.079498808 | -2.826906955 | -0.156612891 |
| C | 3.476402616 | -2.848000066 | -0.185310765 |
| C | 4.457628297 | -3.908333592 | -0.243308441 |
| C | 5.669272458 | -3.239640486 | -0.243803507 |
| N | 5.545430853 | -1.852533283 | -0.191451190 |
| C | 6.977903770 | -3.678391461 | -0.286009734 |
| C | 7.753002122 | -2.443612501 | -0.257229437 |
| C | 6.837704767 | -1.330254754 | -0.198794793 |
| C | 7.262707442 | 0.005384304 | -0.159641756 |
| C | 8.618747849 | 0.259830696 | -0.177930629 |
| C | 9.580251798 | -0.796933846 | -0.235063069 |
| C | 9.150432129 | -2.172260875 | -0.275594171 |
| C | 10.132255902 | -3.193909089 | -0.331808199 |
| C | 11.480775589 | -2.888822143 | -0.348036469 |
| C | 11.902000256 | -1.544543859 | -0.308533505 |
| C | 10.968167779 | -0.524124527 | -0.253279502 |
| C | 7.087916333 | -5.097041226 | -0.339353865 |
| C | 5.897917300 | -5.853283792 | -0.342537668 |
| C | 4.594690517 | -5.323086236 | -0.297052956 |
| C | 4.197676572 | -1.561305241 | -0.153527960 |
| C | 3.559528101 | -0.319255152 | -0.095382030 |
| C | -0.030881257 | 0.093329226 | -0.010395770 |
| N | 0.092100735 | -1.287132656 | -0.062740013 |
| C | -1.328707508 | 0.546546741 | 0.028229036 |
| C | -2.121411138 | -0.689398716 | -0.000249592 |
| C | -1.200885951 | -1.804665714 | -0.055366130 |
| C | -1.653963426 | -3.122834530 | -0.087123630 |
| C | -3.027178249 | -3.345474629 | -0.060722304 |
| C | -3.959589583 | -2.282270332 | -0.005295283 |
| C | -5.414101019 | -2.560028713 | 0.026303567 |
| C | -5.916381511 | -3.672607786 | 0.712761696 |
| C | -7.289728449 | -3.952328917 | 0.759086306 |
| C | -7.790981279 | -5.181326658 | 1.542529688 |
| C | -7.148240423 | -6.461685913 | 0.957878095 |
| C | -7.390549257 | -5.040613103 | 3.030556745 |
| C | -9.321565299 | -5.341542298 | 1.472557930 |
| C | -8.152127977 | -3.079668568 | 0.088097686 |
| C | -7.693068248 | -1.950652720 | -0.616820072 |
| C | -8.707553846 | -1.044225719 | -1.340471256 |
| C | -9.478175200 | -1.871619511 | -2.396839916 |
| C | -9.708429058 | -0.468296000 | -0.310251711 |
| C | -8.030865211 | 0.137660112 | -2.060509192 |
| C | -6.320435646 | -1.707463743 | -0.633785759 |
| C | -3.484680239 | -0.953708729 | 0.022341822 |
| C | -1.445529932 | 1.952864977 | 0.084284795 |
| C | -1.355768442 | 4.885445576 | -0.611586715 |
| C | -1.494329944 | 6.281439940 | -0.569986721 |
| C | -2.568591049 | 6.965667393 | -1.438033433 |
| C | -2.294794884 | 6.661670780 | -2.930299019 |
| C | -3.965091752 | 6.421746578 | -1.053193575 |
| C | -2.587497362 | 8.495546972 | -1.258175283 |
| C | -0.627786974 | 6.995369212 | 0.262670291 |
| H | 2.708635787 | 8.850516530 | 1.705056913 |
| H | 1.392321789 | 8.900743654 | 0.523223757 |
| H | 2.687910047 | 7.708584201 | 0.346382684 |
| H | 2.913449385 | 7.023089633 | 3.350690692 |
| H | 2.954746680 | 5.814654594 | 2.060918622 |
| H | 1.795562823 | 5.654339552 | 3.395924255 |
| H | 1.040693858 | 8.614102149 | 3.616941515 |
| H | -0.158083126 | 7.305170053 | 3.607632700 |
| H | -0.313958848 | 8.659025012 | 2.478709558 |
| H | 1.189120726 | 4.449770914 | 1.578484012 |
| H | 1.900558754 | 2.826305545 | 0.023621073 |
| H | 1.506897453 | -3.748280377 | -0.178368984 |
| H | 6.544961478 | 0.818355894 | -0.116338181 |
| H | 8.978052798 | 1.284563781 | -0.148732283 |
| H | 9.811475903 | -4.229769796 | -0.362273344 |
| H | 12.216933269 | -3.686687039 | -0.391315467 |
| H | 12.962767009 | -1.308822684 | -0.321503109 |
| H | 11.294981087 | 0.512781692 | -0.222870503 |
| H | 8.042952995 | -5.608888412 | -0.377245383 |
| H | 5.996130692 | -6.935822061 | -0.383592538 |
| H | 3.737567834 | -5.988759676 | -0.303746134 |
| H | 4.129761731 | 0.603497579 | -0.073779912 |
| H | -0.960671982 | -3.955759991 | -0.141822756 |
| H | -3.394160708 | -4.364937372 | -0.116993623 |
| H | -5.220187598 | -4.311170270 | 1.247581297 |
| H | -6.055316998 | -6.435656743 | 1.020668389 |
| H | -7.419810695 | -6.591011329 | -0.096046209 |
| H | -7.491562314 | -7.345921305 | 1.508486276 |
| H | -6.305375526 | -4.967306116 | 3.156875730 |
| H | -7.734451496 | -5.911688450 | 3.601544420 |
| H | -7.839490481 | -4.144668416 | 3.474232915 |
| H | -9.672487975 | -5.472893352 | 0.442573987 |
| H | -9.843326605 | -4.480720248 | 1.905977100 |
| H | -9.624535732 | -6.228975984 | 2.039353098 |
| H | -9.217457190 | -3.275671054 | 0.107522602 |
| H | -10.023941150 | -2.706148521 | -1.944369762 |
| H | -8.793479002 | -2.286508894 | -3.145304476 |
| H | -10.208190720 | -1.239620931 | -2.917102619 |
| H | -10.260427553 | -1.257864751 | 0.210212701 |
| H | -10.441747385 | 0.175508446 | -0.811034123 |
| H | -9.190302777 | 0.131733362 | 0.446530611 |
| H | -7.329510778 | -0.200509874 | -2.831705084 |
| H | -7.487437536 | 0.784430335 | -1.362201899 |
| H | -8.791784471 | 0.752333343 | -2.554260686 |
| H | -5.923677648 | -0.866418094 | -1.189583608 |
| H | -4.192283679 | -0.133529799 | 0.094045551 |
| H | -2.408458579 | 2.447241705 | 0.154687335 |
| H | -1.991465605 | 4.298324667 | -1.267681201 |
| H | -2.311273225 | 5.586665032 | -3.137330299 |
| H | -1.313837030 | 7.044641858 | -3.234014882 |
| H | -3.055768352 | 7.135565675 | -3.562715579 |
| H | -4.036335292 | 5.339310546 | -1.202471127 |
| H | -4.741799030 | 6.894522158 | -1.666939958 |
| H | -4.190010879 | 6.629524902 | -0.000757315 |
| H | -1.635008942 | 8.952984508 | -1.548998975 |
| H | -2.803546065 | 8.782596683 | -0.222725007 |
| H | -3.368799254 | 8.931248923 | -1.891230587 |
| H | -0.714448951 | 8.074324845 | 0.311833596 |
| ***Optimized T_1_ geometry*** | | | |
| C | 2.003985040 | 8.228636265 | 1.107741663 |
| C | 1.213580130 | 7.216282775 | 1.970821459 |
| C | 2.229272924 | 6.370196459 | 2.762011536 |
| C | 0.336758780 | 7.985490696 | 2.987574714 |
| C | 0.314114225 | 6.357342108 | 1.060481293 |
| C | 0.422499712 | 4.967749952 | 0.990369160 |
| C | -0.416655059 | 4.205574802 | 0.157709431 |
| C | -0.282264027 | 2.722544835 | 0.099203835 |
| C | 1.019290454 | 2.152667040 | 0.052325081 |
| C | 1.160715185 | 0.760688522 | -0.002095394 |
| C | 2.166241272 | -0.306648804 | -0.065164345 |
| C | 1.459635676 | -1.572124825 | -0.097803415 |
| C | 2.111893615 | -2.795456199 | -0.156088692 |
| C | 3.511720472 | -2.781627787 | -0.184541892 |
| C | 4.511867510 | -3.843272344 | -0.244847496 |
| C | 5.713632434 | -3.166889505 | -0.245103025 |
| N | 5.588807935 | -1.801432377 | -0.191994136 |
| C | 7.034869648 | -3.625958272 | -0.289922075 |
| C | 7.823725392 | -2.426699706 | -0.262172866 |
| C | 6.878086177 | -1.261992048 | -0.199309958 |
| C | 7.315415454 | 0.029190126 | -0.161225272 |
| C | 8.729071781 | 0.261514562 | -0.182594462 |
| C | 9.682203140 | -0.801674300 | -0.241658592 |
| C | 9.239262097 | -2.173998963 | -0.282619947 |
| C | 10.195568837 | -3.195311138 | -0.340101635 |
| C | 11.588416010 | -2.907792957 | -0.358933466 |
| C | 12.015277208 | -1.596511573 | -0.319962803 |
| C | 11.066017257 | -0.544999098 | -0.261447047 |
| C | 7.133570254 | -5.039170746 | -0.344396828 |
| C | 5.944007315 | -5.800398029 | -0.347559391 |
| C | 4.648697051 | -5.255872608 | -0.300100579 |
| C | 4.216287969 | -1.516571793 | -0.152471401 |
| C | 3.560802180 | -0.290741347 | -0.093282531 |
| C | -0.041304744 | 0.072729074 | -0.007500743 |
| N | 0.081836879 | -1.285829610 | -0.060098846 |
| C | -1.347067535 | 0.531356761 | 0.034460944 |
| C | -2.140613001 | -0.705274970 | 0.006891453 |
| C | -1.219432158 | -1.810273262 | -0.050566459 |
| C | -1.672445031 | -3.124806213 | -0.084139672 |
| C | -3.049222583 | -3.345162086 | -0.059439216 |
| C | -3.982717942 | -2.289083214 | -0.001065654 |
| C | -5.442184561 | -2.571490475 | 0.026762575 |
| C | -5.950873602 | -3.642141057 | 0.768430227 |
| C | -7.324779510 | -3.926359787 | 0.807924439 |
| C | -7.832145819 | -5.113396084 | 1.650064723 |
| C | -7.174884339 | -6.420596676 | 1.146701756 |
| C | -7.455249589 | -4.889988271 | 3.134096470 |
| C | -9.360682187 | -5.286496630 | 1.566862533 |
| C | -8.180902678 | -3.096646760 | 0.078310396 |
| C | -7.713628107 | -2.007517245 | -0.680314335 |
| C | -8.719202942 | -1.143281430 | -1.466019670 |
| C | -9.474574519 | -2.026889265 | -2.487203207 |
| C | -9.735491765 | -0.513831973 | -0.483400151 |
| C | -8.034382599 | -0.000499614 | -2.239623536 |
| C | -6.339895875 | -1.763169359 | -0.693808212 |
| C | -3.508591187 | -0.966862435 | 0.030799867 |
| C | -1.458977898 | 1.926384921 | 0.092419833 |
| C | -1.378041402 | 4.868182009 | -0.611403604 |
| C | -1.524688885 | 6.263597406 | -0.569396032 |
| C | -2.596013898 | 6.942595424 | -1.445163487 |
| C | -2.309917790 | 6.640727161 | -2.935563269 |
| C | -3.992489761 | 6.391424986 | -1.070680782 |
| C | -2.624068960 | 8.472314503 | -1.265037193 |
| C | -0.668759893 | 6.981592823 | 0.270466125 |
| H | 2.646091492 | 8.852030454 | 1.742287474 |
| H | 1.339581630 | 8.896115787 | 0.549240449 |
| H | 2.642480074 | 7.710549724 | 0.382946333 |
| H | 2.846238588 | 7.024746963 | 3.388132596 |
| H | 2.903440543 | 5.816571538 | 2.098510035 |
| H | 1.734285436 | 5.650851851 | 3.424089153 |
| H | 0.963349830 | 8.606010183 | 3.640199501 |
| H | -0.229256735 | 7.291492197 | 3.619375646 |
| H | -0.381653292 | 8.645443413 | 2.490265747 |
| H | 1.146085392 | 4.443875658 | 1.604117331 |
| H | 1.878282445 | 2.816686817 | 0.036051679 |
| H | 1.562015704 | -3.730718047 | -0.179477522 |
| H | 6.627258225 | 0.866380205 | -0.116219426 |
| H | 9.095051719 | 1.283316340 | -0.153036334 |
| H | 9.870803922 | -4.229803426 | -0.371035468 |
| H | 12.301738686 | -3.725322889 | -0.403893745 |
| H | 13.075317559 | -1.358959371 | -0.333658590 |
| H | 11.409009629 | 0.486075636 | -0.231001695 |
| H | 8.088028525 | -5.552877223 | -0.383683490 |
| H | 6.038536787 | -6.882211748 | -0.389728428 |
| H | 3.787118669 | -5.916883912 | -0.306541915 |
| H | 4.109177281 | 0.645469236 | -0.070230856 |
| H | -0.981100229 | -3.959763466 | -0.138526085 |
| H | -3.415887673 | -4.365904120 | -0.112685731 |
| H | -5.258790227 | -4.247022245 | 1.346908645 |
| H | -6.083154102 | -6.383685398 | 1.222385787 |
| H | -7.430924105 | -6.609116864 | 0.097767071 |
| H | -7.521106121 | -7.275601216 | 1.740449113 |
| H | -6.372546111 | -4.802525131 | 3.271522489 |
| H | -7.802975135 | -5.730646911 | 3.747255884 |
| H | -7.915523833 | -3.973657009 | 3.520993070 |
| H | -9.695316152 | -5.474974394 | 0.540333078 |
| H | -9.892878287 | -4.406058328 | 1.944597504 |
| H | -9.668030662 | -6.143633188 | 2.176685464 |
| H | -9.245992385 | -3.295234546 | 0.093266822 |
| H | -10.024805085 | -2.837328715 | -1.997908323 |
| H | -8.778767398 | -2.479952193 | -3.202617912 |
| H | -10.198455340 | -1.425408793 | -3.050893973 |
| H | -10.293958022 | -1.275376120 | 0.070789383 |
| H | -10.462045090 | 0.101727365 | -1.028123364 |
| H | -9.228171865 | 0.126060126 | 0.247669935 |
| H | -7.321842364 | -0.378773193 | -2.981313415 |
| H | -7.500513665 | 0.683126166 | -1.569686959 |
| H | -8.789083497 | 0.585707614 | -2.776127609 |
| H | -5.935044905 | -0.952786277 | -1.288728130 |
| H | -4.214866133 | -0.144488417 | 0.100585113 |
| H | -2.423398047 | 2.420831405 | 0.157974141 |
| H | -2.005426487 | 4.279305544 | -1.274124410 |
| H | -2.319651620 | 5.565810527 | -3.143368243 |
| H | -1.328672567 | 7.028765006 | -3.231954769 |
| H | -3.068662248 | 7.111157004 | -3.573234210 |
| H | -4.057245752 | 5.308735320 | -1.220771902 |
| H | -4.767164226 | 6.860443460 | -1.689859794 |
| H | -4.226059613 | 6.597626362 | -0.019828596 |
| H | -1.671779205 | 8.934721701 | -1.548656455 |
| H | -2.849200647 | 8.757868548 | -0.231113079 |
| H | -3.402952639 | 8.904190344 | -1.903690922 |
| H | -0.761516452 | 8.060028222 | 0.319765690 |

Table S7. The Cartesian coordinates of optmized S_0_, S_1_ and T_1_ geometry for NBisICz-PCz.

|  | **X (Å)** | **Y (Å)** | **Z (Å)** |
| --- | --- | --- | --- |
| ***Optimized S_0_ geometry*** | | | |
| C | 2.524343682 | 9.612144729 | 1.386720432 |
| C | 2.825031773 | 8.347507943 | 2.226194340 |
| C | 3.937070223 | 8.667659544 | 3.253379002 |
| C | 1.548286756 | 7.979026743 | 3.005671952 |
| C | 3.294363674 | 7.212612719 | 1.294899311 |
| C | 2.623286170 | 5.992804654 | 1.197776992 |
| C | 3.076254147 | 4.969687281 | 0.345502184 |
| C | 2.340765802 | 3.676642128 | 0.259176979 |
| C | 0.921471849 | 3.695714814 | 0.206292872 |
| C | 0.217636752 | 2.488244454 | 0.128770061 |
| C | -1.133700522 | 1.949215480 | -0.030571151 |
| C | -1.026077279 | 0.510046511 | -0.122118103 |
| C | -2.134271611 | -0.296829084 | -0.358823021 |
| C | -3.376460708 | 0.328480971 | -0.478588003 |
| C | -4.722488389 | -0.196626978 | -0.738162532 |
| C | -5.520524755 | 0.937217994 | -0.738141513 |
| N | -4.844472154 | 2.099742938 | -0.515767876 |
| C | -6.883653651 | 1.105073044 | -0.944672725 |
| C | -7.066470155 | 2.556255102 | -0.830293508 |
| C | -5.789755005 | 3.131157414 | -0.569745501 |
| C | -5.603236641 | 4.516849073 | -0.405300807 |
| C | -6.704787084 | 5.336155115 | -0.503351879 |
| C | -8.008657600 | 4.821662995 | -0.761840377 |
| C | -8.203739216 | 3.407575156 | -0.929096737 |
| C | -9.515724506 | 2.929765227 | -1.183749598 |
| C | -10.582947274 | 3.797649947 | -1.270673439 |
| C | -10.392637196 | 5.189403119 | -1.107081287 |
| C | -9.131722908 | 5.684610978 | -0.858300219 |
| C | -7.557347195 | -0.105998761 | -1.189574528 |
| C | -6.815130436 | -1.304597230 | -1.203877691 |
| C | -5.421720642 | -1.384412177 | -0.987085509 |
| C | -3.485139047 | 1.762576045 | -0.354053224 |
| C | -2.380016990 | 2.573648153 | -0.137755167 |
| C | 1.017901903 | 1.358008963 | 0.131822779 |
| N | 0.343477732 | 0.173313634 | 0.022257899 |
| C | 2.396584652 | 1.244217338 | 0.141613310 |
| C | 2.617971267 | -0.199731783 | 0.027311004 |
| C | 1.328332168 | -0.841919548 | -0.022552261 |
| C | 1.223803125 | -2.238389849 | -0.074249292 |
| C | -0.066970566 | -2.979719427 | 0.029841880 |
| C | -0.757290569 | -3.002157426 | 1.246969335 |
| C | -1.943118263 | -3.732739965 | 1.361630118 |
| C | -2.437800098 | -4.448193437 | 0.240537938 |
| N | -3.633868271 | -5.079385624 | 0.595620549 |
| C | -4.451365796 | -5.860483778 | -0.266581746 |
| C | -5.796843440 | -5.518563307 | -0.455977222 |
| C | -6.597892327 | -6.289994990 | -1.297819083 |
| C | -6.061300299 | -7.390989532 | -1.968298151 |
| C | -4.718248171 | -7.725421404 | -1.784539330 |
| C | -3.914919737 | -6.971514537 | -0.929061965 |
| C | -3.908719102 | -4.780440389 | 1.936813391 |
| C | -2.879580777 | -3.948568060 | 2.444787984 |
| C | -2.927299510 | -3.525431057 | 3.778236799 |
| C | -3.985647515 | -3.938619310 | 4.581429208 |
| C | -4.990936526 | -4.774712188 | 4.065298533 |
| C | -4.967700125 | -5.208904095 | 2.741827768 |
| C | -1.764876797 | -4.430559441 | -0.984768777 |
| C | -0.580102309 | -3.701725299 | -1.069172352 |
| C | 2.423615332 | -2.958974700 | -0.165748542 |
| C | 3.697101015 | -2.359994003 | -0.160291523 |
| C | 4.927170239 | -3.190271233 | -0.251220898 |
| C | 5.016134544 | -4.419355706 | 0.409431649 |
| C | 6.169102595 | -5.216152601 | 0.333922282 |
| C | 6.216392837 | -6.558669445 | 1.089706435 |
| C | 6.035631075 | -6.303064874 | 2.605044128 |
| C | 5.075120075 | -7.473867315 | 0.585645938 |
| C | 7.549225912 | -7.304228858 | 0.887221677 |
| C | 7.240207037 | -4.738735131 | -0.426701745 |
| C | 7.195703812 | -3.507347668 | -1.106199540 |
| C | 8.414678369 | -3.052213577 | -1.932324185 |
| C | 9.648752153 | -2.932319412 | -1.006463065 |
| C | 8.705730301 | -4.091214561 | -3.041316553 |
| C | 8.191155751 | -1.685285433 | -2.606913537 |
| C | 6.028687952 | -2.748931116 | -1.006481147 |
| C | 3.778314514 | -0.964667767 | -0.042844399 |
| C | 3.078906569 | 2.464214451 | 0.216583145 |
| C | 4.227476501 | 5.192855345 | -0.415918555 |
| C | 4.936071825 | 6.402572369 | -0.346773107 |
| C | 6.194212532 | 6.597919685 | -1.215510214 |
| C | 7.237943481 | 5.511590073 | -0.862485784 |
| C | 5.812398976 | 6.473805924 | -2.709859525 |
| C | 6.850759887 | 7.975392936 | -1.003722350 |
| C | 4.449701735 | 7.392179810 | 0.512199416 |
| H | 2.193280526 | 10.431888443 | 2.036309022 |
| H | 1.731828815 | 9.416925897 | 0.655146157 |
| H | 3.406508043 | 9.958042761 | 0.838074964 |
| H | 3.619236631 | 9.478576846 | 3.920448957 |
| H | 4.865318716 | 8.982562870 | 2.765354968 |
| H | 4.164783068 | 7.790249427 | 3.869422405 |
| H | 1.253232497 | 8.816892132 | 3.647580460 |
| H | 1.700912804 | 7.106635079 | 3.651007427 |
| H | 0.708294794 | 7.765369206 | 2.334866582 |
| H | 1.745797274 | 5.801181635 | 1.804425764 |
| H | 0.412984864 | 4.655094746 | 0.191289670 |
| H | -2.042332107 | -1.369272442 | -0.461142181 |
| H | -4.618077686 | 4.926136872 | -0.206448804 |
| H | -6.589017972 | 6.410062291 | -0.381198416 |
| H | -9.674470273 | 1.863678243 | -1.310291304 |
| H | -11.579348558 | 3.410226202 | -1.466377484 |
| H | -11.241594193 | 5.863693767 | -1.177874413 |
| H | -8.977036196 | 6.753733598 | -0.730850911 |
| H | -8.626467593 | -0.149794230 | -1.373246363 |
| H | -7.351353733 | -2.228956688 | -1.402652838 |
| H | -4.926290074 | -2.350334301 | -1.023851448 |
| H | -2.467766628 | 3.653102678 | -0.070155736 |
| H | -0.366835302 | -2.450920111 | 2.098053635 |
| H | -6.202152572 | -4.647837492 | 0.050557441 |
| H | -7.641177180 | -6.021103466 | -1.438670927 |
| H | -6.685996158 | -7.984981681 | -2.629257691 |
| H | -4.295058320 | -8.584815205 | -2.297226681 |
| H | -2.877366948 | -7.243205937 | -0.761353354 |
| H | -2.146194694 | -2.885531497 | 4.180041301 |
| H | -4.035845486 | -3.615813754 | 5.617270263 |
| H | -5.804925564 | -5.092922137 | 4.710959564 |
| H | -5.743070935 | -5.860914052 | 2.353669768 |
| H | -2.149301563 | -4.964086278 | -1.847731878 |
| H | -0.040662697 | -3.673671041 | -2.011655088 |
| H | 2.353720347 | -4.040363151 | -0.237796457 |
| H | 4.175838014 | -4.744527574 | 1.015550176 |
| H | 5.081614074 | -5.813148767 | 2.825365070 |
| H | 6.836589790 | -5.662842743 | 2.992120759 |
| H | 6.059955236 | -7.250397773 | 3.157794652 |
| H | 4.090247510 | -7.021825217 | 0.742273683 |
| H | 5.090284977 | -8.432437187 | 1.118925401 |
| H | 5.182087397 | -7.679087438 | -0.485736664 |
| H | 8.402305147 | -6.724469567 | 1.257697975 |
| H | 7.728394110 | -7.541689052 | -0.167669133 |
| H | 7.529438652 | -8.250515733 | 1.439643555 |
| H | 8.141669265 | -5.335531797 | -0.499515530 |
| H | 9.893014708 | -3.885038034 | -0.525226709 |
| H | 9.474293791 | -2.193978325 | -0.215436795 |
| H | 10.527559138 | -2.614216521 | -1.580932012 |
| H | 8.920039573 | -5.081807578 | -2.626888612 |
| H | 9.575224985 | -3.782915075 | -3.635011918 |
| H | 7.849693389 | -4.190579273 | -3.718494571 |
| H | 8.005089146 | -0.893472117 | -1.872362661 |
| H | 7.348157555 | -1.707833209 | -3.306743908 |
| H | 9.084776948 | -1.404302182 | -3.175749493 |
| H | 5.941583683 | -1.808010537 | -1.537193068 |
| H | 4.748858736 | -0.480516397 | 0.015836129 |
| H | 4.162856683 | 2.510250025 | 0.260353851 |
| H | 4.558274111 | 4.411986436 | -1.094437941 |
| H | 6.850465294 | 4.502254296 | -1.035754020 |
| H | 7.533431506 | 5.579864205 | 0.190786687 |
| H | 8.138523782 | 5.632667045 | -1.477133075 |
| H | 5.377948846 | 5.495568597 | -2.940170180 |
| H | 6.699106207 | 6.603010794 | -3.342861334 |
| H | 5.079486951 | 7.238639508 | -2.991138638 |
| H | 7.170768769 | 8.120171016 | 0.034488207 |
| H | 6.175115530 | 8.795665880 | -1.271625354 |
| H | 7.740248421 | 8.060948514 | -1.638193198 |
| H | 4.978671552 | 8.335243255 | 0.582323489 |
| ***Optimized S_1_ geometry*** | | | |
| C | 2.629835823 | 9.550964113 | 1.433141443 |
| C | 2.895080648 | 8.278562656 | 2.272857153 |
| C | 3.987124538 | 8.579477659 | 3.326960040 |
| C | 1.596114653 | 7.922420864 | 3.020683562 |
| C | 3.371482087 | 7.142279665 | 1.346933040 |
| C | 2.687230133 | 5.932054365 | 1.227539079 |
| C | 3.148210574 | 4.906670584 | 0.381963659 |
| C | 2.400979659 | 3.622547825 | 0.274032727 |
| C | 0.978088491 | 3.666364728 | 0.207903407 |
| C | 0.256671419 | 2.463026528 | 0.108863309 |
| C | -1.088502959 | 1.938248668 | -0.073577926 |
| C | -0.996912098 | 0.476198721 | -0.187638269 |
| C | -2.096648506 | -0.331267982 | -0.481424312 |
| C | -3.341074695 | 0.283556343 | -0.629605867 |
| C | -4.663899975 | -0.216586847 | -0.937985227 |
| C | -5.456698464 | 0.916951421 | -0.929065156 |
| N | -4.758553523 | 2.088100420 | -0.649311714 |
| C | -6.805249479 | 1.116229809 | -1.157745939 |
| C | -6.970093961 | 2.556059931 | -1.000775886 |
| C | -5.686573061 | 3.131631165 | -0.686949102 |
| C | -5.497340706 | 4.502831200 | -0.472008226 |
| C | -6.593868516 | 5.337142295 | -0.569199247 |
| C | -7.894596989 | 4.833162138 | -0.879069960 |
| C | -8.096495202 | 3.422713409 | -1.099436168 |
| C | -9.401166469 | 2.958897467 | -1.403278553 |
| C | -10.469100398 | 3.833337793 | -1.490114227 |
| C | -10.273141565 | 5.212360390 | -1.275390699 |
| C | -9.011821557 | 5.696914825 | -0.976595592 |
| C | -7.500848951 | -0.088502771 | -1.458153355 |
| C | -6.762346751 | -1.290306883 | -1.489713735 |
| C | -5.381336533 | -1.406095601 | -1.243053831 |
| C | -3.436399806 | 1.742957381 | -0.464602116 |
| C | -2.329198914 | 2.554836008 | -0.202645442 |
| C | 1.051828631 | 1.331681500 | 0.111126449 |
| N | 0.350400883 | 0.137554743 | -0.020860252 |
| C | 2.418592387 | 1.192986594 | 0.137136288 |
| C | 2.616932042 | -0.252007317 | 0.015032141 |
| C | 1.313049175 | -0.884055821 | -0.036901865 |
| C | 1.185714037 | -2.285001712 | -0.070879454 |
| C | -0.108533970 | -2.997268125 | 0.095617435 |
| C | -0.831477637 | -2.842768281 | 1.285448115 |
| C | -2.030552315 | -3.534485301 | 1.463864109 |
| C | -2.510706684 | -4.383478282 | 0.431014159 |
| N | -3.730508329 | -4.931241945 | 0.824855873 |
| C | -4.561692807 | -5.771442561 | 0.030563515 |
| C | -5.855789764 | -5.354479412 | -0.305514103 |
| C | -6.669832333 | -6.182065638 | -1.078860471 |
| C | -6.194341598 | -7.414127913 | -1.533012869 |
| C | -4.900445491 | -7.822603271 | -1.203809734 |
| C | -4.085662020 | -7.009787228 | -0.415295728 |
| C | -4.041178290 | -4.447402693 | 2.102388523 |
| C | -3.004483086 | -3.583234734 | 2.534136171 |
| C | -3.084058475 | -2.985681651 | 3.797427544 |
| C | -4.182849073 | -3.259569508 | 4.605911954 |
| C | -5.197840290 | -4.126567342 | 4.164848744 |
| C | -5.142886476 | -4.733574293 | 2.912264351 |
| C | -1.801065800 | -4.548983155 | -0.762542437 |
| C | -0.599093126 | -3.861053869 | -0.908508276 |
| C | 2.371590806 | -3.022215916 | -0.185497597 |
| C | 3.652979512 | -2.434647599 | -0.199357262 |
| C | 4.869485574 | -3.274144208 | -0.305901002 |
| C | 4.930872453 | -4.535220758 | 0.299334924 |
| C | 6.075067533 | -5.341219500 | 0.209100507 |
| C | 6.095429798 | -6.715131282 | 0.907288808 |
| C | 5.894778591 | -6.521431506 | 2.429340157 |
| C | 4.951364048 | -7.593920593 | 0.347706892 |
| C | 7.422775939 | -7.466930754 | 0.692138896 |
| C | 7.163674195 | -4.844270527 | -0.514315777 |
| C | 7.146979023 | -3.583590637 | -1.140600417 |
| C | 8.383833950 | -3.111675754 | -1.929409926 |
| C | 9.605213459 | -3.048634011 | -0.981234561 |
| C | 8.676307094 | -4.107928275 | -3.076615623 |
| C | 8.188368945 | -1.714647891 | -2.548672934 |
| C | 5.990991905 | -2.813055254 | -1.022228927 |
| C | 3.758548765 | -1.034445069 | -0.073186404 |
| C | 3.129216381 | 2.409711539 | 0.230274303 |
| C | 4.319410078 | 5.119041686 | -0.352058011 |
| C | 5.039961242 | 6.319998387 | -0.262036610 |
| C | 6.318755403 | 6.504856511 | -1.102419389 |
| C | 7.341653490 | 5.404147406 | -0.733309490 |
| C | 5.967518978 | 6.393203233 | -2.605225884 |
| C | 6.987011069 | 7.873133348 | -0.868855083 |
| C | 4.546420466 | 7.311212728 | 0.591200553 |
| H | 2.293676605 | 10.371406288 | 2.079176840 |
| H | 1.852394379 | 9.369302696 | 0.682214468 |
| H | 3.528875837 | 9.888788487 | 0.907294132 |
| H | 3.664255528 | 9.391202750 | 3.990581172 |
| H | 4.930354198 | 8.884871564 | 2.862108884 |
| H | 4.189354614 | 7.696313427 | 3.943698396 |
| H | 1.296955990 | 8.760740023 | 3.660058396 |
| H | 1.722727946 | 7.044896551 | 3.664702101 |
| H | 0.769200439 | 7.722936546 | 2.329635453 |
| H | 1.792560022 | 5.748779329 | 1.810922843 |
| H | 0.483993515 | 4.632153483 | 0.190819179 |
| H | -1.990004780 | -1.399358873 | -0.607545436 |
| H | -4.515692805 | 4.900297401 | -0.234089784 |
| H | -6.477799572 | 6.405108557 | -0.407669407 |
| H | -9.556115389 | 1.897906041 | -1.568815896 |
| H | -11.460983937 | 3.456530089 | -1.724290791 |
| H | -11.114858729 | 5.896386972 | -1.344668643 |
| H | -8.861659469 | 6.761373330 | -0.810835068 |
| H | -8.564503265 | -0.112784313 | -1.667611431 |
| H | -7.305679439 | -2.201593747 | -1.731901074 |
| H | -4.896404180 | -2.375081053 | -1.301217719 |
| H | -2.428235140 | 3.632379372 | -0.123031625 |
| H | -0.455841594 | -2.184804662 | 2.064260937 |
| H | -6.206390911 | -4.382258058 | 0.027885571 |
| H | -7.673382272 | -5.855814900 | -1.337280672 |
| H | -6.828943973 | -8.052477074 | -2.141168992 |
| H | -4.526474855 | -8.782234515 | -1.549808886 |
| H | -3.087472509 | -7.330931089 | -0.133475466 |
| H | -2.297995326 | -2.318798782 | 4.141523939 |
| H | -4.258801847 | -2.800757804 | 5.587391380 |
| H | -6.045332482 | -4.330237537 | 4.813561635 |
| H | -5.927542971 | -5.404815440 | 2.579374655 |
| H | -2.174907206 | -5.187447688 | -1.556108821 |
| H | -0.034029420 | -3.968363750 | -1.830052532 |
| H | 2.286813906 | -4.102425990 | -0.246275888 |
| H | 4.078458542 | -4.876265266 | 0.878437842 |
| H | 4.942793424 | -6.030786246 | 2.656990056 |
| H | 6.696823495 | -5.907941875 | 2.855380358 |
| H | 5.900373635 | -7.491686512 | 2.940931179 |
| H | 3.969057406 | -7.138365023 | 0.510547286 |
| H | 4.948701990 | -8.574425603 | 0.839291368 |
| H | 5.071289620 | -7.754295167 | -0.729887851 |
| H | 8.276797109 | -6.914351170 | 1.100072922 |
| H | 7.615176585 | -7.661673220 | -0.369156675 |
| H | 7.383607668 | -8.435543299 | 1.202953975 |
| H | 8.058289399 | -5.449424553 | -0.599476928 |
| H | 9.830190997 | -4.024152436 | -0.537702443 |
| H | 9.429533016 | -2.341776520 | -0.162263523 |
| H | 10.496169773 | -2.719033363 | -1.529559728 |
| H | 8.871955194 | -5.117833124 | -2.701372443 |
| H | 9.558238712 | -3.787067111 | -3.644388304 |
| H | 7.829210949 | -4.167002215 | -3.769506470 |
| H | 8.003919770 | -0.951220299 | -1.784226830 |
| H | 7.355320392 | -1.695509851 | -3.260462457 |
| H | 9.093553671 | -1.423359882 | -3.093174045 |
| H | 5.926454612 | -1.849173330 | -1.512715868 |
| H | 4.737854046 | -0.569037748 | -0.019787187 |
| H | 4.211479330 | 2.437513501 | 0.298537831 |
| H | 4.655209672 | 4.336487766 | -1.025932702 |
| H | 6.945696191 | 4.400529548 | -0.920297292 |
| H | 7.615512136 | 5.463326357 | 0.326351688 |
| H | 8.256467760 | 5.517569406 | -1.328099018 |
| H | 5.525968348 | 5.421634386 | -2.849877497 |
| H | 6.869073442 | 6.514684708 | -3.218494489 |
| H | 5.250226424 | 7.168439542 | -2.897943661 |
| H | 7.286047651 | 8.008610238 | 0.176845806 |
| H | 6.327258805 | 8.702902114 | -1.146842692 |
| H | 7.891053989 | 7.951314048 | -1.483399311 |
| H | 5.084723006 | 8.247653308 | 0.677498155 |
| ***Optimized T_1_ geometry*** | | | |
| C | 2.599260337 | 9.600736196 | 1.387734389 |
| C | 2.890253229 | 8.333709488 | 2.227018264 |
| C | 4.003415212 | 8.645775895 | 3.255469537 |
| C | 1.610167743 | 7.973952568 | 3.005098599 |
| C | 3.352562741 | 7.195851347 | 1.295795515 |
| C | 2.672973072 | 5.980859060 | 1.197567983 |
| C | 3.119427476 | 4.954651731 | 0.345490754 |
| C | 2.375168954 | 3.666753657 | 0.258194247 |
| C | 0.955268844 | 3.695986938 | 0.205373865 |
| C | 0.242659992 | 2.494246837 | 0.126869606 |
| C | -1.113165180 | 1.965715290 | -0.033088484 |
| C | -1.016230206 | 0.522433510 | -0.127081622 |
| C | -2.126287406 | -0.276436156 | -0.367725651 |
| C | -3.368330985 | 0.356294230 | -0.488496012 |
| C | -4.711057213 | -0.155825998 | -0.749152374 |
| C | -5.502250254 | 0.973285001 | -0.749904324 |
| N | -4.814904862 | 2.139872333 | -0.522365888 |
| C | -6.875778117 | 1.141459520 | -0.955751094 |
| C | -7.075532316 | 2.559715689 | -0.843200866 |
| C | -5.739582810 | 3.186268224 | -0.567131111 |
| C | -5.586084418 | 4.532134067 | -0.409080068 |
| C | -6.752177182 | 5.358972444 | -0.514385268 |
| C | -8.052022088 | 4.826019050 | -0.774830303 |
| C | -8.234496691 | 3.404775131 | -0.943904212 |
| C | -9.519887756 | 2.909733381 | -1.196367253 |
| C | -10.644342260 | 3.776880163 | -1.290166566 |
| C | -10.473130507 | 5.135968886 | -1.129860749 |
| C | -9.181030268 | 5.661306964 | -0.872639123 |
| C | -7.556654661 | -0.077084501 | -1.202049881 |
| C | -6.811056141 | -1.276626296 | -1.217227986 |
| C | -5.423786920 | -1.357029694 | -1.001385826 |
| C | -3.465249666 | 1.793455004 | -0.359840531 |
| C | -2.352563873 | 2.597721622 | -0.140769612 |
| C | 1.034803925 | 1.358144607 | 0.129161393 |
| N | 0.351949767 | 0.177541879 | 0.019222431 |
| C | 2.412399653 | 1.234493595 | 0.139183062 |
| C | 2.623588854 | -0.210494524 | 0.024633030 |
| C | 1.329035921 | -0.843686365 | -0.024693435 |
| C | 1.215427990 | -2.240016868 | -0.074618673 |
| C | -0.079685286 | -2.972609021 | 0.034508171 |
| C | -0.774217711 | -2.975646048 | 1.249445065 |
| C | -1.964851699 | -3.697458765 | 1.369229082 |
| C | -2.460321040 | -4.423709099 | 0.255608582 |
| N | -3.662274548 | -5.042039136 | 0.614292568 |
| C | -4.483085490 | -5.826526421 | -0.241394201 |
| C | -5.825114417 | -5.475919452 | -0.439494025 |
| C | -6.629650106 | -6.250767345 | -1.274836222 |
| C | -6.099882771 | -7.363963550 | -1.930458882 |
| C | -4.760178139 | -7.706988044 | -1.738299632 |
| C | -3.953560492 | -6.949575695 | -0.889030860 |
| C | -3.939941327 | -4.723996190 | 1.950467873 |
| C | -2.906704503 | -3.893027050 | 2.451608999 |
| C | -2.956306366 | -3.452602345 | 3.779343520 |
| C | -4.020462546 | -3.848055599 | 4.583865710 |
| C | -5.029784999 | -4.683544648 | 4.074748798 |
| C | -5.004831101 | -5.134671460 | 2.756950903 |
| C | -1.783059417 | -4.426081353 | -0.967382515 |
| C | -0.593524139 | -3.705663224 | -1.056841734 |
| C | 2.410006063 | -2.968708815 | -0.167285413 |
| C | 3.687709161 | -2.378295344 | -0.163690201 |
| C | 4.911948630 | -3.216951450 | -0.255568525 |
| C | 4.993236568 | -4.446772691 | 0.404807034 |
| C | 6.140763248 | -5.251289446 | 0.328471557 |
| C | 6.179512729 | -6.594153372 | 1.084152106 |
| C | 6.001395477 | -6.337472931 | 2.599619506 |
| C | 5.031857252 | -7.501710981 | 0.580776307 |
| C | 7.507188177 | -7.348637678 | 0.880834444 |
| C | 7.214668414 | -4.781038306 | -0.432676088 |
| C | 7.177910703 | -3.549266360 | -1.111936515 |
| C | 8.399496539 | -3.102029556 | -1.938544944 |
| C | 9.634503896 | -2.989498633 | -1.012992980 |
| C | 8.683916081 | -4.143117318 | -3.047291245 |
| C | 8.184346034 | -1.733961278 | -2.613537312 |
| C | 6.016052311 | -2.783080829 | -1.011463679 |
| C | 3.778550479 | -0.983662791 | -0.046354329 |
| C | 3.104307807 | 2.449919395 | 0.214917010 |
| C | 4.272947474 | 5.170226143 | -0.414725550 |
| C | 4.990069733 | 6.374820198 | -0.344393870 |
| C | 6.250348818 | 6.561500755 | -1.211940045 |
| C | 7.285890379 | 5.467413457 | -0.858776886 |
| C | 5.868894468 | 6.441040116 | -2.706684964 |
| C | 6.916797662 | 7.934010270 | -0.998824777 |
| C | 4.509977172 | 7.367640380 | 0.514411535 |
| H | 2.273213961 | 10.422586353 | 2.037240570 |
| H | 1.806208900 | 9.411238300 | 0.655234223 |
| H | 3.484410396 | 9.940611874 | 0.840134792 |
| H | 3.690656075 | 9.458745505 | 3.922476557 |
| H | 4.934376066 | 8.954198599 | 2.768472134 |
| H | 4.224283149 | 7.766581128 | 3.871459769 |
| H | 1.320372454 | 8.813649916 | 3.647047107 |
| H | 1.756034467 | 7.100230658 | 3.650183075 |
| H | 0.769412925 | 7.766366176 | 2.333355665 |
| H | 1.793481436 | 5.795242573 | 1.803170666 |
| H | 0.453803683 | 4.659085880 | 0.190796904 |
| H | -2.039743896 | -1.349034381 | -0.472766144 |
| H | -4.618040167 | 4.978578083 | -0.209053120 |
| H | -6.648864902 | 6.432871524 | -0.391539201 |
| H | -9.665281586 | 1.842299979 | -1.322961093 |
| H | -11.626721669 | 3.358117536 | -1.487292220 |
| H | -11.320710955 | 5.812235830 | -1.198496059 |
| H | -9.052876398 | 6.733450604 | -0.746952213 |
| H | -8.625247681 | -0.117675187 | -1.383893758 |
| H | -7.347779140 | -2.200777904 | -1.416144333 |
| H | -4.926655857 | -2.321650962 | -1.037378404 |
| H | -2.434188675 | 3.677795978 | -0.072802504 |
| H | -0.383505813 | -2.415688548 | 2.094663451 |
| H | -6.224588999 | -4.595594728 | 0.054985529 |
| H | -7.670267270 | -5.975147015 | -1.422413602 |
| H | -6.727234941 | -7.960693133 | -2.586431583 |
| H | -4.342289946 | -8.575739021 | -2.239446669 |
| H | -2.918781312 | -7.227567532 | -0.714668612 |
| H | -2.172143906 | -2.813105467 | 4.175782514 |
| H | -4.072022424 | -3.511743380 | 5.615347867 |
| H | -5.848292433 | -4.987867837 | 4.721419187 |
| H | -5.783374979 | -5.786134004 | 2.374223415 |
| H | -2.167976926 | -4.967938795 | -1.824934356 |
| H | -0.050895269 | -3.692538083 | -1.997821204 |
| H | 2.332784651 | -4.049722672 | -0.237789229 |
| H | 4.151178836 | -4.766347801 | 1.011453180 |
| H | 5.050841708 | -5.841137904 | 2.820503736 |
| H | 6.806863047 | -5.702636660 | 2.986216152 |
| H | 6.019697389 | -7.284979030 | 3.152339332 |
| H | 4.050136812 | -7.043119204 | 0.738097586 |
| H | 5.040999609 | -8.460447121 | 1.113929518 |
| H | 5.136747692 | -7.707473987 | -0.490710604 |
| H | 8.364354989 | -6.774601809 | 1.250788508 |
| H | 7.684120601 | -7.587209779 | -0.174185319 |
| H | 7.481408726 | -8.294810779 | 1.433232493 |
| H | 8.112102227 | -5.383818269 | -0.506080468 |
| H | 9.872879091 | -3.943568938 | -0.531474122 |
| H | 9.464749391 | -2.249835630 | -0.222180708 |
| H | 10.515189197 | -2.677039439 | -1.587715308 |
| H | 8.892201304 | -5.134898217 | -2.632627106 |
| H | 9.555164881 | -3.840357747 | -3.641289706 |
| H | 7.827115407 | -4.237393568 | -3.724234880 |
| H | 8.003460497 | -0.940760936 | -1.879205394 |
| H | 7.340933492 | -1.751489311 | -3.313002910 |
| H | 9.079520260 | -1.458781986 | -3.182793615 |
| H | 5.934915256 | -1.841504848 | -1.541952308 |
| H | 4.752465709 | -0.506195871 | 0.011717121 |
| H | 4.188558282 | 2.487564588 | 0.259342336 |
| H | 4.598767352 | 4.387229540 | -1.093187770 |
| H | 6.891138228 | 4.461040564 | -1.032787850 |
| H | 7.581091855 | 5.532992012 | 0.194750780 |
| H | 8.187791348 | 5.582220737 | -1.472723451 |
| H | 5.427368921 | 5.466173753 | -2.937786258 |
| H | 6.757038945 | 6.564031386 | -3.338944597 |
| H | 5.141845209 | 7.211412598 | -2.988090641 |
| H | 7.236756113 | 8.075895496 | 0.039804448 |
| H | 6.247450779 | 8.759361569 | -1.266955799 |
| H | 7.807545109 | 8.013365372 | -1.632360585 |
| H | 5.045552735 | 8.306923699 | 0.585381218 |

Table S8. The Cartesian coordinates of optmized S_0_, S_1_ and T_1_ geometry for NBisICz-DPA.

|  | **X (Å)** | **Y (Å)** | **Z (Å)** |
| --- | --- | --- | --- |
| ***Optimized S_0_ geometry*** | | | |
| C | 2.532040184 | 8.981871659 | 1.038798075 |
| C | 1.688559124 | 8.036725584 | 1.927522139 |
| C | 2.657300191 | 7.163445538 | 2.747518196 |
| C | 0.849172060 | 8.879019658 | 2.917543212 |
| C | 0.749371056 | 7.198544541 | 1.038250075 |
| C | 0.790135056 | 5.803900442 | 1.005386073 |
| C | -0.084009006 | 5.061884362 | 0.190918014 |
| C | -0.020598001 | 3.573219255 | 0.172673012 |
| C | 1.250800092 | 2.939805212 | 0.137198010 |
| C | 1.325582098 | 1.542326108 | 0.121715009 |
| C | 2.281524166 | 0.436757031 | 0.034867003 |
| C | 1.529228109 | -0.796934058 | 0.020257001 |
| C | 2.144375155 | -2.037729149 | -0.093545007 |
| C | 3.537712256 | -2.062881150 | -0.178383013 |
| C | 4.500815325 | -3.163769227 | -0.302768022 |
| C | 5.728611431 | -2.520523181 | -0.339872024 |
| N | 5.653881391 | -1.162223086 | -0.254545018 |
| C | 7.023452490 | -3.010605217 | -0.449571033 |
| C | 7.847275578 | -1.796700130 | -0.428796031 |
| C | 6.968637506 | -0.682635048 | -0.308098022 |
| C | 7.432773526 | 0.645452048 | -0.258139019 |
| C | 8.790170639 | 0.861543060 | -0.330027024 |
| C | 9.720716713 | -0.211184015 | -0.451645033 |
| C | 9.251005651 | -1.568535115 | -0.502654036 |
| C | 10.205330729 | -2.612089186 | -0.623760046 |
| C | 11.554027850 | -2.336179169 | -0.691542048 |
| C | 12.017931879 | -1.001157073 | -0.641616048 |
| C | 11.117111807 | 0.034061002 | -0.524242038 |
| C | 7.076166535 | -4.414021319 | -0.537030038 |
| C | 5.869253409 | -5.142118370 | -0.505691037 |
| C | 4.588278328 | -4.558990326 | -0.391491028 |
| C | 4.288825311 | -0.829269058 | -0.151880011 |
| C | 3.676598265 | 0.412016030 | -0.048582004 |
| C | 0.096779007 | 0.905040064 | 0.155046011 |
| N | 0.155472011 | -0.461324033 | 0.116256008 |
| C | -1.181316087 | 1.432459101 | 0.165177012 |
| C | -2.038082147 | 0.244271017 | 0.130823010 |
| C | -1.178111082 | -0.914938065 | 0.109722008 |
| C | -1.736257124 | -2.199089156 | 0.101495007 |
| N | -0.935990070 | -3.391389243 | 0.136773010 |
| C | -1.076030076 | -4.269152305 | -0.985614069 |
| C | -0.799290055 | -3.776352272 | -2.269392164 |
| C | -0.959539071 | -4.592805329 | -3.387314245 |
| C | -1.383310102 | -5.915451429 | -3.242683234 |
| C | -1.656479119 | -6.409945449 | -1.966060139 |
| C | -1.514747110 | -5.594350379 | -0.843605062 |
| C | -0.486777035 | -3.872606280 | 1.395388102 |
| C | -0.939369068 | -3.304556236 | 2.598193188 |
| C | -0.443348032 | -3.752801270 | 3.822600273 |
| C | 0.500051036 | -4.777380342 | 3.883470278 |
| C | 0.951388069 | -5.346971392 | 2.690826194 |
| C | 0.474227034 | -4.900743354 | 1.461572104 |
| C | -3.132138223 | -2.304210164 | 0.066210005 |
| C | -3.990840289 | -1.190694087 | 0.059410004 |
| C | -5.466232386 | -1.370401098 | 0.024400002 |
| C | -6.080942459 | -2.398247173 | 0.745732052 |
| C | -7.471948518 | -2.583913186 | 0.725389050 |
| C | -8.098910579 | -3.728159269 | 1.545879110 |
| C | -7.516103544 | -5.080939368 | 1.072043075 |
| C | -7.770792585 | -3.526634253 | 3.044584217 |
| C | -9.630916710 | -3.791115275 | 1.396927103 |
| C | -8.233799606 | -1.698908122 | -0.042719003 |
| C | -7.657947548 | -0.649603047 | -0.782540056 |
| C | -8.564708639 | 0.279635020 | -1.612986115 |
| C | -9.335576696 | -0.553786039 | -2.664362193 |
| C | -9.575482694 | 0.983950071 | -0.676635051 |
| C | -7.767780561 | 1.367389101 | -2.358055171 |
| C | -6.271038432 | -0.503382036 | -0.736280052 |
| C | -3.420608246 | 0.090859007 | 0.103717007 |
| C | -1.231209087 | 2.831728204 | 0.180681013 |
| C | -1.010759074 | 5.749239395 | -0.598578041 |
| C | -1.088960078 | 7.150754491 | -0.594504043 |
| C | -2.123416154 | 7.857597551 | -1.492267105 |
| C | -1.846402135 | 7.504297547 | -2.973078216 |
| C | -3.546132254 | 7.383616505 | -1.111032082 |
| C | -2.078837151 | 9.390979701 | -1.350977097 |
| C | -0.200477014 | 7.848313560 | 0.228690016 |
| H | 3.203192228 | 9.589977706 | 1.657883121 |
| H | 1.904176135 | 9.665720697 | 0.458210033 |
| H | 3.145434225 | 8.410960582 | 0.332265024 |
| H | 3.304065239 | 7.803714554 | 3.358086240 |
| H | 3.305422240 | 6.557942461 | 2.103688151 |
| H | 2.124125151 | 6.489109466 | 3.427100246 |
| H | 1.504882108 | 9.485811704 | 3.554216257 |
| H | 0.246804018 | 8.233739597 | 3.567131256 |
| H | 0.166788012 | 9.559842702 | 2.398159171 |
| H | 1.486654106 | 5.261839378 | 1.634502118 |
| H | 2.140936154 | 3.560053257 | 0.091745006 |
| H | 1.563497114 | -2.949499210 | -0.113561008 |
| H | 6.739016491 | 1.474715105 | -0.165364012 |
| H | 9.175815650 | 1.877199134 | -0.293847021 |
| H | 9.861619734 | -3.640692260 | -0.662975049 |
| H | 12.267659883 | -3.150624226 | -0.784103055 |
| H | 13.083497951 | -0.796213059 | -0.695951052 |
| H | 11.465866814 | 1.063629078 | -0.485116035 |
| H | 8.012175570 | -4.956410356 | -0.627913045 |
| H | 5.929959406 | -6.224933441 | -0.574494041 |
| H | 3.708416269 | -5.195671372 | -0.374046027 |
| H | 4.250397306 | 1.332902095 | -0.039579003 |
| H | -0.460184033 | -2.751111199 | -2.382533170 |
| H | -0.740505051 | -4.194508303 | -4.374406316 |
| H | -1.499776109 | -6.552969465 | -4.114462296 |
| H | -1.997170141 | -7.434327561 | -1.839509130 |
| H | -1.739599127 | -5.982384414 | 0.144613010 |
| H | -1.678490119 | -2.512186179 | 2.577186184 |
| H | -0.809871060 | -3.294102239 | 4.737419342 |
| H | 0.880066063 | -5.124083369 | 4.839885346 |
| H | 1.692890122 | -6.141501451 | 2.712031194 |
| H | 0.842254062 | -5.352059379 | 0.545912040 |
| H | -3.556268258 | -3.302387239 | 0.022077002 |
| H | -5.460122400 | -3.048528218 | 1.354855098 |
| H | -6.428847448 | -5.122449370 | 1.194603087 |
| H | -7.739688552 | -5.254567376 | 0.013208001 |
| H | -7.948156553 | -5.906455416 | 1.651023121 |
| H | -6.691507486 | -3.518201253 | 3.228655232 |
| H | -8.204816568 | -4.337370312 | 3.642734260 |
| H | -8.179320596 | -2.577781186 | 3.410706245 |
| H | -9.933712725 | -3.959390285 | 0.357110026 |
| H | -10.114245724 | -2.873257209 | 1.750581127 |
| H | -10.024955742 | -4.621514332 | 1.993672142 |
| H | -9.309934671 | -1.821078130 | -0.073780005 |
| H | -9.962007727 | -1.321199097 | -2.197878161 |
| H | -8.643202612 | -1.058084078 | -3.348091241 |
| H | -9.990365701 | 0.094357007 | -3.259851236 |
| H | -10.209022745 | 0.266323019 | -0.145107011 |
| H | -10.233285734 | 1.645542120 | -1.253705090 |
| H | -9.056500627 | 1.591057115 | 0.073964005 |
| H | -7.052244495 | 0.936196068 | -3.067291218 |
| H | -7.216725537 | 2.015090147 | -1.666637120 |
| H | -8.455180591 | 2.002666145 | -2.928072209 |
| H | -5.784169433 | 0.273412020 | -1.314319094 |
| H | -4.060554290 | 0.967640071 | 0.137434010 |
| H | -2.173590158 | 3.369675243 | 0.218200016 |
| H | -1.664931122 | 5.174245372 | -1.247477092 |
| H | -1.907052140 | 6.426075459 | -3.153500225 |
| H | -0.846516061 | 7.837083572 | -3.274255235 |
| H | -2.579205186 | 7.994058552 | -3.626322261 |
| H | -3.662351262 | 6.301798447 | -1.234138089 |
| H | -4.294887311 | 7.873284587 | -1.745865125 |
| H | -3.773650272 | 7.627610557 | -0.066978005 |
| H | -1.104693079 | 9.800129708 | -1.642062118 |
| H | -2.293330163 | 9.713098677 | -0.325573024 |
| H | -2.834103205 | 9.843181717 | -2.003747143 |
| H | -0.240433017 | 8.930939619 | 0.248643018 |
| ***Optimized S_1_ geometry*** | | | |
| C | 2.555027840 | 8.951529112 | 1.041937430 |
| C | 1.701718540 | 8.016978965 | 1.932452261 |
| C | 2.661488047 | 7.138983155 | 2.757919726 |
| C | 0.867671260 | 8.870372213 | 2.917442832 |
| C | 0.757387647 | 7.182945878 | 1.044600890 |
| C | 0.789220876 | 5.788139585 | 1.014843565 |
| C | -0.089156195 | 5.048874095 | 0.201993478 |
| C | -0.037537532 | 3.559450857 | 0.188883550 |
| C | 1.239543825 | 2.925993109 | 0.173909276 |
| C | 1.315121200 | 1.517371210 | 0.160398743 |
| C | 2.254173201 | 0.417380227 | 0.059738875 |
| C | 1.499108938 | -0.831520236 | 0.012333341 |
| C | 2.086063775 | -2.063788085 | -0.220917861 |
| C | 3.485269965 | -2.117001261 | -0.348858350 |
| C | 4.422205024 | -3.195188989 | -0.578309150 |
| C | 5.660162037 | -2.562854271 | -0.586773832 |
| N | 5.592412796 | -1.204135564 | -0.391741943 |
| C | 6.948775843 | -3.042167502 | -0.755945015 |
| C | 7.774583664 | -1.842453414 | -0.653717384 |
| C | 6.907926059 | -0.724110209 | -0.428687061 |
| C | 7.383343132 | 0.587293468 | -0.280344838 |
| C | 8.744317695 | 0.802725813 | -0.357475028 |
| C | 9.661904906 | -0.266466831 | -0.581575496 |
| C | 9.179700763 | -1.614673687 | -0.733383326 |
| C | 10.121639706 | -2.651732537 | -0.954512962 |
| C | 11.474694178 | -2.385015890 | -1.024386965 |
| C | 11.949260706 | -1.063706094 | -0.875639220 |
| C | 11.058547444 | -0.031783932 | -0.659393498 |
| C | 6.999319097 | -4.446095196 | -0.957847980 |
| C | 5.785512220 | -5.161990240 | -0.964675737 |
| C | 4.507413118 | -4.593939922 | -0.783938142 |
| C | 4.245262154 | -0.872635271 | -0.242016721 |
| C | 3.650819882 | 0.368804632 | -0.056684944 |
| C | 0.074541106 | 0.899687759 | 0.188317731 |
| N | 0.130479767 | -0.487536748 | 0.147068621 |
| C | -1.196895462 | 1.421291037 | 0.180770170 |
| C | -2.047111689 | 0.239561174 | 0.134926144 |
| C | -1.177786660 | -0.933970248 | 0.166536358 |
| C | -1.754326511 | -2.220997211 | 0.223571101 |
| N | -0.993346248 | -3.394979224 | 0.483801737 |
| C | -1.201953423 | -4.523394234 | -0.354289472 |
| C | -1.219638860 | -4.341132518 | -1.748257324 |
| C | -1.434800197 | -5.428478077 | -2.588578566 |
| C | -1.633402847 | -6.705503696 | -2.055974990 |
| C | -1.621206206 | -6.886597093 | -0.670458817 |
| C | -1.412229824 | -5.805718113 | 0.181826381 |
| C | -0.289548462 | -3.505027544 | 1.705611041 |
| C | -0.671563956 | -2.727739705 | 2.816421456 |
| C | 0.034266599 | -2.826394971 | 4.010401712 |
| C | 1.116127334 | -3.703275682 | 4.127818105 |
| C | 1.491442520 | -4.484642028 | 3.031278394 |
| C | 0.803788163 | -4.387868961 | 1.827727772 |
| C | -3.150579966 | -2.338192863 | 0.091694982 |
| C | -3.993125213 | -1.222486955 | -0.004393703 |
| C | -5.464764290 | -1.387388974 | -0.095514794 |
| C | -6.120137877 | -2.420641827 | 0.583846879 |
| C | -7.509891691 | -2.592324019 | 0.501626775 |
| C | -8.182787977 | -3.739657994 | 1.280479077 |
| C | -7.590833796 | -5.092540505 | 0.818404292 |
| C | -7.920947741 | -3.556463154 | 2.794506681 |
| C | -9.707004302 | -3.787129141 | 1.061872204 |
| C | -8.229753146 | -1.690090854 | -0.287328693 |
| C | -7.613940797 | -0.634927491 | -0.986249986 |
| C | -8.475761564 | 0.313605857 | -1.842041931 |
| C | -9.203562477 | -0.498623210 | -2.939689295 |
| C | -9.522904807 | 1.010549573 | -0.940646935 |
| C | -7.638429004 | 1.406635865 | -2.533047272 |
| C | -6.230168962 | -0.500091886 | -0.875828707 |
| C | -3.419159142 | 0.070111931 | 0.040367795 |
| C | -1.253279388 | 2.838442569 | 0.183214595 |
| C | -1.009443248 | 5.741573310 | -0.590929445 |
| C | -1.077159320 | 7.143409829 | -0.591892177 |
| C | -2.103626898 | 7.854733908 | -1.495408370 |
| C | -1.824579865 | 7.494360286 | -2.974081657 |
| C | -3.531033457 | 7.392471387 | -1.117484531 |
| C | -2.048847523 | 9.388276135 | -1.359536041 |
| C | -0.186093680 | 7.837714986 | 0.231433673 |
| H | 3.230197413 | 9.556524654 | 1.659851597 |
| H | 1.934019494 | 9.638175222 | 0.457193385 |
| H | 3.164814400 | 8.372409225 | 0.339067167 |
| H | 3.311832000 | 7.776236232 | 3.368017378 |
| H | 3.306018369 | 6.525899985 | 2.117904720 |
| H | 2.120951712 | 6.471038116 | 3.438001990 |
| H | 1.527054254 | 9.474462916 | 3.553053538 |
| H | 0.258718699 | 8.232613614 | 3.568424788 |
| H | 0.191769902 | 9.554576065 | 2.393949539 |
| H | 1.483059828 | 5.241775961 | 1.642837194 |
| H | 2.129417119 | 3.544797870 | 0.125894687 |
| H | 1.489731573 | -2.958767829 | -0.331752629 |
| H | 6.698435052 | 1.411797592 | -0.108357774 |
| H | 9.139454558 | 1.808984869 | -0.246159053 |
| H | 9.764735687 | -3.670023126 | -1.070334394 |
| H | 12.178049114 | -3.195948099 | -1.194952678 |
| H | 13.015605198 | -0.861229676 | -0.931957311 |
| H | 11.419878930 | 0.987974416 | -0.544512975 |
| H | 7.930816738 | -4.981625170 | -1.107723243 |
| H | 5.839343252 | -6.236862366 | -1.124076677 |
| H | 3.627722411 | -5.230720226 | -0.814336708 |
| H | 4.241917928 | 1.278172462 | -0.021430249 |
| H | -1.049560371 | -3.350914015 | -2.157694282 |
| H | -1.435562879 | -5.279374417 | -3.664196519 |
| H | -1.797271504 | -7.552464107 | -2.715379235 |
| H | -1.789275403 | -7.873077414 | -0.248114397 |
| H | -1.424457438 | -5.944477542 | 1.257474499 |
| H | -1.527904414 | -2.067677152 | 2.741860069 |
| H | -0.273110718 | -2.223062632 | 4.859467846 |
| H | 1.662899631 | -3.775451723 | 5.062939060 |
| H | 2.340209806 | -5.157579971 | 3.104241618 |
| H | 1.119953201 | -4.978255269 | 0.975452839 |
| H | -3.578240999 | -3.334179563 | 0.099551709 |
| H | -5.534701678 | -3.083414371 | 1.213779873 |
| H | -6.510489073 | -5.145645532 | 0.989492350 |
| H | -7.767987469 | -5.253239872 | -0.251176190 |
| H | -8.055793164 | -5.919932661 | 1.368250157 |
| H | -6.851025269 | -3.560142772 | 3.027672517 |
| H | -8.388992846 | -4.369071252 | 3.363536480 |
| H | -8.337046321 | -2.607759553 | 3.152079881 |
| H | -9.964252126 | -3.940907409 | 0.007673135 |
| H | -10.198085287 | -2.869348748 | 1.404616493 |
| H | -10.134397746 | -4.620693404 | 1.630379774 |
| H | -9.304440859 | -1.801872298 | -0.366372409 |
| H | -9.856046545 | -1.268153518 | -2.514139838 |
| H | -8.484599406 | -0.997336075 | -3.599687717 |
| H | -9.826106543 | 0.163467556 | -3.553767435 |
| H | -10.185861186 | 0.290066750 | -0.450361388 |
| H | -10.148492330 | 1.686208154 | -1.536544085 |
| H | -9.034507438 | 1.601928199 | -0.157759001 |
| H | -6.895259249 | 0.981639011 | -3.217212393 |
| H | -7.114577818 | 2.039799618 | -1.807884551 |
| H | -8.295209620 | 2.055874933 | -3.122705893 |
| H | -5.714759740 | 0.281558405 | -1.421100655 |
| H | -4.065730728 | 0.941974514 | 0.024677491 |
| H | -2.194132734 | 3.376680957 | 0.218746370 |
| H | -1.665748187 | 5.167393553 | -1.238154819 |
| H | -1.891086804 | 6.415796032 | -3.150100330 |
| H | -0.821481220 | 7.819318139 | -3.273012869 |
| H | -2.552013270 | 7.986442474 | -3.631817666 |
| H | -3.653478489 | 6.310826634 | -1.235870975 |
| H | -4.274444305 | 7.884414349 | -1.757087583 |
| H | -3.760320487 | 7.642590559 | -0.075210206 |
| H | -1.070839790 | 9.789290940 | -1.648887457 |
| H | -2.263810079 | 9.715449759 | -0.335803838 |
| H | -2.798903726 | 9.843745079 | -2.016213733 |
| H | -0.217671900 | 8.920791640 | 0.247515635 |
| ***Optimized T_1_ geometry*** | | | |
| C | 2.424286049 | 9.023996920 | 1.030873382 |
| C | 1.594290843 | 8.068744565 | 1.921470765 |
| C | 2.575420378 | 7.207162405 | 2.739108829 |
| C | 0.747270148 | 8.900893199 | 2.913578314 |
| C | 0.663074799 | 7.219351209 | 1.034427013 |
| C | 0.720195059 | 5.825285129 | 1.001790202 |
| C | -0.146828446 | 5.072541960 | 0.189450566 |
| C | -0.066211631 | 3.584740772 | 0.171682134 |
| C | 1.213288421 | 2.966063521 | 0.136817409 |
| C | 1.305104975 | 1.569998009 | 0.122074627 |
| C | 2.275052291 | 0.476313540 | 0.036519118 |
| C | 1.536620493 | -0.769840426 | 0.022283378 |
| C | 2.163558582 | -2.002051754 | -0.090085708 |
| C | 3.560526796 | -2.013889698 | -0.174634856 |
| C | 4.532343378 | -3.097156368 | -0.296795699 |
| C | 5.748818964 | -2.449072542 | -0.334237284 |
| N | 5.658722145 | -1.081933250 | -0.249698560 |
| C | 7.055652714 | -2.937006584 | -0.443586651 |
| C | 7.873161395 | -1.756384952 | -0.426431338 |
| C | 6.958975788 | -0.572380036 | -0.301427111 |
| C | 7.427738881 | 0.707405861 | -0.256986711 |
| C | 8.845014886 | 0.907622920 | -0.334010053 |
| C | 9.769186121 | -0.175444884 | -0.454003524 |
| C | 9.292265602 | -1.536237157 | -0.502270068 |
| C | 10.220650780 | -2.577914396 | -0.619832572 |
| C | 11.618455868 | -2.321880541 | -0.692451984 |
| C | 12.077604974 | -1.022065480 | -0.646713146 |
| C | 11.156859580 | 0.049903478 | -0.527782033 |
| C | 7.118405304 | -4.350634124 | -0.529603200 |
| C | 5.912023049 | -5.083898120 | -0.497065736 |
| C | 4.633033071 | -4.510424560 | -0.384507073 |
| C | 4.295988847 | -0.767829161 | -0.148508711 |
| C | 3.667710315 | 0.468117315 | -0.046361600 |
| C | 0.083908264 | 0.917933526 | 0.155335887 |
| N | 0.158463753 | -0.448346174 | 0.117692848 |
| C | -1.200107367 | 1.430155507 | 0.164989304 |
| C | -2.042616487 | 0.232487320 | 0.131207238 |
| C | -1.168778602 | -0.916967301 | 0.111147183 |
| C | -1.713067751 | -2.207481373 | 0.103137960 |
| N | -0.900073868 | -3.391356931 | 0.139212220 |
| C | -1.023875213 | -4.266798863 | -0.986565233 |
| C | -0.746200189 | -3.766697338 | -2.267362430 |
| C | -0.891403786 | -4.581266952 | -3.388704722 |
| C | -1.300764807 | -5.909152103 | -3.250638446 |
| C | -1.574716259 | -6.410944377 | -1.977021469 |
| C | -1.448043678 | -5.597481256 | -0.851244556 |
| C | -0.450076761 | -3.870194999 | 1.398061505 |
| C | -0.912114196 | -3.308619047 | 2.600402397 |
| C | -0.416119752 | -3.754292248 | 3.825711687 |
| C | 0.536766307 | -4.769979095 | 3.888192052 |
| C | 0.997763253 | -5.332906284 | 2.696078173 |
| C | 0.520680220 | -4.889063529 | 1.465955888 |
| C | -3.107388492 | -2.328619496 | 0.067260515 |
| C | -3.978945776 | -1.224873179 | 0.059532090 |
| C | -5.452105206 | -1.421492034 | 0.023712597 |
| C | -6.055597621 | -2.456687873 | 0.744094953 |
| C | -7.444371663 | -2.658239978 | 0.722897901 |
| C | -8.058578647 | -3.810151622 | 1.542332088 |
| C | -7.459917620 | -5.155850091 | 1.068113601 |
| C | -7.733798915 | -3.605759545 | 3.041369614 |
| C | -9.589660623 | -3.890772363 | 1.392372130 |
| C | -8.216024119 | -1.781533715 | -0.044949243 |
| C | -7.651707437 | -0.725307927 | -0.783808075 |
| C | -8.568575866 | 0.194146613 | -1.614070404 |
| C | -9.330192582 | -0.647308312 | -2.665786553 |
| C | -9.587115703 | 0.887017928 | -0.677593089 |
| C | -7.783657731 | 1.290846300 | -2.358769921 |
| C | -6.266580484 | -0.563391289 | -0.736874139 |
| C | -3.423352173 | 0.062949730 | 0.103509645 |
| C | -1.267264812 | 2.829490832 | 0.179873268 |
| C | -1.083141033 | 5.749026560 | -0.598252840 |
| C | -1.178036987 | 7.149490393 | -0.594146753 |
| C | -2.222895946 | 7.843781580 | -1.489679238 |
| C | -1.945665061 | 7.492941720 | -2.971022462 |
| C | -3.639028167 | 7.353522301 | -1.104536500 |
| C | -2.195800833 | 9.377670100 | -1.349386649 |
| C | -0.296165310 | 7.857817848 | 0.226955531 |
| H | 3.089538110 | 9.640200860 | 1.648365498 |
| H | 1.786862111 | 9.700179238 | 0.451728340 |
| H | 3.042829111 | 8.460487497 | 0.322876115 |
| H | 3.216002726 | 7.855145820 | 3.348099988 |
| H | 3.229036171 | 6.609371663 | 2.093637693 |
| H | 2.052011389 | 6.526486366 | 3.419945235 |
| H | 1.397145028 | 9.515428114 | 3.548846526 |
| H | 0.154170168 | 8.248339439 | 3.564423689 |
| H | 0.055544617 | 9.573520293 | 2.395862796 |
| H | 1.424434620 | 5.291608207 | 1.629456423 |
| H | 2.095942483 | 3.596913553 | 0.090963350 |
| H | 1.592073839 | -2.919770588 | -0.109315975 |
| H | 6.761721423 | 1.558648512 | -0.166156202 |
| H | 9.236265366 | 1.919942104 | -0.300180109 |
| H | 9.870033334 | -3.603715576 | -0.656533189 |
| H | 12.309771544 | -3.154391624 | -0.783265208 |
| H | 13.141571926 | -0.808968638 | -0.700960339 |
| H | 11.524861081 | 1.072173073 | -0.492169068 |
| H | 8.058016502 | -4.885027203 | -0.619219430 |
| H | 5.978677421 | -6.166589215 | -0.564014125 |
| H | 3.755503053 | -5.149558322 | -0.365587405 |
| H | 4.231717634 | 1.395239562 | -0.038228278 |
| H | -0.417978950 | -2.737395925 | -2.375320733 |
| H | -0.671908979 | -4.177189332 | -4.373359381 |
| H | -1.405596619 | -6.545044930 | -4.125098140 |
| H | -1.904403301 | -7.439552234 | -1.855536645 |
| H | -1.673604670 | -5.991261044 | 0.134541814 |
| H | -1.658430047 | -2.523083933 | 2.578100026 |
| H | -0.790148217 | -3.300619962 | 4.740017327 |
| H | 0.916661268 | -5.114810374 | 4.845353356 |
| H | 1.746878198 | -6.120273399 | 2.718420957 |
| H | 0.896595205 | -5.334852810 | 0.550816376 |
| H | -3.519983924 | -3.331649487 | 0.023337954 |
| H | -5.427692460 | -3.100215018 | 1.353131796 |
| H | -6.372316501 | -5.184779359 | 1.191142178 |
| H | -7.680952629 | -5.331479517 | 0.009068767 |
| H | -7.882658327 | -5.986646513 | 1.646446315 |
| H | -6.654805287 | -3.584846185 | 3.226108376 |
| H | -8.158759150 | -4.421830408 | 3.638804341 |
| H | -8.153540880 | -2.661908399 | 3.407745360 |
| H | -9.889793712 | -4.061694868 | 0.352213578 |
| H | -10.083815228 | -2.978835299 | 1.746386222 |
| H | -9.974454399 | -4.726149502 | 1.988227951 |
| H | -9.290691775 | -1.915939878 | -0.076631757 |
| H | -9.948258697 | -1.421656275 | -2.199597812 |
| H | -8.632259650 | -1.143837950 | -3.349552786 |
| H | -9.991949899 | -0.006183173 | -3.261194833 |
| H | -10.212753657 | 0.162285735 | -0.146326738 |
| H | -10.252106664 | 1.541581196 | -1.254470333 |
| H | -9.074837994 | 1.499497534 | 0.073243815 |
| H | -7.063236247 | 0.867734957 | -3.067919611 |
| H | -7.239897151 | 1.944448588 | -1.667144294 |
| H | -8.477972149 | 1.918584542 | -2.928791118 |
| H | -5.788296486 | 0.219190177 | -1.314259292 |
| H | -4.073256777 | 0.932438915 | 0.136492229 |
| H | -2.216274526 | 3.355632773 | 0.217619622 |
| H | -1.731734484 | 5.166139360 | -1.245686980 |
| H | -1.994179258 | 6.413990691 | -3.150676921 |
| H | -0.950484399 | 7.837170039 | -3.274940112 |
| H | -2.685813594 | 7.973772701 | -3.622649221 |
| H | -3.742853341 | 6.270343199 | -1.226645484 |
| H | -4.395137565 | 7.834038527 | -1.737681833 |
| H | -3.866622036 | 7.595433987 | -0.060010240 |
| H | -1.227090078 | 9.797873349 | -1.642877162 |
| H | -2.411656826 | 9.697899595 | -0.323674501 |
| H | -2.957753073 | 9.820709411 | -2.000693864 |
| H | -0.348904401 | 8.939905469 | 0.246832529 |

**Supplementary references**

[S1] C. Maeda, K. Nagahata, T. Ema, *Org. Biomol. Chem.* **2017**, *15* (37), 7783.

[S2] Frisch, M., et al., Wallingford, CT (2016), <https://gaussian.com/citation>.

[S3] A. D. Becke, *J. Chem. Phys.* **1992**, *96* (3), 2155.

[S4] C. Lee, W. Yang, R. G. Parr, *Phys. Rev. B* **1988**, *37* (2), 785.

[S5] S. H. Vosko, L. Wilk, M. Nusair, *Can. J. Phys.* **1980**, *58* (8), 1200.

[S6] P. J. Stephens, F. J. Devlin, C. F. Chabalowski, M. J. Frisch, *J. Phys. Chem.* **1994**, *98* (45), 11623.

[S7] F. Neese, F. Wennmohs, U. Becker, C. Riplinger, *J. Chem. Phys.* **2020**, *152* (22).

[S8] F. Weigend, R. Ahlrichs, Phys. *Chem. Chem. Phys.* **2005**, *7* (18), 3297.

[S9] T. Lu, F. Chen, *J. Comput. Chem.* **2012**, *33* (5), 580.

[S10] J. Liu, T.-L. Lam, M.-K. Sit, Q. Wan, C. Yang, G. Cheng, C.-M. Che, *J. Mater. Chem. C* **2022**, *10* (28), 10271.

[S11] S. Nam, J. W. Kim, H. J. Bae, Y. M. Maruyama, D. Jeong, J. Kim, J. S. Kim, W. J. Son, H. Jeong, J. Lee, *Adv. Sci.* **2021**, *8* (16), 2100586.

[S12] H.-G. Kim, H. Shin, Y. H. Ha, R. Kim, S.-K. Kwon, Y.-H. Kim, J.-J. Kim, *ACS Appl. Mater. Interfaces* **2018**, *11* (1), 26.

[S13] S. H. Baek, J. Y. Park, S. J. Woo, W. S. Lee, W. S. Kim, H. J. Cheon, Y. H. Kim, J. H. Lee, *Small Structures* **2024**, 2300564.

[S14] K. Cheong, S. W. Han, J. Y. Lee, *Small Methods* **2024**, 2301710.

[S15] J. Yan, P. Yi, I.-C. Peng, W.-Y. Hung, B. Hu, G. Ni, S.-M. Yiu, Y. Chi, K. C. LAU, *Inorg. Chem. Front.* **2024**, <https://doi.org/10.1039/D4QI00454J>.

[S16] J. Yan, T. Nakamura, X. Tan, S.-M. Yiu, R. Mimura, K. Hoshi, X. Zhou, Y. Chi, H. Sasabe, J. Kido, *Chem. Eng. J.* **2024**, 150791.

[S17] J. Yan, Z.-H. Qu, D.-Y. Zhou, S.-M. Yiu, Y. Qin, X. Zhou, L.-S. Liao, Y. Chi, *ACS Appl. Mater. Interfaces* **2024**, *16* (3), 3809.

[S18] J. Yan, C. Wu, K. N. Tong, F. Zhou, Y. Chen, Y. Pan, G. Xie, Y. Chi, K. C. Lau, G. Wei, *Small Methods* **2024**, 2301555.

[S19] J. Yan, Z. Q. Feng, Y. Wu, D. Y. Zhou, S. M. Yiu, C. Y. Chan, Y. Pan, K. C. Lau, L. S. Liao, Y. Chi, *Adv. Mater.* **2024**, 2305273.

[S20] K. Cheong, U. Jo, W. P. Hong, J. Y. Lee, *Small Methods* **2023**, 2300862.

[S21] Z. Zheng, L. Wang, Y. Xin, Q. Wang, X. Hong, Y. Zhang, S. M. Yiu, F. Zhou, J. Yan, D. Zhang, *Adv. Funct. Mater.* **2024**, 2311692.

[S22] C. Wu, K. N. Tong, K. Shi, Z. Jin, Y. Wu, Y. Mu, Y. Huo, M. C. Tang, C. Yang, H. Meng, *Adv. Sci.* **2023**, *10* (29), 2301112.

[S23] J. Yan, S. F. Wang, C.-H. Hsu, E. H.-C. Shi, C.-C. Wu, P.-T. Chou, S.-M. Yiu, Y. Chi, C. You, I.-C. Peng, *ACS Appl. Mater. Interfaces* **2023**, *15* (17), 21333.

[S24] Y. Qin, X. Yang, J. Jin, D. Li, X. Zhou, Z. Zheng, Y. Sun, W. Y. Wong, Y. Chi, S. J. Su, *Adv. Opt. Mater.* **2022**, *10* (23), 2201633.

[S25] C. Wu, M. Wang, K. N. Tong, M. Zhang, W. Li, Z. Xu, W. L. Zhang, Y. Wu, C. Yang, H. Y. Fu, *Adv. Opt. Mater.* **2023**, *11* (1), 2201998.

[S26] X. Yang, X. Zhou, Y. X. Zhang, D. Li, C. Li, C. You, T. C. Chou, S. J. Su, P. T. Chou, Y. Chi, *Adv. Sci.* **2022**, *9* (25), 2201150.

[S27] C. You, X.-Q. Wang, X. Zhou, Y. Yuan, L.-S. Liao, Y.-C. Liao, P.-T. Chou, Y. Chi, *ACS Appl. Mater. Interfaces* **2021**, *13* (49), 59023.

[S28] K. H. Lee, J. Y. Lee, *J. Mater. Chem. C* **2019**, *7* (28), 8562.
